# The Effect of Three Complexes of Iodine with Amino Acids on Gene Expression of Model Antibiotic Resistant Microorganisms *Escherichia coli* ATCC BAA-196 and *Staphylococcus aureus* ATCC BAA-39

**DOI:** 10.3390/microorganisms11071705

**Published:** 2023-06-29

**Authors:** Sabina T. Kenesheva, Setshaba Taukobong, Sergey V. Shilov, Tatyana V. Kuznetsova, Ardak B. Jumagaziyeva, Tatyana A. Karpenyuk, Oleg N. Reva, Aleksandr I. Ilin

**Affiliations:** 1 Scientific Center for Anti-Infectious Drugs, Almaty 050060, Kazakhstan; 2 Faculty of Biology and Biotechnology, Al-Farabi Kazakh National University, Almaty 050040, Kazakhstan; 3 Centre for Bioinformatics and Computational Biology, Department of Biochemistry, Genetics and Microbiology, University of Pretoria, Pretoria 0002, South Africa

**Keywords:** RNA sequencing, transcriptomics, antibiotic resistance, *Escherichia coli*, *Staphylococcus aureus*, iodine, amino acids

## Abstract

1. Background: Iodine is a broad-spectrum antimicrobial disinfectant for topical application. Recent studies have shown promising results on the applicability of an iodine-containing complex, FS-1, against antibiotic-resistant pathogens. It was hypothesized that the antimicrobial activity of iodine-containing complexes may be modulated by the organic moiety of the complex, i.e., amino acids. 2. Methods: Gene regulation and metabolic alterations were studied in two model multidrug-resistant microorganisms, *Staphylococcus aureus* ATCC BAA-39, and *Escherichia coli* ATCC BAA-196, treated with three complexes containing iodine and three different amino acids: glycine, L-alanine, and L-isoleucine. The bacterial cultures were exposed to sub-lethal concentrations of the complexes in the lagging and logarithmic growth phases. Gene regulation was studied by total RNA sequencing and differential gene expression analysis. 3. Results: The central metabolism of the treated bacteria was affected. An analysis of the regulation of genes involved in stress responses suggested the disruption of cell wall integrity, DNA damage, and oxidative stress in the treated bacteria. 4. Conclusions: Previous studies showed that the application of iodine-containing complexes, such as FS-1, serves as a supplement to common antibiotics and can be a promising way to combat antibiotic-resistant pathogens. Current results shed light on possible mechanisms of this action by disrupting the cell wall barriers and imposing oxidative stress. It was also found that the effect of the complexes on metabolic pathways varied in the tested microorganisms depending on the organic moiety of the complexes and the growth phase when the complexes had been applied.

## 1. Introduction

Although existing antimicrobial compounds are continuously being modified and new antibiotic classes are being discovered, the rate of resistance continues to rise; therefore, the rate of new antibiotic discovery and production has started to drop substantially [1,2]. The discovery of new groups of antibiotics only temporarily mitigates the problem of drug resistance as the introduction of new antibiotics into the medical practice immediately stimulates the fast selection and propagation of pathogens resistant to the new antibiotic. For this reason, alternative strategies for developing new antimicrobials and supplementary drugs that prevent the acquisition of antibiotic resistance and the distribution of multidrug-resistant pathogens need to be prioritized, along with the discovery of new antibiotics [3,4,5].

The iodine-containing complex FS-1, initially developed as a supplementary medicine against multidrug-resistant tuberculosis [6], has also shown the potential to reverse the antibiotic resistance of multidrug-resistant bacteria to antibiotic-susceptible phenotypes lasting at least for a few generations. This effect was demonstrated using three model multidrug-resistant microorganisms: *Staphylococcus aureus* ATCC BAA-39, *Escherichia coli* ATCC BAA-196, and *Acinetobacter baumanni* ATCC-1790 [7,8,9].

Iodine is a well-known antimicrobial agent that has been used for many years as a disinfectant and antiseptic. When iodine is applied in high concentrations in disinfectants and sanitizers such as povidone-iodine with 10,000 ppm or 1% total titratable iodine, it disrupts cell wall integrity by oxidizing C=C double bonds of unsaturated fatty acids. Iodine slows down or completely halts protein synthesis in bacterial cells, destroys hydrogen bonds in proteins, disrupts activities of enzymes by oxidizing sulfur in cysteine and methionine residues, disrupts electron transport, and causes DNA denaturation and membrane destabilization, which culminates in cell death [10,11]. However, it remains unclear what are the mechanisms of the therapeutic effect of low concentrations of iodine and iodine-containing compounds, such as in the case of the drug FS-1 applied in therapeutic doses of 2.5–4.0 mg/kg [6]. Since iodine can penetrate cell membranes, its application is possible against systemic infections caused by intracellular bacteria such as *Mycobacterium tuberculosis* [6]. However, low toxicity of the drug FS-1 was shown in experiments on rats and dogs [12] and in clinical trials (see www.clinicaltrials.gov, accessed on 26 June 2023; acc. NCT02607449). Considering these factors, complexes of iodine with organic molecules have been shown as a viable therapeutic option to overcome the antibiotic resistance problem [13].

Aqueous iodine solutions are generally unstable and difficult to study due to halogenation by iodine, the large spectrum of organic compounds present in the solution, and the complexes made with them in a random manner [14]. Complexes of iodine with organic compounds, where iodine or iodide ions are fixed by coordination bonds with organic macromolecules, are more stable and less toxic than the solutions of molecular iodine and potassium iodide [15]. The therapeutic activity of an iodine-containing complex FS-1 has been demonstrated in previous studies [7,8,9], where it was hypothesized that the antimicrobial activity and the ability to induce the antibiotic resistance reversion may be modulated by the organic components of the iodine-containing nano-micelles; however, no information was found in the literature to support or falsify this finding. FS-1 is a rather complex construct of dextrin-oligopeptide ligands, iodine, triiodide, and metal ions [8], making it difficult to study the impact of the individual components on the specific activity of the drug. Complexes of iodine with three different amino acids, L-alanine (KS25), glycine (KS33), and L-isoleucine (KS51), were used in this study. The idea of this study was to verify the hypothesis that organic compounds of the complexes can moderate the antimicrobial activity of iodine. To the best of our knowledge, as there are no publications on this issue, we decided to start our study with these common ambivalent and hydrophobic amino acids having no other chemically active or chargeable functional groups in their R-chains, to avoid overcomplicating possible interactions. This initial study will create a background for further studies of complexes of iodine with various organic compounds, including other amino acids, polypeptides, organic acids, carbohydrates, etc. The chemical structures of these three complexes of iodine with L-alanine (KS25), glycine (KS33), and L-isoleucine (KS51) were deposited at the CCDC database (https://www.ccdc.cam.ac.uk/, accessed on 26 June 2023) under accession numbers 1,036,607, 1,036,667 and 1,436,137, respectively. The analysis of the structures showed different distributions of molecular I_2_ and the two anionic states of iodine I_3_^−^ and I^−^ that eventually may affect their antimicrobial activities. These simple ambivalent and hydrophobic amino acids having no extra charges or active functional groups were selected to prove the concept that making complexes with organic molecules may modulate the biological activity of iodine and identify genes and systems in taxonomically distant pathogens, which respond similarly or dissimilarly to the different iodine-containing complexes. The presented work will create the groundwork for further studies on the optimization of organic moieties of iodine-containing complexes designed against different multidrug-resistant pathogens.

## 2. Materials and Methods

### 2.1. Iodine-Containing Compounds

Complexes of iodine linked by coordination bonds to the selected amino acids were synthesized in the Laboratory of New Materials of the Scientific Center for Anti-infectious Drugs (Almaty, Kazakhstan).

Molecular iodine (4.54 g; 0.018 M) was solved in 50 mL 0.048 M iodide salt solution. The resulting solution was mixed with aliquots of the amino acid solutions. The mixture was stirred until completely dissolved and left in a dark place at room temperature for 48 h. Thereafter, the complexes were desiccated till forming crystals in the desiccator (Merk, Manama, Bahrain) filled with anhydrous calcium chloride. The content of the complexes is shown in Table 1.

More specifically, the complexes were prepared as follows.

KS25: 4.54 g of iodine (0.018 mol) was dissolved in 50 mL of water. Thereafter, 20 mL of L-alanine solution (1.79 M) was added to the prepared iodine solution.

KS33: 10 mL of iodine solution containing 1.78 g of lithium iodide (0.013 mol) and 3.384 g (0.013 mol) was mixed with 15 mL glycine solution (0.89 M) preheated at 40 °C for 10 min.

KS51: 1 g of L-isoleucine (0.008 mol) was dissolved in 15 mL of water and heated to 50 °C for 10 min. Then 10 mL of water solution was prepared with 1.02 g (0.008 mol) of lithium iodide and 1.93 g of iodine (0.008 mol). The two solutions were mixed and stirred until completely dissolved.

The concentration of halogens (iodine) in the complexes was measured by sodium thiosulfate titration [16]. The concentration of molecular iodine per 1000 g of the complex was measured by Equation (1):

(1) $$C_{I_{2}}=\frac{V_{1}\cdot K_{1}\cdot 12.69}{m}$$

where *V*_1_—volume of 0.05 M sodium thiosulfate spend for the complete titration; *K*_1_—correction on sodium thiosulfate concentration in the buffer, for 0.05 M solution *K*_1_ = 0.5; and *m*—weight of the complex in g.

The concentration of LiI was measured by Equation (2) using titration with silver nitrate [17]:

(2) $$C_{LiI}=\frac{(V_{2}\cdot K_{2}-V_{1}\cdot K_{1})\cdot 13.39}{m}$$

where *V*_1_—volume of 0.05 M sodium thiosulfate spend for the complete titration; *V*_2_—volume of 0.05 M AgNO_3_ spent for the complete titration; and *K*_1_ = *K*_2_ = 0.5, *m*—weight of the complex in g.

### 2.2. Test Strains and Growth Conditions

Multidrug-resistant strains *Staphylococcus aureus* subsp. *aureus* ATCC^®^ BAA-39™ and *Escherichia coli* ATCC^®^ BAA-196™ were obtained from the American Type Culture Collection (ATCC) and used as the model organisms in this study. Whole genome sequences of these multidrug-resistant strains of bacterial pathogens were obtained and characterized in previous studies [7,8]. Stock cultures were kept in a freezer at −80 °C. Bacteria were cultivated on Mueller–Hinton (MH) liquid or solid media (Himedia, India) without antibiotics (*S. aureus*) and in the medium supplemented with ceftazidime 10 μg/mL (*E. coli*) as recommended in the ATCC product sheet. Before use in the experiments, the strains were twice passaged on MH medium. Agar plates were incubated at 37 °C for 18–24 h. Suspensions were cultivated for 18–24 h in 100 mL flasks with screwed lids in a thermal shaker at 37 °C on 100 rpm.

### 2.3. X-ray Diffractions Analysis

Single crystal X-ray diffraction analysis was conducted on an Auto-Refractometer CAD4 Enraf-Nonius (Rotterdam, The Netherland) using the pre-installed CAD4 version 5 software. The diffraction analysis was run at 200 ± 1 °K to avoid the destruction of the crystals. Fourier transforms were used to identify the coordinates of the atoms with further adjustments for the heavy atoms using the Patterson and superflip functions. Finally, an isotropic refinement of the thermal motions of the hydrogen atoms was applied, whereas a refinement of all other atoms was carried out anisotropically. SHELX, JANA2006, and PLATON v.1.6 software packages [18] were used for structural predictions. The spatial distribution of the atoms in the crystal structures of the complexes was visualized using VESTA Ver. 3.5.7 (https://jp-minerals.org/vesta/en/download.html, accessed on 26 June 2023). Then this information was used for structure modeling and predicting non-covalent bonds using Discovery Studio 4.0. Several additional characteristics of the complexes obtained by the X-ray diffraction method are given in Supplementary Table S1.

### 2.4. UV Spectroscopy

UV spectroscopy was performed using the absorption double beam UV spectrophotometer Lambda 35 Perkin Elmer. Monochromic light emission in a diapason of wavelengths from 190 to 1100 nm was used to record the light abortion by water solutions of the complexes placed in 1.0 cm cuvettes at room temperature. Absorption was measured in a diapason of wavelengths from 190 to 500 nm.

### 2.5. Determination of Minimal Bactericidal Concentrations of the Iodine Containing Complexes

The minimal bactericidal concentration (MBC) was determined by a two-fold microdilution method. The first wells (A-H) of the 96-well plates (BIOLOGIX, Jinan, Shandong, China) were filled with 0.1 mL MH broth supplemented with 0.1 mL aliquots of iodine-containing compounds dissolved in 0.9% NaCl solution to the initial concentration of 8 mg/mL. Thereafter, 0.01 mL bacterial suspensions were prepared from 24 h cultures and, adjusted by saline to 10^6^ CFU/mL, were added into each well of the serial two-fold dilutions. After 24 h incubation, 0.01 mL suspensions from each well were streak-plated onto a solid MH nutrient medium. The MBC values shown in Table 2 were determined as the lowest concentrations of the complexes inhibiting culture growth after 24 h of incubation.

### 2.6. Treatment of the Bacterial Cultures with the Complexes

Aliquots of overnight bacterial culture suspensions from individual colonies adjusted by saline to 1.5–2.1 × 10^9^ CFU mL^−1^ were inoculated into 10 mL liquid MH medium and cultivated with shaking until the lag and mid-log growth phases. In particular, *E. coli* culture was incubated for 1 h (the end of the lagging growth phase, termed the Lag-experiment) and for 6 h (the mid of the logarithmic growth phase, the Log-experiment); *S. aureus* respectively was cultivated for 2.5 h (Lag-experiment) and for 9 h (Log-experiment). The Lag and Log growth phases were estimated based on the strain-specific growth curves on the MH medium, which were determined in the previous study [9]. When the cultures reached the end of the lagging and the mid-logarithmic phases, the iodine-containing complexes, KS25, KS33, and KS51, were added to the growth media in concentrations corresponding to ½ MBC of each complex. The estimated MBC was 2 mg/mL for KS25 and 1 mg/mL, respectively, for KS33 and KS51, for both *E. coli* ATCC BAA-196 and *S. aureus* ATCC BAA-39. The same volume of saline was added to the bacterial cultures in the negative controls. After the addition of the aliquots of the iodine-containing complexes, the samples were incubated for 10 min at 37 °C with shaking (100 rpm). Metabolic processes were stopped by adding the killing buffer (2.0 mL of 1 M Tris-HCl, pH 7.5; 0.5 mL of 1 M MgCl_2_, 1.3 g of NaN_3_; 997.5 mL of water) in the volume ratio 1:1 [19], followed by an immediate RNA extraction. Bacterial cells were collected for RNA extraction by centrifugation at 5000× *g* for 10 min. All the experiments were performed in several repetitions to achieve the necessary amount of generated RNA reads (Table 3).

### 2.7. RNA Library Preparation and Sequencing

Total RNA samples were extracted from the cultures with the use of the RiboPure Bacteria Kit (Ambion, Vilnius, Lithuania) as instructed by the developer’s guidelines. Afterwards, the quantity and quality of the extracted RNA were verified with the use of the NanoDrop 2000c spectrophotometer (Thermo Scientific, Waltham, MA, USA) at optical wavelengths of 260 and 280 nm. Purification of the total RNA from ribosomal RNA was carried out using the MICROBExpress Bacterial mRNA Purification Kit (Ambion, Vilnus, Lithuania) following the developer’s recommendations. Thereafter, the quality of the sample purification was verified by the Bioanalyzer 2100 (Agilent Technologies, Böblingen, Germany) with the RNA 6000 Nano LabChip Kit (Agilent Technologies, Böblingen, Germany). The library preparation from the extracted RNA samples included an enzymatic fragmentation step that was performed using the Ion Total RNA Seq kit V2. Thereafter, the Ion Xpress RNA-Seq Barcode 01-16 Kit was used for barcoding the RNA fragment. RNA sequencing was then performed using the Ion 318 Chip Kit V2 on the Ion Torrent PGM sequencer (Life Technologies, Carlsbad, CA, USA). The generated RNA sequences were quality controlled by removing short reads smaller than 30 bp and those with average base call quality values below 21. The remaining reads were trimmed by the Trimmomatic algorithm to remove low-quality termini and whole reads with average base call quality values below 21. The reads were also trimmed from the adapter sequences. All these steps were performed by using the standard RNA quality control pipeline implemented in UGENE v.36 [20].

### 2.8. Differential Gene Expression Analyses

Differential gene expression was calculated using the R-3.4.4 software packages. Firstly, reference indices were built for each reference genome using the buildindex function available in the Rsubreads package (Bioconducter, www.bioconductor.org, accessed on 26 June 2023). For each bacterium, the obtained RNA fragments were aligned to the relevant reference genomes in FASTA formats (*S. aureus* ATCC BAA-39 NC [CP033505.1] and *E. coli* ATCC BAA-196 NC [CP042865.1 for chromosome and CP042866.1 for plasmid]. Resulting read alignment files in BAM format were sorted using the Samtools-1.10 software package available from htslib (www.htslib.org, accessed on 26 June 2023), and then counts of reads overlapping predicted gene locations were calculated using the featureCounts function of the Bioconducter Rsubread package and the relevant genome annotation files in GFF format. DESeq2 and GenomicFeatures Bioconducter tools were then used for the normalization of the read counts and the calculation of differential gene expression statistics.

### 2.9. Metabolic Pathway and Regulatory Network Analyses

Metabolic pathways and reactions controlled by the differentially expressed genes were identified using the Pathway Tools v.26.0 software [21] and the KEGG Pathway database (https://www.genome.jp/kegg/pathway.html, accessed on 26 June 2023). Gene ontology (GO) and metabolic pathway enrichment analyses were performed using the Pathway Tools Smart table functions. Pairs of homologous genes shared by *S. aureus* BAA-39 and *E. coli* BAA-196 were identified using the program GET_HOMOLOGUES with the parameters set by default [22]. Large, admixed clusters of homologous phage-related integrases and transposases were excluded from the comparison of gene regulation.

### 2.10. Statistical Evaluations

All experiments on RNA sequencing were performed in several repetitions, as shown in Table 3. Genes showing two-fold or greater expression differences and estimated *p*-value equal or smaller than 0.05 were considered as significantly regulated. The gene expression fold-change comparison and visualization of the results were performed using an in-house Python 2.7 script available from the SeqWord project website http://seqword.bi.up.ac.za/transcriptomics_scripts/, accessed on 26 June 2023. Pearson correlation coefficients (Cp) of gene co-regulation were calculated by Equation (3):

(3) $$C_{P}=\frac{N\sum x_{i}y_{i}-\sum x_{i}\sum y_{i}}{\sqrt{\left(N\sum x_{i}^{2}-{\left(\sum x_{i}\right)}^{2}\right)\left(N\sum y_{i}^{2}-{(\sum y_{i})}^{2}\right)}}$$

where *x_i_* and *y_i_* are fold-change values estimated for every *i*’s pair of homologous genes, and *N*—the total number of shared homologous genes.

Grouping of co-expressed genes was performed by using the principle coordinates analysis (PCoA) algorithm implemented in the program PAleontological STatistics (PAST) 4.02 (http://folk.uio.no/ohammer/past/, accessed on 26 June 2023) [23].

## 3. Results

### 3.1. Prediction of Chemical Structures of the Synthesized Complexes

**Figure 1:**
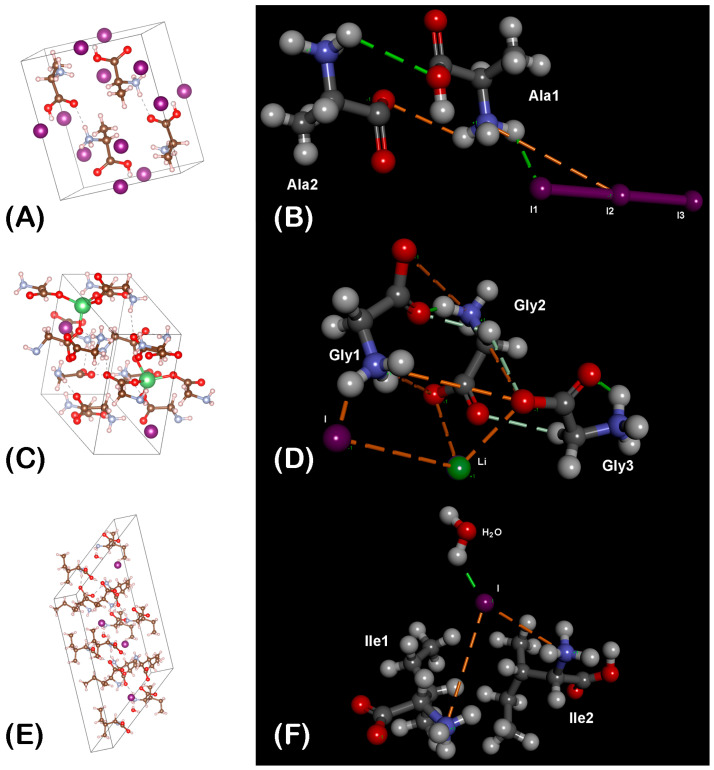
Structural models of the complexes KS25 (**A**,**B**); KS33 (**C**,**D**); and KS51 (**E**,**F**). The distribution of atoms predicted by X-ray diffraction as deposited at the CCDC database are shown in sections (**A**,**C**,**E**). Elementary units of the crystals with predicted non-covalent bonds are shown in sections (**B**,**D**,**F**). Hydrogen, carbon, oxygen, nitrogen, iodine, and lithium atoms are depicted as light grey, dark grey, red, blue, violet, and green colored balls, respectively. The solid edges represent covalent bonds. The dashed edges represent hydrogen bonds (green lines) and electrostatic or metal ion coordination bonds (orange lines).

Three complexes of iodine with amino acids glycine, L-alanine, and L-isoleucine were synthesized. Monoclinic crystals of these complexes: KS25, KS33, and KS51 were obtained and analyzed by X-ray diffraction analysis (Figure 1A,C,E, respectively). It was revealed that the elementary unit of the complex KS25 was a dimer of two molecules of alanine linked by their α-carboxyl and α-amino groups (Figure 1B). The nitrogen atoms in this complex are fully protonated and have positive charges, whereas the iodine atoms form a triiodite I_3_ with a negative charge. The general chemical formula of the complex is 2C_3_H_7_NO_2_ · HI_3_.

The elementary unit of the complex KS33 is a trimer of three molecules of glycine incorporating one lithium cation and one iodine anion (Figure 1D). The lithium-ion form coordination links with the α-carboxyl groups of the two molecules of glycine and with the iodine anion forming a tetrahedral 3D structure. The iodine ion is also linked to the third molecule of glycine by a hydrogen bond. The general chemical formula of the complex is 3C_2_H_5_NO_2_ · LiI.

The elementary unit of the complex KS51 is built up of two molecules of isoleucine linked by their positively charged α-amino groups through an iodine anion, which also has a hydrogen link with one molecule of water (Figure 1F). Only one α-carboxyl group in the complex is deprotonated and has a negative charge that compensates the positive charge of one α-amino group. The positive charge of the second α-amino group is compensated by the iodine anion, whereas one α-carboxyl group remains deprotonated and negatively charged. The general chemical formula of the complex is 4C_6_H_13_NO_2_ · 2HI · H_2_O.

Several other technical parameters of the crystal structures and refinement procedures are given in Supplementary Table S1.

**Figure 2:**
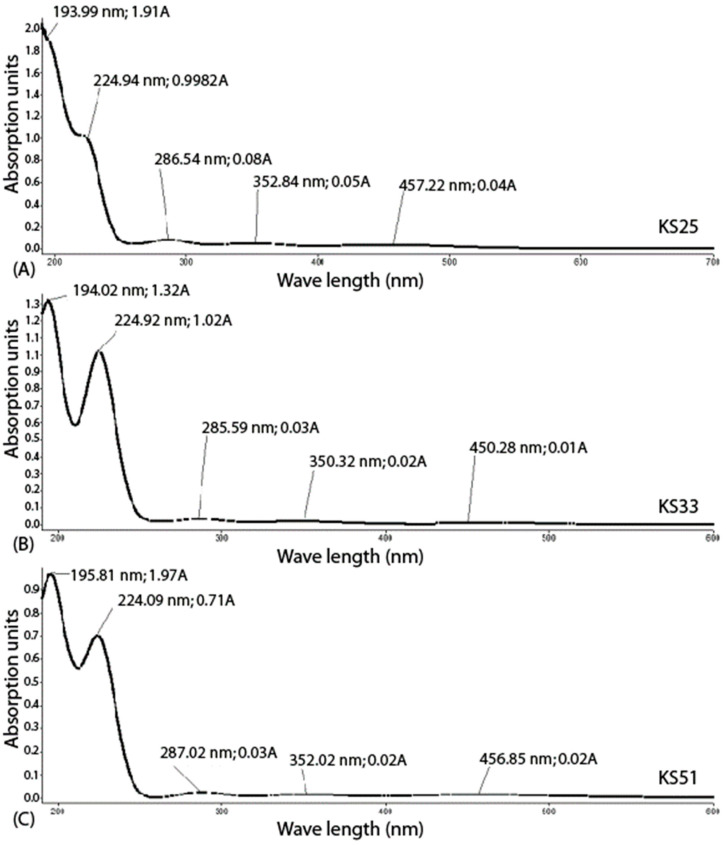
UV-spectra of water solutions of the complexes (**A**) KS25; (**B**) KS33; and (**C**) KD51. Several peaks of absorption bands were pointed out with indication of the respective wavelength in nm and absorption units A.

UV-spectroscopy contributed to the structural modeling of the complexes by providing additional information regarding the creation of transient chemical links and atomic states, which exist in water solutions (Figure 2).

It was observed that the addition of molecular iodine to the solutions KS33 and KS51 in equimolar concentrations of iodine ions led to the formation of triiodide ions: I_2_ + I^−^ ⇄ I_3_^−^. Water solutions of the created complexes were characterized by a dynamic equilibrium of the following molecular species of the complexes KS25, KS33, and KS51, as shown respectively in Equations (4)–(6):

KS25: 2AlaHI_3_ ⇄ Ala_2_ · H^+^ + I_3_^−^ + I_2_ +I^−^ (4)

KS33: 3Gly · LiI + I_2_ ⇄ 3Gly · Li^+^ + I_3_^−^ + I^−^ + I_2_ (5)

KS51: 4Ile · 2H^+^ · 2I^−^ · H_2_O +2I_2_ ⇄ 4Ile · 2H^+^ 2I_3_ + 2I^−^ +2I_2_ +H_2_O. (6)

The UV spectra confirmed that the solutions of these three complexes contained the same three forms of active iodine (I_3_^−^, I_2_, and I^−^). In particular, the UV-absorption peaks at 224–225 nm corresponded to coordination bonds between molecular iodine and α-carboxyl groups of amino acids [24,25]. These peaks were higher in the samples with KS33 and KS51 loaded with molecular iodine (Figure 2B,C). Triodide ions also participated in the creation of complexes with amino acids, which existed in the solution in zwitterionic forms at neutral pH. The absorption peaks at 285–287 nm revealed by UV-spectrometry correspond to ionic bridges between triiodide ions and protonated α-amino groups of amino acids [26]. These peaks were present in all three complexes. The absorption bands at the areas 224–225 nm and 285–287 nm may also be contributed by interactions between molecular iodine with LiI [24,25] that were predicted for KS33 by X-ray crystallography (Figure 1D). Negative charges of iodine ions and triiodides were compensated in the solutions by Li^+^ cations and/or protons. The difference of action of the complexes on gene expression of the bacterial test cultures may be explained by a moderating effect of either the organic component of the complexes (different amino acids, see Table 1) or the cation composition of the complexes (H^+^, Li^+^).

### 3.2. Comparison of Gene Expression Patterns in E. coli and S. aureus under the Effect of Iodine-Containing Complexes

To compare the effects of the iodine-containing complexes on the two model organisms, *E. coli* BAA-196 and *S. aureus* BAA-39, pairs of homologous genes shared by these two genomes were identified using the program GET-HOMOLOGOUS. In total, 725 pairs of homologous genes were found and their expression was analyzed (Supplementary Table S2).

**Figure 3:**
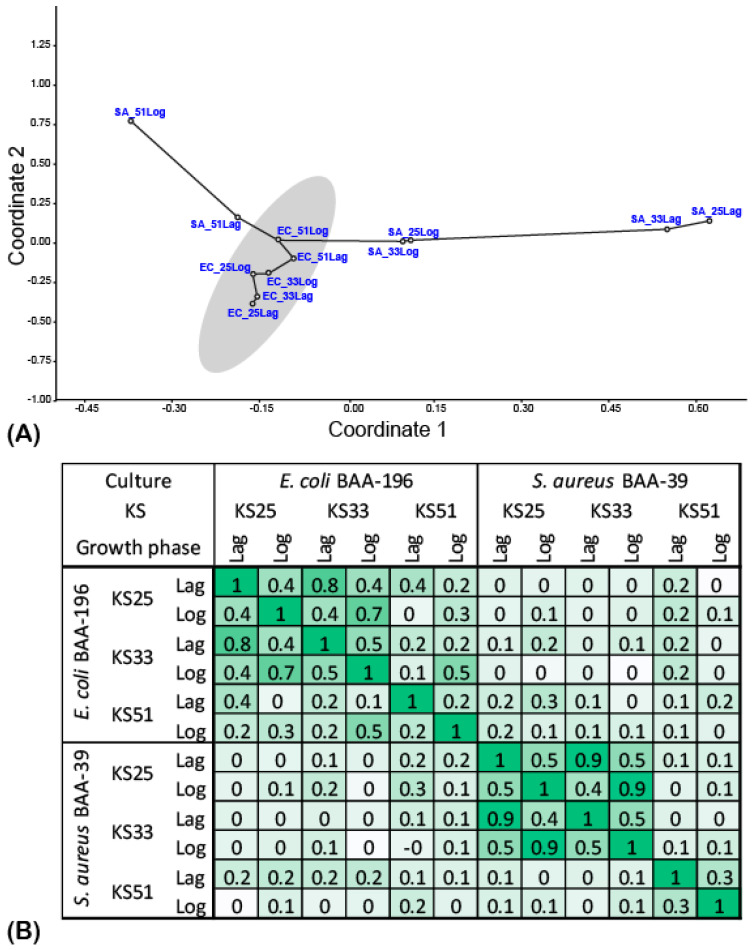
Co-regulation of gene expression in two tested bacterial cultures under the effect of the complexes of iodine with different amino acids applied at the different growth phases. (**A**) Principle component analysis (PCoA) plot of the experiments revealed by comparison of Log_2_(fold_change) values of 725 homologous genes shared by *E. coli* BAA-196 and *S. aureus* BAA-39. The names of the experiments were abbreviated as follows: EC_25Lag—*E. coli* BAA-196 culture treated with KS25 in the Lag growth phase; EC_25Log—*E. coli* BAA-196 culture treated with KS25 in the Log growth phase; EC_33Lag—*E. coli* BAA-196 culture treated with KS33 in the Lag growth phase; EC_33Log—*E. coli* BAA-196 culture treated with KS33 in the Log-growth phase; EC_51Lag—*E. coli* BAA-196 culture treated with KS51 in the Lag growth phase; EC_51Log—*E. coli* BAA-196 culture treated with KS51 in the Log growth phase; SA_25Lag—*S. aureus* BAA-39 culture treated with KS25 in the Lag growth phase; SA_25Log—*S. aureus* BAA-39 culture treated with KS25 in the Log growth phase; SA_33Lag—*S. aureus* BAA-39 culture treated with KS33 in the Lag growth phase; SA_33Log—*S. aureus* BAA-39 culture treated with KS33 in the Log growth phase; SA_51Lag—*S. aureus* BAA-39 culture treated with KS51 in the Lag growth phase; SA_51Log—*S. aureus* BAA-39 culture treated with KS51 in the Log growth phase. The 95% confidence deviation area of the principle coordinate values calculated for *E. coli* BAA-196 experiments is shaded. Graphs of the minimal spanning tree are shown. (**B**) Heatmap plot of aggregated Pearson correlation coefficients calculated for pairs of the conditions using the fold change values of differential regulation of expression of the homologous genes. Darker green shading depicts higher correlation.

Differential gene expression patterns of *E. coli* BAA-196 and *S. aureus* BAA-39 obtained after 5 min cultivation with the complexes KS25, KS33, and KS51 at the lagging (Lag) and the logarithmic (Log) growth phases were analyzed and compared using the principle component analysis (PCoA) based on the fold change matrices estimated for all differentially expressed genes showing |fold change| ≥ 2.0 and *p*-values ≤ 0.05 at least at one of the conditions. Down-regulation and up-regulation of the genes of *E. coli* BAA-196 and *S. aureus* BAA-39 at different conditions are summarized in Supplementary Table S2. The resulting PCoA plot is shown in Figure 3A and a heatmap presentation of the aggregated Pearson coefficients calculated for pairs of the conditions is shown in Figure 3B.

*E. coli* samples treated with the tested compounds at different growth phases showed a significant level of gene co-regulation (shaded area in Figure 3A and the relatively higher correlation coefficients calculated for this group of samples in Figure 3B. Compared to *E. coli*, the patterns of the regulated genes in *S. aureus* differed to a greater extent at the different growth phases and under the effects of the different complexes. It was, however, noticed that in both model organisms, the samples treated with KS25 and KS33 were grouped together; however, the gene regulation varied at the different growth phases. For example, the aggregated Pearson correlation calculated for gene regulation under the effects of KS25 and KS33 on *E. coli* at the Lag-phase (C_EC_KS25Lag|EC_KS33Lag_) was 0.79 and that at the Log-phase (C_EC_KS25Log|EC_KS33Log_) was 0.69; while the correlations C_EC_25Lag|EC_25Log_ and C_EC_33Lag|EC_33Log_ were 0.41 and 0.39, respectively. The patterns of gene regulation under the effect of KS51 on *E. coli* at both growth phases were clustered together by PCoA, and they differed from the gene regulation patterns under the effects of KS25 and KS33 at both growth phases.

The patterns of gene regulation in *S. aureus* at the same conditions were dissimilar to those in *E. coli* (Figure 3). Nevertheless, the effect of KS25 and KS33 on the gene expression in *S. aureus* at the same growth phase were more similar to each other (Pearson correlation was around 0.92) than the patterns obtained at different growth phases under the effects of the same complexes (Pearson correlation around 0.47), as it was also true for *E. coli*. The patterns of gene regulation in *S. aureus* treated with KS51 at the Lag and Log growth phases were dissimilar to all the other patterns, and they showed only a moderate similarity with one another: C_SA_51Lag|SA_51Log_ = 0.31.

Only one gene, the copper efflux pump *copA*, was generally up-regulated by all the iodine-containing complexes in both model microorganisms. This gene was strongly activated in *E. coli*; however, it was not activated in *S. aureus* when treated with KS51. Another gene with the general up-regulation pattern was NADPH/FMN-dependent nitroreductase *nfsA*. This enzyme plays a key role in redox homeostasis, oxidative stress responses, and reduction of chromate toxicity [27].

**Figure 4:**
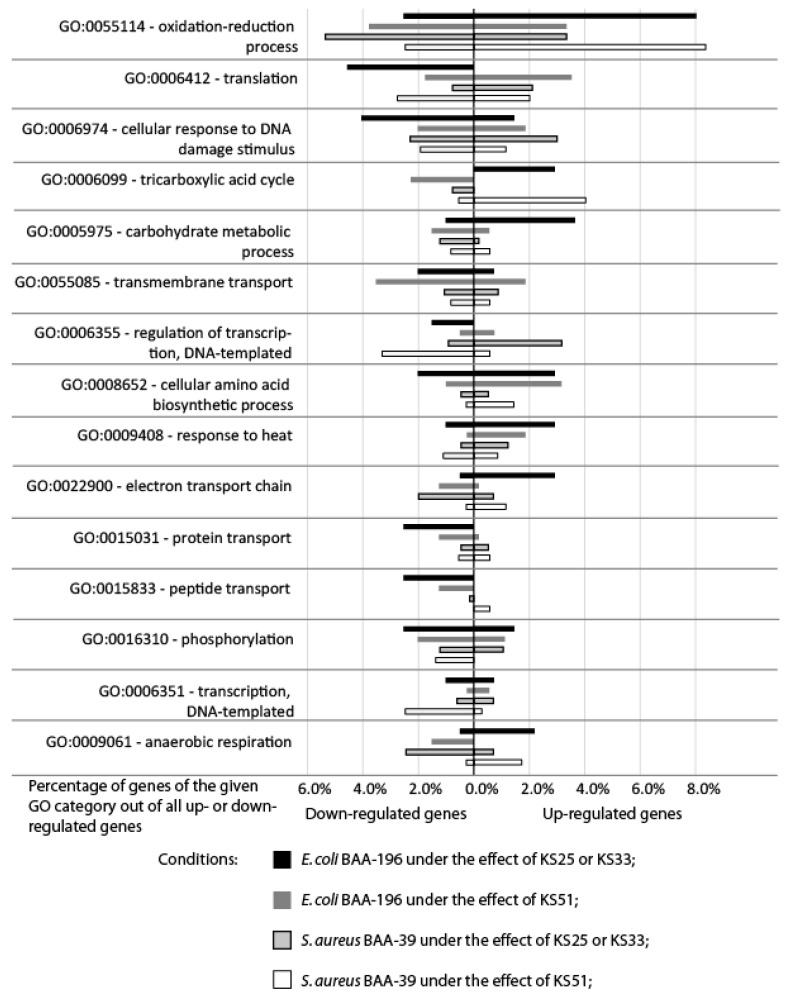
Percentages of up- and down-regulated genes associated with the different bioprocesses GO-terms (110 functional genes in total listed in Supplementary Table S2) in experiments with KS25, KS33, and KS51 in the Lag- and Log-growth phases.

Other co- and counter-regulated genes of the model microorganisms are shown in Supplementary Table S2. The distribution of the strongly regulated functional genes over GO bioprocess terms is shown in Figure 4. Due to a high level of similarity in gene regulation patterns caused by the actions of KS25 and KS33 on both bacteria (see Figure 3B), their effects were combined in this comparison in Figure 4. The genes most altered by the treatment with the iodine-containing complexes were involved in the oxidation–reduction processes. These genes were mostly up-regulated in *E. coli* treated with KS25 and 33, and in *S. aureus* treated with KS51. In other experiments, these genes were regulated differentially and predominantly inhibited in *E. coli* treated with KS51. Strong down-regulation of the genes involved in protein translation; cellular responses to DNA damage stimuli; and transmembrane transport, especially the transport of proteins and peptides, was characteristic of *E. coli* treated with KS25&33. In contrast, carbohydrate metabolic processes, the tricarboxylic acid cycle (TCA) cycle, amino acid biosynthesis, heat stress response, and anaerobic respiration proteins with the electron transport chain were generally up-regulated in *E. coli* treated with KS25&33. Unexpectedly, the patterns of gene regulation under the effect of KS25&33 in *E. coli* and under the effect of KS51 in *S. aureus* showed a higher similarity to each other than when the strains were treated with the same complexes (Figure 3). Many transcriptional regulators were down-regulated in *S. aureus* treated with KS51, which were not regulated by the effect of KS25 and KS33 on the same organism. Transmembrane transport was strongly inhibited in *S. aureus* under the effect of all the complexes and not so much in *E. coli*. In the experiments where *E. coli* was treated with KS51 and *S. aureus* was treated with KS25 and 33, the specific response to the treatment consisted of up-regulation of the genes involved in translation and cellular response to DNA damage, whereas the TCA and respiration genes, as well as the genes involved in carbohydrate metabolism and transmembrane transport, were down-regulated.

**Figure 5:**
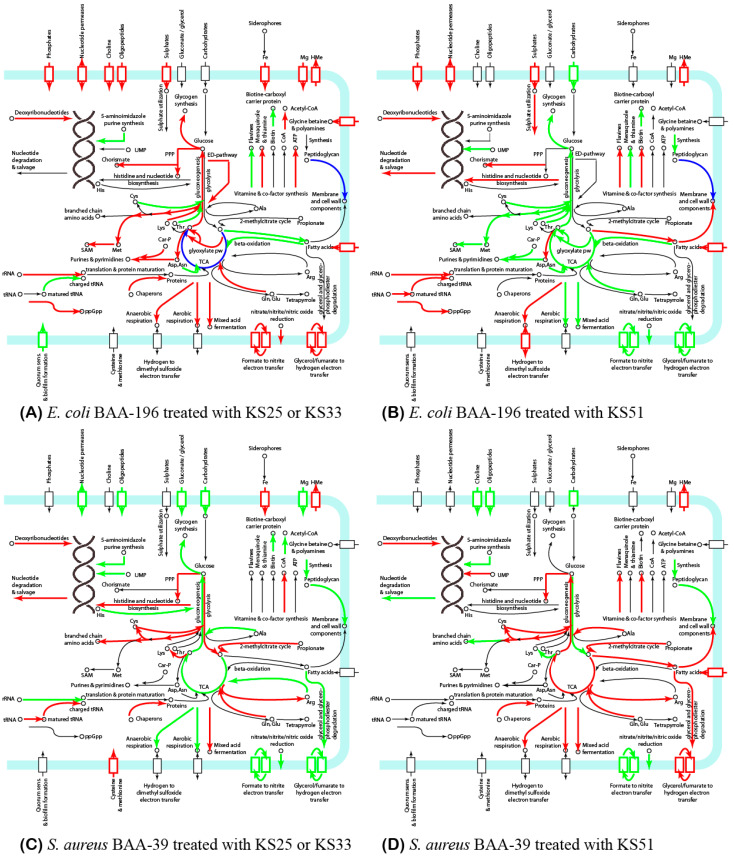
A summary of metabolic pathways, which possibly are affected by the treatment of the model microorganisms. (**A**) *E. coli* BAA-196 with KS25 and KS33; (**B**) *E. coli* BAA-196 with KS51; (**C**) *S. aureus* BAA-39 with KS25 and KS33; (**D**) *S. aureus* BAA-39 with KS25. Up- and down-regulation of the metabolic pathways are depicted, respectively, by the arrows of red and green colors. Pathways regulated differentially at the Lag- and Log-growth phases are depicted by blue arrows. Cell membrane and cell wall-associated proteins are shown by blocks depicted by the same color scheme. Individual pathways and key compounds are labeled.

Metabolic pathways affected by the treatment with the iodine-containing complexes were predicted using the PathwayTools v.26.0 software and summarized in Supplementary Table S3 and Figure 5. The central metabolism of the bacteria was affected in all the experiments. The TCA cycle pathway tended to be activated in *E. coli* treated with KS25 and in *S. aureus* treated with KS51, while the treatment of *E. coli* with KS33 and KS51 and the treatment of *S. aureus* with KS25 and 33 in the Lag-growth phase inhibited the TCA cycle. Catabolic glyoxylate shunt and the gluconeogenic pathway were activated in *E. coli* under the effect of KS25 and 33, whereas KS51 inhibited the glyoxylate pathway. Activation of the gluconeogenesis in *E. coli* treated with KS25 and 33 over the glycolysis is compensated by activation of the Entner–Doudoroff (ED) pathway bypassing the glycolysis. Genes of the non-oxidative branch of the pentose phosphate pathway (PPP) also were up-regulated at this condition.

In *S. aureus*, the switch between glycolysis and gluconeogenesis is controlled by two versions of glyceraldehyde-3-phosphate dehydrogenases, *gapA1* and *gapA2* [28]. The latter enzyme is homolog of *gapA* of *E. coli*; however, the version of this enzyme present in *S. aureus* participates exclusively in gluconeogenesis, whereas its homolog from *E. coli* is bidirectional. Treatment of *S. aureus* with KS25 and 33 activated *gapA1* and inhibited *gapA2*, which corresponds to an activation of glycolysis. By way of contrast, treatment of this culture with IPS51 in the Log-growth phase inhibits *gapA1*, which was in favor of gluconeogenesis.

Genes involved in aerobic respiration and the fermentation pathways were activated in *E. coli* treated with KS25 and 33. Many of these genes were inhibited in *E. coli* under the effect of KS51, except for several genes involved in dissimilatory nitrate reduction (*narL*, *narK* and *narG*) and also *ndh* respiratory NADH dehydrogenase. Treatment of *S. aureus* with KS25 and 33 activated the acetate/glycerate fermentation, while other pathways of the aerobic and anaerobic respiration were generally inhibited. Treatment of *S. aureus* with KS51-activated fumarate reductase genes *frdAB* is involved in the anaerobic respiration but inhibits the glycerate metabolism. The nitrate-based respiration was generally inhibited in *S. aureus* in all conditions.

Synthesis of amino acids was activated in all the experiments except for *E. coli* treated with KS51, where these biosynthetic pathways were strongly inhibited. Cysteine synthesis and metabolism were activated in *S. aureus*, but not in *E. coli*. Contrarily, methionine metabolism and sulfate uptake were activated in *E. coli*.

Fatty acid synthesis was activated in *E. coli* and *S. aureus* under the effect of KS51 but not KS25 and 33; however, fatty acid uptake and transmembrane transportation were activated at all these conditions except for *S. aureus* treated with KS25 and 33. The pathway of beta-oxidation of fatty acids was down-regulated in *E. coli* under the effect of all the compounds, but it was not regulated in *S. aureus*. Synthesis of peptidoglycan was generally inhibited in *S. aureus* treated with all the complexes. In *E. coli*, the synthesis of peptidoglycan was activated by KS51 in the Lag-growth phase, but it was inhibited in the Log-growth phase. Treatment with KS25 and 33 did not affect the peptidoglycan synthesis in this bacterium; however, various genes involved in peptidoglycan processing were differentially regulated by all the complexes.

## 4. Discussion

In this study, we analyzed differential gene expression in *E. coli* ATCC BAA-196 and *S. aureus* ATCC BAA-39 treated with the complexes KS25, KS33, and KS51 containing respectively glycine, L-alanine, and L-isoleucine loaded with iodine. Different responses were observed in the two model microorganisms representing the Gram-positive and Gram-negative pathogens (Figure 5). It was found that the effect of iodine-containing complexes on bacterial metabolism varied significantly depending on the moiety of organic molecules of the complexes. It may be explained by a different distribution of iodine in the forms of iodide ion, molecular iodine, and triiodide in these complexes, which are characterized by different mobility and penetrability through the cellular barriers, and also by the properties of the involved amino acids. The gene expression patterns were also affected by the growth phases of the bacterial cultures when the complexes were applied. The dissimilarity of responses of the different tested bacterial pathogens at different growth phases was reported in a previous study with the therapeutic complex FS-1 [9]. It should be noted that Figure 5 presents a general overview of the affected metabolic pathways, which have to be verified in future studies. The main conclusion from this finding is that the effect of iodine-containing complexes on Gram-positive and Gram-negative pathogens is dissimilar; however, several important commonalities were found.

Bacteria can alter their own metabolism in response to environmental challenges. The cells utilize transcription regulators to control genes and operons belonging to various metabolic pathways or stress response mechanisms [29,30]. The most common effect of the iodine-containing complexes used in this study was a strong up-regulation of the efflux pumps and detoxication enzymes such as copper transporter *copA*, multicopper oxidase *cueO* in *E. coli*; zinc transporter *zitB*, heavy metal ion efflux pump *zntA*, and mercuric ion reductase *merA2* in *S. aureus*; and *nfsA* reductase in both microorganisms. The genes *copA*, *cueO*, and *zitB* were significantly up-regulated when the same model cultures were treated with the drug FS-1 [8,9]. Many other genes associated with the oxidation–reduction processes were also regulated (Figure 4). Among them, the genes involved in preventing oxidative stress damages caused by reactive oxygen species (ROS), which include protein–methionine–sulfoxide reductase catalytic subunit *yedY*; superoxide dismutase *sodA*; alkyl hydroperoxide reductase *ahpF*; glutaredoxin-2 *grxB*; and the oxidative stress-related phosphoglyceromutase *gpmI* were activated in all experiments with *S. aureus*. In the *E. coli*, treatment with the iodine-containing complexes activated quercetin 2,3-dioxynase *yhhW*. This gene encodes a bacterial pirin, which is most likely involved in gene regulation, DNA replication control, and response to oxidative stress [31]. All these facts indicate that the prime target of iodine released by the complexes was the bacterial cell wall, which degraded due to the oxidizing power of iodine and led to an increased penetrability of the affected cells for metal ions and other toxic compounds, including antibiotics. This finding is in agreement with the previous reports where an increased concentration of ROS in the cytoplasm of the microorganisms treated with FS-1 [8,9,10]. Abiotic stresses induce an increase in the level of ROS in the cellular cytoplasm resulting in the production of toxic methylglyoxal [32] and damaging intracellular lipids, proteins, and nucleic acids [33].

This scheme of action of the iodine-containing complexes on bacterial cells explains other observed regulations. In order to reduce the intracellular influx of iodine bound to amino acids, the treated cultures down-regulate the activities of the uptake transporters and permeases of organic nitrogen-rich compounds. It appears that *E. coli* coped better with the prevention of the influx of iodine into cytoplasm than *S. aureus*, agreeing with the previous study when the drug FS-1 was used [9]. A deleterious effect of intracellular iodine caused the activation of synthesis of various chaperons and shock proteins in *S. aureus* that was not observed in the treated *E. coli*.

The effects of KS25 and KS33 were similar, whereas the effect of KS51 on the gene expression in the treated bacterial cultures was more culture-dependent (Figure 1). Iodide ions, molecular I_2_, and triiodide anions existed in equilibrium in the aqueous solutions, and a shift towards one or another could be moderated by the presence of active electron donors or acceptors in the solution [34]. The difference in the gene expression may be due to different proportions of the molecular iodine, triiodide, and iodide ions in the complexes with different amino acids in their composition. The treatment of the two model microorganisms representing Gram-positive and Gram-negative pathogens caused rather dissimilar regulation of the central metabolism, including the glycolytic pathways, TCA, carbohydrate, and amino acid metabolism, aerobic and anaerobic respiration. These differences may be explained by the different structural organization of the cell walls of the Gram-positive and Gram-negative bacteria and by the general differences in the metabolism of *E. coli* and *S. aureus*, such as the availability of the additional ED and glyoxylate pathways, which are absent in *S. aureus*. Alternative cell wall organization may also be attributed to different growth phases that may explain differences in gene regulation patterns when the complexes were applied at the end of the lagging and in the mid-logarithmic growth phases. A similar difference in the reactions of the model microorganisms at different growth phases to the effect of the drug FS-1 was reported in the previous study [9].

## 5. Conclusions

In this study, we demonstrated differential gene expression in *E. coli* ATCC BAA-196 and *S. aureus* ATCC BAA-39 treated with the iodine-containing complexes KS25, KS33, and KS51 in comparison with the untreated cultures served as the negative controls. A common response of these two model microorganisms to the treatment was a strong activation of heavy metal efflux pumps that indicate possible degradation of the cell wall barriers and the increased permeability of the cellular membrane. The genes involved in oxidation-reduction balancing were also strongly affected in both bacteria that suggested oxidative stress.

The striking discovery was that the creation of iodine-containing complexes with different amino acids moderates the specific activities of these complexes. It was demonstrated in this study by the differential gene regulation caused by the complexes of iodine with glycine and L-alanine (KS25 and KS33) in contrast to the effect caused by the complex with L-isoleucine (KS51). The complexes KS33 and KS51 were synthesized by applying LiI to amino acids followed by loading equimolar concentrations of molecular iodine (Table 1) in contrast to the complex KS25, which did not contain metal cations in its structure (Figure 1A,B). UV-spectra of water solutions of KS33 and KS51 were more similar to each other and differed from that of KS25 (Figure 2). However, the chemical similarity between the amino acids used in the complexes (small ambivalent glycine and alanine in KS25 and KS33 versus large hydrophobic isoleucine in KS33) had a greater effect on the similarity of the gene regulation patterns (Figure 3).

The aim of the project was to verify the hypothesis that the compounds of the organic moiety of iodine-containing complexes can moderate the activity of iodine and its effect on pathogens. The transcriptomics analysis of the effects of these three complexes created a ground for further studies by identifying the biological systems and individual genes affected similarly or dissimilarly by the complexes. In particular, it can be concluded from this study and from the previous results with the drug FS-1 [8,9] that up-regulation of the genes *copA*, *cueO*, and *zitB* may serve as a marker of the level of degradation of the cell wall of the pathogens by iodine-containing complexes. It should be noted that the designed complexes were not planned to be used as future antimicrobial drugs. The results obtained in this study will facilitate the design of future iodine-containing therapeutic complexes by guiding the process of optimization of their organic composition.

## Figures and Tables

**Table 1:** Complexes of iodine with amino acids synthesized for this study.

Complex	Source of Iodine	Amino Acid
KS25	I_2_	Alanine
KS33	I_2_ + LiI	Glycine
KS51	I_2_ + LiI	Isoleucine

**Table 2:** Minimal bactericidal concentration (MBC) of the complexes determined for the test cultures used in this study.

Test-Cultures	MBC Values (mg/mL), I_2_ Content Concentration per Total Concentration of the Substance		
	KS25	KS33	KS51
*E. coli* BAA-196	0.50/4.0	0.32/2.0	0.57/2.0
*S. aureus* BAA-39	0.50/4.0	0.32/2.0	0.57/2.0

**Table 3:** Repetitions of the experiments on RNA extraction and sequencing.

Strain	Growth Phase	Treatment with the Complexes			Negative Control (NC)
		KS25	KS33	KS51	
*E. coli* BAA-196	Lag	3	3	3	6
	Log	3	3	3	6
*S. aureus* BAA-39	Lag	3	3	3	6
	Log	6	6	3	9

## Data Availability

The RNA reads generated for this study are available from the NCBI SRA database through the BioProjects PRJNA557356 and PRJNA480363 created, respectively, for the genomes of *E. coli* ATCC BAA-196 and *S. aureus* ATCC BAA-39. The links to the respective SRA Experiments are available on the BioProject Web-pages. The predicted X-ray structures of the complexes KS25, KS33, and KS51 were deposited at the CCDC database (https://www.ccdc.cam.ac.uk/, accessed on 26 June 2023) under accession numbers 1036607, 1036667, and 1436137, respectively.

## Supplementary Materials

The following supporting information can be downloaded at: https://www.mdpi.com/article/10.3390/microorganisms11071705/s1 (https://zenodo.org/record/7556403#.ZBMFRZdByHs, accessed on 26 June 2023): Supplementary Table S1. X-ray diffraction and refinement parameters; Supplementary Table S2. Genes regulated with statistical reliability (|fold change| ≥ 2.0; *p*-value ≤ 0.05) under the effect of iodine-containing complexes at least at one condition; Supplementary Table S3. Regulation of expression of genes involved in the central metabolism of bacteria by the three tested complexes.
