# Supplementary material for: The Effect of Three Complexes of Iodine with Amino Acids on Gene Expression of Model Antibiotic Resistant Microorganisms Escherichia coli ATCC BAA-196 and Staphylococcus aureus ATCC BAA-39
Source: Microorganisms. 2023 Jun 29;11(7):1705. doi: 10.3390/microorganisms11071705 (PMC10383335; doi:10.3390/microorganisms11071705)
Supplement: Supplementary file 1 [file microorganisms-11-01705-s001.zip › Table S1.pdf]

Table S1. X-ray diffraction and refinement parameters

Crystal Data			
**Compound**	**KS25**	**KS33**	**KS51**
Formula	$2C_3H_7NO_2 \cdot HI_3$	$3C_2H_5NO_2 \cdot LiI$	$4C_6H_{13}NO_2 \cdot 2HI \cdot H_2O$
Molecular Weight	559.91	359.05	798.54
Crystal System; Space group	Monoclinic, $P2_1/c$	Monoclinic, $P2_1$	Monoclinic, C2
Lattice constants: a, b, c [Å]	8.1057(16); 8.9853(18); 10.541(2)	5.4692(11); 9.898(2); 11.193(2)	22.8411(7); 5.8290(2); 16.2238(5)
alpha, beta, gamma [deg]	90; 100.29(3); 90	90; 90,67(3); 90	90; 123.658(2);90
V, $[Å]^3$; Z	755.4(3); 2	605.9(2); 2	1797.94(12); 2
D(calc)[g/cm^3]; F(000)	2.462; 512	1.968; 352	1.475; 812
Mu(MoKα) [mm^{-1}]	6.205	2.662	1.796
Crystal Size [mm]	0.10 x 0.10 x 0.20	0.14 x 0.17 x 0.20	0.02 x 0.10 x 0.20
**Data Collection**			
T [^{0}K]; Radiation [Å]	200; MoKα; λ=0,71073	200; MoKα; λ=0,71073	100; MoKα; λ=0.71073
θ_{min};$\theta_{max,}$ [deg]	2.6; 30.0	1.8; 30.0	3.2; 29.7
Dataset	0:11; 0:12; -14:14	0:7 ; -13:13 ; -15:15	-31:31; -8:8 ; -22:22
Tot., Uniq. Data, R(int)	2338, 2195, 0.023	4107, 3515, 0.022	9148, 4462, 0.029
Observed data, I > 2,0 sigma(I)	1737	3322	3726
**Refinement**			
Nref, Npa	2195; 102	3515; 157	4462; 185
R, wR^2, S	0.0351; 0.0859; 1.10	0.0377; 0.0955; 1.25	0.0458; 0.0684; 1.05
	w = 1/[\s^2^(Fo^2^)+(0.0374P)^2^+0.2440P], where P=(Fo^2^+2Fc^2^)/3	w = 1/[\s^2^(Fo^2^)+(0.0374P)^2^+0.2440P], where	w = 1/[\s^2^(Fo^2^)+(0.0374P)^2^+0.2440P], где P=(Fo^2^+2Fc^2^)/3

		P=(Fo^2^+2Fc^2^)/3	
Max. and Av. Shift/Error	0.00; 0.00	0.00; 0.00	0.00; 0.00
Min. and Max. Resd. Dens. [e/Å^3]	-1.03; 1.45	-1.56; 2.08	-0.94; 0.88
