# Supplementary material for: The Effect of Three Complexes of Iodine with Amino Acids on Gene Expression of Model Antibiotic Resistant Microorganisms Escherichia coli ATCC BAA-196 and Staphylococcus aureus ATCC BAA-39
Source: Microorganisms. 2023 Jun 29;11(7):1705. doi: 10.3390/microorganisms11071705 (PMC10383335; doi:10.3390/microorganisms11071705)
Supplement: Supplementary file 1 [file microorganisms-11-01705-s001.zip › Table S2.pdf]

Table S2. Genes regulated with a statistical reliability (|*fold change* | ≥ 2.0; *p* -value ≤ 0.05) under the effect of iodine-containing complexes at least at one condition.

1 - upregulation; -1 - downregulation;

**Locus tag in *E. coli* BAA-196**	**Locus tag in *S. aureus* BAA-39**	**Gene**	***E.coli* BAA-196**						***S.aureus* BAA-39**						**Annotation**
			IPC25		IPC33		IPC51		IPC25		IPC33		IPC51		
			Lag	Log	Lag	Log	Lag	Log	Lag	Log	Lag	Log	Lag	Log	
BAA196NC_0001	HMPRNC0000_0001	*dnaA*							-1						Chromosomal replication initiation protein
BAA196NC_0054	HMPRNC0000_1766	*spoT*			1		1								Bifunctional (p)ppGpp synthetase II/ guanosine-3',5'-bis pyrophosphate 3'-pyrophosphohydrolase
BAA196NC_0056	HMPRNC0000_1263	*gmk*					1								Guanylate kinase
BAA196NC_0065	HMPRNC0000_1265	*dfp*						1			1				Bifunctional phosphopantothenoylcysteine decarboxylase/phosphopantothenate synthase
BAA196NC_0066	HMPRNC0000_1785	*yicR*				-1									DNA repair protein RadC
BAA196NC_0089	HMPRNC0000_0594	*kbl*							-1						2-amino-3-ketobutyrate coenzyme A ligase
BAA196NC_0094	HMPRNC0000_0864	*gpmI*							1	1	1	1			Phosphoglyceromutase
BAA196NC_0098	HMPRNC0000_1589	*gpsA*	-1				-1	-1		1		1			NAD(P)H-dependent glycerol-3-phosphate dehydrogenase
BAA196NC_0120	HMPRNC0000_0169	*aldB*					-1	-1		1		1			Aldehyde dehydrogenase B
BAA196NC_0145	HMPRNC0000_2772	*xylB*											-1		Xylulokinase
BAA196NC_0158	HMPRNC0000_0955	*tiaE*							-1						2-ketoaldonate reductase/glyoxylate reductase B
BAA196NC_0170	HMPRNC0000_1025	*dppD*						-1							Dipeptide transporter
BAA196NC_0249	HMPRNC0000_0035	*zntA*		1		1			1	1	1	1			Zinc, cobalt and lead efflux system
BAA196NC_0271	HMPRNC0000_0224	*ggt*		1			-1		1		1				Gamma-glutamyltranspeptidase periplasmic precursor
BAA196NC_0316	HMPRNC0000_1928	*pck*		1			-1		-1		-1				Phosphoenolpyruvate carboxykinase
BAA196NC_0318	HMPRNC0000_0538	*hslO*							1	1	1	1			Hsp33-like chaperonin
BAA196NC_0353	HMPRNC0000_2659	*nirB*						1	-1		-1				Nitrite reductase, large subunit, NAD(P)H-binding
BAA196NC_0359	HMPRNC0000_0787	*pabA*				1									Para-aminobenzoate synthase component II
BAA196NC_0360	HMPRNC0000_0987	*argD*							-1		-1				Bifunctional acetylornithine aminotransferase/ succinyldiaminopimelate aminotransferase
BAA196NC_0378	HMPRNC0000_0588	*rpsL*								1		1			30S ribosomal protein S12
BAA196NC_0379	HMPRNC0000_0589	*rpsG*								1		1			30S ribosomal protein S7
BAA196NC_0380	HMPRNC0000_0590	*fusA*		1						1		1			Elongation factor EF-2
BAA196NC_0400	HMPRNC0000_2462	*rplC*		1											50S ribosomal protein L3
BAA196NC_0402	HMPRNC0000_2460	*rplW*					-1		1	1		1			50S ribosomal protein L23
BAA196NC_0404	HMPRNC0000_2458	*rpsS*					-1								30S ribosomal protein S19
BAA196NC_0405	HMPRNC0000_2457	*rplV*												-1	50S ribosomal protein L22
BAA196NC_0407	HMPRNC0000_2455	*rplP*		1						1		1			50S ribosomal protein L16
BAA196NC_0408	HMPRNC0000_2454	*rpmC*				1				1	-1	1			50S ribosomal protein L29
BAA196NC_0409	HMPRNC0000_2453	*rpsQ*								1		1			30S ribosomal protein S17
BAA196NC_0411	HMPRNC0000_2451	*rplX*						-1						-1	50S ribosomal protein L24
BAA196NC_0412	HMPRNC0000_2450	*rplE*								1		1		-1	50S ribosomal protein L5
BAA196NC_0414	HMPRNC0000_2449	*rpsH*		1										-1	30S ribosomal protein S8
BAA196NC_0415	HMPRNC0000_2448	*rplF*						-1		1		1		-1	50S ribosomal protein L6
BAA196NC_0416	HMPRNC0000_2447	*rplR*		1						1		1		-1	50S ribosomal protein L18
BAA196NC_0418	HMPRNC0000_2445	*rpmD*		1		1				1		1		-1	50S ribosomal protein L30
BAA196NC_0419	HMPRNC0000_2444	*rplO*		1										-1	50S ribosomal protein L15
BAA196NC_0420	HMPRNC0000_2443	*secY*								1		1			Protein translocase subunit SecY
BAA196NC_0424	HMPRNC0000_1859	*rpsD*							-1		-1				30S ribosomal protein S4
BAA196NC_0425	HMPRNC0000_2437	*rpoA*						-1						-1	DNA-directed RNA polymerase subunit alpha
BAA196NC_0428	HMPRNC0000_0064	*zntR*							1						Zinc-responsive transcriptional regulator
BAA196NC_0430	HMPRNC0000_1447	*mscL*						1							Large-conductance mechanosensitive channel
BAA196NC_0432	HMPRNC0000_1274	*rsmB*								1		1			16S rRNA m5C967 methyltransferase, S-adenosyl-L-methionine-dependent
BAA196NC_0458	HMPRNC0000_1651	*accC*				-1		-1	-1						Acetyl-CoA carboxylase
BAA196NC_0459	HMPRNC0000_1652	*accB*					1								Acetyl-CoA carboxylase
BAA196NC_0461	HMPRNC0000_2623	*yhdH*		1											Predicted oxidoreductase, Zn-dependent and NAD(P)-binding
BAA196NC_0477	HMPRNC0000_2889	*mdh*		1									1		Malate dehydrogenase
BAA196NC_0482	HMPRNC0000_2432	*rplM*			-1		1								50S ribosomal protein L13
BAA196NC_0498	HMPRNC0000_0488	*gltD*		1					1	1	1	1			Glutamate synthase, 4Fe-4S protein, small subunit
BAA196NC_0499	HMPRNC0000_0487	*gltB*		1		1			1	1	1	1			Glutamate synthase, large subunit
BAA196NC_0507	HMPRNC0000_0852	*yhbJ*		-1					1	1	1	1			Hypothetical protein
BAA196NC_0525	HMPRNC0000_1584	*ispB*							-1				-1		Octaprenyl diphosphate synthase
BAA196NC_0526	HMPRNC0000_1780	*rplU*					1								50S ribosomal protein L21
BAA196NC_0527	HMPRNC0000_1778	*rpmA*					1	1							50S ribosomal protein L27
BAA196NC_0533	HMPRNC0000_1728	*yhbY*		-1				-1							Predicted RNA-binding protein
BAA196NC_0535	HMPRNC0000_0537	*ftsH*								1		1			Protease, ATP-dependent zinc-metallo
BAA196NC_0546	HMPRNC0000_1339	*rpsO*		-1							-1				30S ribosomal protein S15
BAA196NC_0548	HMPRNC0000_1340	*pnp*												-1	Polynucleotide phosphorylase/polyadenylase
BAA196NC_0552	HMPRNC0000_1755	*yhbW*							1	1	1	1			Luciferase-like monooxygenase
BAA196NC_0594	HMPRNC0000_1548	*tdcB*	-1	-1		-1	-1	-1							Threonine dehydratase
BAA196NC_0606	HMPRNC0000_2583	*yhaH*						1							Predicted inner membrane protein
BAA196NC_0646	HMPRNC0000_2227	*ygjD*							1						O-sialoglycoprotein endopeptidase
BAA196NC_0706	HMPRNC0000_2568	*yghA*				1				-1		-1			Oxidoreductase
BAA196NC_0737	HMPRNC0000_2616	*lctP*												-1	L-lactate permease
BAA196NC_0749	HMPRNC0000_2047	*mutY*			-1										Adenine DNA glycosylase
BAA196NC_0764	HMPRNC0000_1706	*yggJ*							1	1		1			Hypothetical protein
BAA196NC_0768	HMPRNC0000_1927	*metK*		1				1							S-adenosylmethionine synthetase
BAA196NC_0782	HMPRNC0000_0861	*pgk*							1	1		1			Phosphoglycerate kinase
BAA196NC_0785	HMPRNC0000_0893	*argO*									1				Arginine exporter protein

BAA196NC_0804	HMPRNC0000_1665	*gcvT*							-1		-1				Glycine cleavage system aminomethyltransferase T
BAA196NC_0805	HMPRNC0000_0904	*gcvH*		1		1		1			-1				Glycine cleavage system protein H
BAA196NC_0810	HMPRNC0000_2386	*yqfA*						-1					-1		Predicted oxidoreductase, inner membrane subunit
BAA196NC_0818	HMPRNC0000_0838	*prfB*							1		1				Peptide chain release factor 2
BAA196NC_0883	HMPRNC0000_1536	*thyA*							-1						Thymidylate synthase
BAA196NC_0921	HMPRNC0000_2778	*gudP*							-1						Predicted D-glucarate transporter
BAA196NC_0930	HMPRNC0000_2324	*pyrG*								1		1			CTP synthetase
BAA196NC_0946	HMPRNC0000_2912	*cysJ*							1						Sulfite reductase, alpha subunit, flavoprotein
BAA196NC_0976	HMPRNC0000_0769	*ygbI*							1	1	1	1			Predicted DNA-binding transcriptional regulator
BAA196NC_1014	HMPRNC0000_1351	*recA*											-1		Recombinase A
BAA196NC_1016	HMPRNC0000_1749	*alaS*				1									Alanyl-tRNA synthetase
BAA196NC_1034	HMPRNC0000_0809	*nrdF*											-1		Ribonucleotide-diphosphate reductase beta subunit
BAA196NC_1048	HMPRNC0000_2892	*gabT*							1		1				4-aminobutyrate aminotransferase
BAA196NC_1090	HMPRNC0000_1711	*grpE*						1	1	1	1	1			Heat shock protein
BAA196NC_1093	HMPRNC0000_1301	*ffh*								1		1			Signal Recognition Particle (SRP) component with 4.5S RNA (ffs)
BAA196NC_1096	HMPRNC0000_1306	*trmD*					1								tRNA (guanine-N(1)-)-methyltransferase
BAA196NC_1107	HMPRNC0000_1249	*rluD*								1		1			23S rRNA pseudouridine synthase
BAA196NC_1108	HMPRNC0000_1237	*yfiH*							1						Hypothetical protein
BAA196NC_1109	HMPRNC0000_1008	*clpB*			1				1	1	1	1		1	Protein disaggregation chaperone
BAA196NC_1165	HMPRNC0000_1148	*suhB*					1								Inositol monophosphatase
BAA196NC_1169	HMPRNC0000_0939	*iscU*			1										Scaffold protein
BAA196NC_1181	HMPRNC0000_1583	*ndk*					1		-1		-1				Nucleoside diphosphate kinase
BAA196NC_1185	HMPRNC0000_1762	*hisS*		-1					-1						Histidyl-tRNA synthetase
BAA196NC_1191	HMPRNC0000_0434	*guaB*	1		1										Inositol-5-monophosphate dehydrogenase
BAA196NC_1192	HMPRNC0000_0435	*guaA*	1		1										Bifunctional GMP synthase/glutamine amidotransferase protein
BAA196NC_1200	HMPRNC0000_1110	*purN*			1						-1				Phosphoribosylglycinamide formyltransferase
BAA196NC_1201	HMPRNC0000_1109	*purM*	1		1									-1	Phosphoribosylaminoimidazole synthetase
BAA196NC_1202	HMPRNC0000_2304	*upp*								-1		-1			Uracil phosphoribosyltransferase
BAA196NC_1203	HMPRNC0000_1252	*uraA*			1				-1		-1				Uracil transporter
BAA196NC_1220	HMPRNC0000_2041	*bcp*					1								Thioredoxin-dependent thiol peroxidase
BAA196NC_1222	HMPRNC0000_1502	*dapA*											-1		Dihydrodipicolinate synthase
BAA196NC_1224	HMPRNC0000_1101	*purC*	1		1				-1						Phosphoribosylaminoimidazole-succinocarboxamide synthase
BAA196NC_1230	HMPRNC0000_1030	*yffB*								-1		-1			Hypothetical protein
BAA196NC_1231	HMPRNC0000_2477	*acrD*						-1							Aminoglycoside/multidrug efflux system
BAA196NC_1236	HMPRNC0000_1439	*tktB*		1											Transketolase 2, thiamin-binding
BAA196NC_1272	HMPRNC0000_0204	*murP*									-1				N-acetylmuramic acid phosphotransfer permease
BAA196NC_1273	HMPRNC0000_0203	*yfeU*									-1				N-acetylmuramic acid-6-phosphate etherase
BAA196NC_1289	HMPRNC0000_2088	*ligA*								1		1			NAD-dependent DNA ligase LigA
BAA196NC_1298	HMPRNC0000_0571	*gltX*								1		1			Glutamyl-tRNA synthetase
BAA196NC_1303	HMPRNC0000_0341	*nupC*		-1											Nucleoside (except guanosine) transporter
BAA196NC_1304	HMPRNC0000_1146	*mntH*									1				Manganese transport protein MntH
BAA196NC_1377	HMPRNC0000_1501	*usg*											-1		Putative beta-semialdehyde dehydrogenase
BAA196NC_1378	HMPRNC0000_2433	*truA*									1				tRNA pseudouridine synthase A
BAA196NC_1380	HMPRNC0000_1833	*accD*					1								Acetyl-CoA carboxylase subunit beta
BAA196NC_1381	HMPRNC0000_1787	*folC*							1	1	1	1			Bifunctional folylpolyglutamate synthase/ dihydrofolate synthase
BAA196NC_1384	HMPRNC0000_1106	*purF*	1		1						-1			-1	Amidophosphoribosyltransferase
BAA196NC_1392	HMPRNC0000_0857	*yfcH*				1									Conserved protein with NAD(P)-binding Rossmann-fold domain
BAA196NC_1393	HMPRNC0000_0541	*folX*						1							D-erythro-7,8-dihydroneopterin triphosphate 2'-epimerase and dihydroneopterin aldolase
BAA196NC_1400	HMPRNC0000_1846	*ackA*								1		1			Acetate kinase
BAA196NC_1433	HMPRNC0000_1074	*menD*						-1		1		1			2-succinyl-6-hydroxy-2, 4-cyclohexadiene-1-carboxylate synthase
BAA196NC_1459	HMPRNC0000_0989	*glpQ*									-1				Periplasmic glycerophosphodiester phosphodiesterase
BAA196NC_1467	HMPRNC0000_1455	*gyrA*								1		1			DNA gyrase subunit A
BAA196NC_1474	HMPRNC0000_0394	*atoB*									-1				Acetyl-CoA acetyltransferase
BAA196NC_1600	HMPRNC0000_2283	*thiM*							-1		-1				Hydroxyethylthiazole kinase
BAA196NC_1601	HMPRNC0000_2284	*thiD*							-1		-1				Phosphomethylpyrimidine kinase
BAA196NC_1608	HMPRNC0000_2322	*gatY*	-1				-1			-1		-1		1	D-tagatose 1,6-bisphosphate aldolase 2, catalytic subunit
BAA196NC_1615	HMPRNC0000_0269	*gatD*									1				Galactitol-1-phosphate dehydrogenase, Zn-dependent and NAD(P)-binding
BAA196NC_1625	HMPRNC0000_1744	*yegQ*							1	1		1			Predicted peptidase
BAA196NC_1672	HMPRNC0000_0128	*rfbX*		-1											Predicted polisoprenol-linked O-antigen transporter
BAA196NC_1683	HMPRNC0000_2982	*hisI*							1		1				Bifunctional phosphoribosyl-AMP cyclohydrolase/phosphoribosyl-ATP pyrophosphatase protein
BAA196NC_1684	HMPRNC0000_2983	*hisF*							1						Imidazole glycerol phosphate synthase subunit HisF
BAA196NC_1685	HMPRNC0000_2984	*hisA*							1		1				1-(5-phosphoribosyl)-5-[(5- phosphoribosylamino)methylideneamino] imidazole-4-carboxamide isomerase
BAA196NC_1686	HMPRNC0000_2985	*hisH*							1		1				Imidazole glycerol phosphate synthase subunit HisH
BAA196NC_1688	HMPRNC0000_0800	*hisC*							1		1				Histidinol-phosphate aminotransferase
BAA196NC_1689	HMPRNC0000_2989	*hisD*									1				Histidinol dehydrogenase
BAA196NC_1696	HMPRNC0000_2220	*yeeE*							1		1				Predicted inner membrane protein
BAA196NC_1697	HMPRNC0000_2219	*yeeD*							1		1				Hypothetical protein
BAA196NC_1737	HMPRNC0000_0595	*hchA*						1	1						Chaperone protein HchA
BAA196NC_1780	HMPRNC0000_2676	*fliY*							1	1	1	1			Cystine transporter subunit
BAA196NC_1782	HMPRNC0000_2675	*yecS*							1	1	1	1			Predicted transporter subunit: membrane component of ABC superfamily
BAA196NC_1783	HMPRNC0000_2003	*yecC*							1		1				Predicted transporter subunit: ATP-binding component of ABC superfamily
BAA196NC_1787	HMPRNC0000_1194	*uvrC*					-1		1						Excinuclease ABC subunit C
BAA196NC_1792	HMPRNC0000_2078	*ftnA*								-1		-1			Ferritin-like protein 2

BAA196NC_1819	HMPRNC0000_0666	*argS*	1				1								Arginyl-tRNA synthetase
BAA196NC_1829	HMPRNC0000_1761	*aspS*							-1						Aspartyl-tRNA synthetase
BAA196NC_1836	HMPRNC0000_0691	*znuB*							-1		-1				High-affinity zinc transporter membrane component
BAA196NC_1844	HMPRNC0000_1624	*zwf*									1			1	Glucose-6-phosphate 1-dehydrogenase
BAA196NC_1866	HMPRNC0000_1857	*yebR*					1								Hypothetical protein
BAA196NC_1920	HMPRNC0000_1815	*gapA*							-1		-1				Glyceraldehyde-3-phosphate dehydrogenase
BAA196NC_1928	HMPRNC0000_0658	*ydjG*											1		Predicted oxidoreductase
BAA196NC_1936	HMPRNC0000_2467	*topB*						-1							DNA topoisomerase III
BAA196NC_1938	HMPRNC0000_0988	*gdhA*							-1		-1				Glutamate dehydrogenase
BAA196NC_1949	HMPRNC0000_0994	*ydjX*											-1		Predicted inner membrane protein
BAA196NC_1963	HMPRNC0000_2410	*chbC*		-1					-1		-1		-1		N,N'-diacetylchitobiose-specific enzyme IIC component of PTS
BAA196NC_1977	HMPRNC0000_2415	*pfkB*							-1		-1				6-phosphofructokinase II
BAA196NC_1980	HMPRNC0000_1811	*thrS*								1		1			Threonyl-tRNA synthetase
BAA196NC_1982	HMPRNC0000_1808	*rpmI*			-1										50S ribosomal protein L35
BAA196NC_1984	HMPRNC0000_1185	*pheS*									-1				Phenylalanyl-tRNA synthetase alpha subunit
BAA196NC_1998	HMPRNC0000_0253	*menE*								-1		-1			Long-chain-fatty-acid--CoA ligase
BAA196NC_2006	HMPRNC0000_0254	*ydiF*								-1	1	-1		-1	Fused predicted acetyl-CoA:acetoacetyl-CoA transferase: alpha subunit/beta subunit
BAA196NC_2008	HMPRNC0000_1729	*ydiB*											-1		Quinate/shikimate 5-dehydrogenase, NAD(P)-binding
BAA196NC_2022	HMPRNC0000_0938	*sufS*						-1							Selenocysteine lyase
BAA196NC_2039	HMPRNC0000_1912	*ribC*						1							Riboflavin synthase subunit alpha
BAA196NC_2052	HMPRNC0000_0986	*nemA*		1		1		1	1		1				N-ethylmaleimide reductase, FMN-linked
BAA196NC_2055	HMPRNC0000_0756	*ydhF*											-1		Predicted oxidoreductase
BAA196NC_2092	HMPRNC0000_1996	*fumC*							-1		-1				Fumarate hydratase
BAA196NC_2163	HMPRNC0000_2754	*ydfG*						1							L-allo-threonine dehydrogenase, NAD(P)-binding
BAA196NC_2253	HMPRNC0000_2406	*yncB*						1	1						Predicted oxidoreductase, Zn-dependent and NAD(P)-binding
BAA196NC_2276	HMPRNC0000_0376	*rimL*							1						Ribosomal-protein-L7/L12-serine acetyltransferase
BAA196NC_2288	HMPRNC0000_2317	*aldA*		1											Aldehyde dehydrogenase A, NAD-linked
BAA196NC_2291	HMPRNC0000_0226	*azoR*	1					1			1				Acyl carrier protein phosphodiesterase
BAA196NC_2361	HMPRNC0000_0093	*abgA*													Predicted peptidase, aminobenzoyl-glutamate utilization protein
BAA196NC_2361	HMPRNC0000_1505	.											-1		N-acetyl-L,L-diaminopimelate deacetylase
BAA196NC_2376	HMPRNC0000_1849	*tpx*						1							Thiol peroxidase
BAA196NC_2385	HMPRNC0000_0232	*ycjS*							-1						Predicted oxidoreductase, NADH-binding
BAA196NC_2412	HMPRNC0000_1044	*fabI*					1								Enoyl-(acyl carrier protein) reductase
BAA196NC_2420	HMPRNC0000_1257	*pyrF*								1		1			Orotidine 5'-phosphate decarboxylase
BAA196NC_2425	HMPRNC0000_1449	*acnA*		1					-1		-1				Aconitate hydratase
BAA196NC_2428	HMPRNC0000_1315	*topA*								1		1			DNA topoisomerase I
BAA196NC_2440	HMPRNC0000_1473	*trpB*		1		1				-1		-1			Tryptophan synthase subunit beta
BAA196NC_2441	HMPRNC0000_1474	*trpA*		1		1				-1		-1			Tryptophan synthase subunit alpha
BAA196NC_2454	HMPRNC0000_1022	*oppF*							-1		-1		-1	-1	Oligopeptide transporter subunit
BAA196NC_2455	HMPRNC0000_1021	*oppD*		1					-1	-1	-1	-1	-1	-1	Oligopeptide transporter ATP-binding component
BAA196NC_2456	HMPRNC0000_1020	*oppC*							-1	-1	-1	-1	-1	-1	Oligopeptide transporter subunit
BAA196NC_2457	HMPRNC0000_1019	*oppB*							-1		-1		-1	-1	Oligopeptide permease ABC transporter membrane protein
BAA196NC_2458	HMPRNC0000_1023	*oppA*	1						-1	-1	-1	-1		-1	Oligopeptide transporter subunit
BAA196NC_2473	HMPRNC0000_2652	*narH*					-1		-1		-1				Nitrate reductase 1, beta (Fe-S) subunit
BAA196NC_2474	HMPRNC0000_2655	*narG*		1		1		1	-1		-1			-1	Nitrate reductase 1, alpha subunit
BAA196NC_2475	HMPRNC0000_2644	*narK*						1	-1		-1				Nitrate/nitrite transporter
BAA196NC_2478	HMPRNC0000_2068	*narL*						1							DNA-binding response regulator in two-component regulatory system with NarX (or NarQ)
BAA196NC_2497	HMPRNC0000_0517	*ispE*						-1	1				-1		4-diphosphocytidyl-2-C-methyl-D-erythritol kinase
BAA196NC_2502	HMPRNC0000_0407	*ychF*					1								Translation-associated GTPase
BAA196NC_2565	HMPRNC0000_1825	*icd*		1		1	-1		-1		-1			1	Isocitrate dehydrogenase
BAA196NC_2568	HMPRNC0000_1753	*trmU*							1						TRNA (5-methylaminomethyl-2-thiouridylate)-methyltransferase
BAA196NC_2589	HMPRNC0000_0525	*mfd*								1		1	-1		Transcription-repair coupling factor
BAA196NC_2594	HMPRNC0000_0966	*ndh*						1							Respiratory NADH dehydrogenase 2/cupric reductase
BAA196NC_2610	HMPRNC0000_1292	*acpP*		-1			1								Acyl carrier protein
BAA196NC_2611	HMPRNC0000_1290	*fabG*	-1										1		3-oxoacyl-[acyl-carrier-protein] reductase
BAA196NC_2612	HMPRNC0000_1289	*fabD*								1		1			Acyl carrier protein S-malonyltransferase
BAA196NC_2613	HMPRNC0000_1015	*fabH*								1		1			3-oxoacyl-(acyl carrier protein) synthase
BAA196NC_2682	HMPRNC0000_0380	*ycdB*							1						Hypothetical protein
BAA196NC_2685	HMPRNC0000_2087	*putP*							-1						Proline:sodium symporter
BAA196NC_2710	HMPRNC0000_1509	*cspG*						-1	-1				-1	-1	DNA-binding transcriptional regulator
BAA196NC_2731	HMPRNC0000_1511	*yccX*						1							Predicted acylphosphatase
BAA196NC_2755	HMPRNC0000_2876	*pyrD*							-1						Dihydroorotate dehydrogenase
BAA196NC_2766	HMPRNC0000_0177	*ssuC*							1		1				Alkanesulfonate transporter subunit
BAA196NC_2767	HMPRNC0000_0175	*ssuB*							1		1				Alkanesulfonate transporter subunit
BAA196NC_2773	HMPRNC0000_1674	*ycbL*						-1							Predicted metal-binding enzyme
BAA196NC_2786	HMPRNC0000_2045	*msbA*							1	1	1	1			Fused lipid transporter subunits of ABC superfamily: membrane component/ATP-binding component
BAA196NC_2796	HMPRNC0000_2662	*focA*					-1								Formate transporter
BAA196NC_2797	HMPRNC0000_0241	*pflB*	-1				-1								Pyruvate formate lyase I
BAA196NC_2808	HMPRNC0000_1758	*ycaJ*						-1							Recombination protein
BAA196NC_2812	HMPRNC0000_0851	*trxB*							1	1		1			Thioredoxin reductase, FAD/NAD(P)-binding
BAA196NC_2828	HMPRNC0000_2817	*poxB*		1						-1		-1			Pyruvate dehydrogenase
BAA196NC_2842	HMPRNC0000_1142	*potI*							-1						Putrescine transporter subunit: membrane component of ABC superfamily
BAA196NC_2850	HMPRNC0000_0426	*nfsA*							1						Nitroreductase A, NADPH-dependent, FMN-dependent
BAA196NC_2882	HMPRNC0000_1497	*ybiT*					1								Fused predicted transporter subunits of ABC superfamily: ATP-binding components
BAA196NC_2890	HMPRNC0000_2340	*dps*	-1		-1			1	1		1			1	DNA protection during starvation conditions

BAA196NC_2917	HMPRNC0000_2490	*moaE*							1	1		1			Molybdopterin synthase, large subunit
BAA196NC_2919	HMPRNC0000_2494	*moaC*				1									Molybdenum cofactor biosynthesis protein C
BAA196NC_2920	HMPRNC0000_2495	*moaB*				1		1							Molybdopterin biosynthesis protein B
BAA196NC_2921	HMPRNC0000_2486	*moaA*			1				1	1		1			Molybdenum cofactor biosynthesis protein A
BAA196NC_2923	HMPRNC0000_0842	*uvrB*											-1		Excinuclease ABC subunit B
BAA196NC_2928	HMPRNC0000_2696	*bioA*						-1							Adenosylmethionine--8-amino-7-oxononanoate transaminase
BAA196NC_3009	HMPRNC0000_2679	*gpmA*						1					-1		Phosphoglyceromutase
BAA196NC_3012	HMPRNC0000_2350	*zitB*							1	1	1	1	1	1	Zinc transporter ZitB
BAA196NC_3025	HMPRNC0000_1124	*cydA*								1		1			Cytochrome d terminal oxidase, subunit I
BAA196NC_3030	HMPRNC0000_1313	*sucD*		1				-1	-1		-1				Succinyl-CoA synthetase subunit alpha
BAA196NC_3031	HMPRNC0000_1312	*sucC*		1	-1		-1	-1	-1		-1				Succinyl-CoA synthetase subunit beta
BAA196NC_3032	HMPRNC0000_1519	*sucB*		1	-1		-1	-1	-1		-1				Dihydrolipoamide acetyltransferase
BAA196NC_3033	HMPRNC0000_1520	*sucA*		1			-1	-1	-1		-1				Alpha-ketoglutarate decarboxylase
BAA196NC_3034	HMPRNC0000_1198	*sdhB*		1			-1	-1	-1		-1			1	Succinate dehydrogenase, FeS subunit
BAA196NC_3035	HMPRNC0000_1197	*sdhA*		1		1	-1	-1	-1		-1			1	Succinate dehydrogenase flavoprotein subunit
BAA196NC_3037	HMPRNC0000_1195	*sdhC*				-1		-1	-1						Succinate dehydrogenase cytochrome b556 large membrane subunit
BAA196NC_3046	HMPRNC0000_1739	*ybgK*		1											Predicted enzyme subunit
BAA196NC_3047	HMPRNC0000_1740	*ybgJ*				1									Predicted enzyme subunit
BAA196NC_3050	HMPRNC0000_0760	*phr*						-1	-1						Deoxyribodipyrimidine photolyase, FAD-binding
BAA196NC_3061	HMPRNC0000_2263	*kdpB*						-1						1	Potassium-transporting ATPase subunit B
BAA196NC_3062	HMPRNC0000_2262	*kdpC*						-1						1	Potassium-transporting ATPase subunit C
BAA196NC_3064	HMPRNC0000_2267	*kdpE*						-1							DNA-binding response regulator in two-component regulatory system with KdpD
BAA196NC_3080	HMPRNC0000_2816	*nagE*											-1		Fused N-acetyl glucosamine specific PTS enzyme: IIC, IIB , and IIA components
BAA196NC_3088	HMPRNC0000_1359	*miaB*					1								Isopentenyl-adenosine A37 tRNA methylthiolase
BAA196NC_3098	HMPRNC0000_0263	*rihA*					-1								Ribonucleoside hydrolase 1
BAA196NC_3107	HMPRNC0000_1903	*leuS*								1		1			Leucyl-tRNA synthetase
BAA196NC_3115	HMPRNC0000_2273	*mrdB*							-1				-1		Cell wall shape-determining protein
BAA196NC_3126	HMPRNC0000_1509	*cspE*		-1					-1				-1	-1	Cold shock protein E
BAA196NC_3143	HMPRNC0000_0424	*ahpF*					1							1	Alkyl hydroperoxide reductase, F52a subunit, FAD/NAD(P)-binding
BAA196NC_3160	HMPRNC0000_0710	*fepD*							1						Iron-enterobactin transporter subunit
BAA196NC_3162	HMPRNC0000_0708	*fepC*							1		1				Iron-enterobactin transporter subunit
BAA196NC_3175	HMPRNC0000_2797	*nfnB*							1		1			1	Dihydropteridine reductase, NAD(P)H-dependent, oxygen-insensitive
BAA196NC_3229	HMPRNC0000_0573	*cysS*					1		1						Cysteinyl-tRNA synthetase
BAA196NC_3230	HMPRNC0000_0984	*ppiB*					1	1					-1		Peptidyl-prolyl cis-trans isomerase B (rotamase B)
BAA196NC_3232	HMPRNC0000_1099	*purE*							-1	-1	-1	-1			Phosphoribosylaminoimidazole carboxylase catalytic subunit
BAA196NC_3233	HMPRNC0000_1100	*purK*					-1		-1		-1				Phosphoribosylaminoimidazole carboxylase
BAA196NC_3241	HMPRNC0000_2704	*glxK*											-1		Glycerate kinase II
BAA196NC_3243	HMPRNC0000_1254	*allB*							-1	1	-1	1			Allantoinase
BAA196NC_3262	HMPRNC0000_2727	*ybbM*								-1		-1			putative iron export permease protein FetB
BAA196NC_3269	HMPRNC0000_2838	*copA*	1	1	1	1	1		1	1	1	1			Copper transporter
BAA196NC_3285	HMPRNC0000_1767	*apt*					1								Adenine phosphoribosyltransferase
BAA196NC_3314	HMPRNC0000_1588	*hupB*				-1			-1	-1		-1		1	HU, DNA-binding transcriptional regulator, beta subunit
BAA196NC_3317	HMPRNC0000_0856	*clpP*							1	1	1	1		1	ATP-dependent Clp protease proteolytic subunit
BAA196NC_3324	HMPRNC0000_1094	*cyoB*	1	1			-1								Cytochrome o ubiquinol oxidase subunit I
BAA196NC_3327	HMPRNC0000_1158	*cyoE*	1	1											Protoheme IX farnesyltransferase
BAA196NC_3349	HMPRNC0000_1770	*tgt*								1		1			Queuine tRNA-ribosyltransferase
BAA196NC_3354	HMPRNC0000_0191	*brnQ*							-1		-1				Predicted branched chain amino acid transporter (LIV-II)
BAA196NC_3356	HMPRNC0000_1610	*phoB*			1				-1						DNA-binding response regulator in two-component regulatory system with PhoR (or CreC)
BAA196NC_3366	HMPRNC0000_1666	*aroL*							-1	-1	-1	-1	1		Shikimate kinase II
BAA196NC_3387	HMPRNC0000_1796	*hemB*				1	1								Delta-aminolevulinic acid dehydratase
BAA196NC_3399	HMPRNC0000_2275	*frmR*						1							Regulator protein that represses frmRAB operon
BAA196NC_3423	HMPRNC0000_1826	*prpC*							-1		-1		1		2-methylcitrate synthase
BAA196NC_3442	HMPRNC0000_2404	*betT*				1								-1	Choline transporter of high affinity
BAA196NC_3444	HMPRNC0000_2903	*betB*									-1				Betaine aldehyde dehydrogenase, NAD-dependent
BAA196NC_3459	HMPRNC0000_2315	*ykgM*						1	-1						50S ribosomal protein L31
BAA196NC_3510	HMPRNC0000_2080	*dinB*								1		1			DNA polymerase IV
BAA196NC_3522	HMPRNC0000_2205	*yafV*							1		1				Predicted C-N hydrolase family amidase, NAD(P)-binding
BAA196NC_3536	HMPRNC0000_0477	*metN*							1		1				DL-methionine transporter subunit
BAA196NC_3537	HMPRNC0000_0478	*metI*							1		1				DL-methionine transporter subunit
BAA196NC_3538	HMPRNC0000_0479	*metQ*							1		1				DL-methionine transporter subunit
BAA196NC_3541	HMPRNC0000_1327	*proS*								1		1			Prolyl-tRNA synthetase
BAA196NC_3556	HMPRNC0000_2291	*fabZ*				1		1							(3R)-hydroxymyristoyl ACP dehydratase
BAA196NC_3565	HMPRNC0000_1323	*frr*							-1						Ribosome releasing factor
BAA196NC_3566	HMPRNC0000_1322	*pyrH*			1				-1		-1				Uridylate kinase
BAA196NC_3571	HMPRNC0000_2072	*map*											-1		Methionine aminopeptidase
BAA196NC_3582	HMPRNC0000_0965	*yadR*						1	1		1				Hypothetical protein
BAA196NC_3604	HMPRNC0000_2886	*panB*							1		1				3-methyl-2-oxobutanoate hydroxymethyltransferase
BAA196NC_3605	HMPRNC0000_2885	*panC*							1		1				Pantoate--beta-alanine ligase
BAA196NC_3633	HMPRNC0000_1433	*guaC*									-1				Guanosine 5'-monophosphate oxidoreductase
BAA196NC_3634	HMPRNC0000_1816	*coaE*							1	1		1			Dephospho-CoA kinase
BAA196NC_3647	HMPRNC0000_1154	*ftsW*		-1		-1									Integral membrane protein involved in stabilizing FstZ ring during cell division
BAA196NC_3648	HMPRNC0000_1232	*murD*							-1		-1				UDP-N-acetylmuramoyl-L-alanyl-D-glutamate synthetase
BAA196NC_3649	HMPRNC0000_1231	*mraY*							-1		-1				Phospho-N-acetylmuramoyl-pentapeptide- transferase
BAA196NC_3650	HMPRNC0000_2269	*murF*						-1							UDP-N-acetylmuramoyl-tripeptide:D-alanyl-D- alanine ligase

BAA196NC_3651	HMPRNC0000_1053	*murE*						-1					-1		UDP-N-acetylmuramoylalanyl-D-glutamate--2, 6-diaminopimelate ligase
BAA196NC_3654	HMPRNC0000_1229	*mraW*							-1	-1	-1	-1	-1		S-adenosyl-methyltransferase
BAA196NC_3655	HMPRNC0000_1228	*mraZ*				-1			-1	-1		-1	-1	-1	Hypothetical protein
BAA196NC_3658	HMPRNC0000_2233	*ilvI*							1		1				Acetolactate synthase III large subunit
BAA196NC_3660	HMPRNC0000_2236	*leuA*							1		1				2-isopropylmalate synthase
BAA196NC_3661	HMPRNC0000_2239	*leuB*							1						3-isopropylmalate dehydrogenase
BAA196NC_3662	HMPRNC0000_2240	*leuC*							1		1				Isopropylmalate isomerase large subunit
BAA196NC_3684	HMPRNC0000_0515	*ksgA*											-1		Dimethyladenosine transferase
BAA196NC_3688	HMPRNC0000_1043	*kefC*								1		1			Glutathione-regulated potassium-efflux system protein
BAA196NC_3703	HMPRNC0000_1256	*carB*	1	1	1		-1	-1	-1	1		1	1		Carbamoyl-phosphate synthase large subunit
BAA196NC_3704	HMPRNC0000_1255	*carA*	1		1		-1		-1	1		1	1		Carbamoyl-phosphate synthase small subunit
BAA196NC_3712	HMPRNC0000_1716	*rpsT*		-1		-1									30S ribosomal protein S20
BAA196NC_3720	HMPRNC0000_1708	*dnaJ*							1	1	1	1			Chaperone Hsp40, co-chaperone with DnaK
BAA196NC_3731	HMPRNC0000_1425	*thrB*									1			-1	Homoserine kinase
BAA196NC_3740	HMPRNC0000_0892	*ytjC*									1				Phosphoglycerate mutase
BAA196NC_3744	HMPRNC0000_0794	*yjjK*							1	1	1	1			Fused predicted transporter subunits of ABC superfamily: ATP-binding components
BAA196NC_3752	HMPRNC0000_2339	*deoD*									-1				Purine nucleoside phosphorylase
BAA196NC_3753	HMPRNC0000_0135	*deoB*		1											Phosphopentomutase
BAA196NC_3783	HMPRNC0000_0463	*yjiA*				1									Predicted GTPase
BAA196NC_3845	HMPRNC0000_2771	*yjhF*								1		1	-1		KpLE2 phage-like element; predicted transporter
BAA196NC_3880	HMPRNC0000_1788	*valS*						-1		1	1	1			Valyl-tRNA synthetase
BAA196NC_3891	HMPRNC0000_1253	*pyrB*	1	1	1		-1	-1	-1		-1				Aspartate carbamoyltransferase catalytic subunit
BAA196NC_3893	HMPRNC0000_0519	*yjgF*	1		1	-1									Ketoacid-binding protein
BAA196NC_3896	HMPRNC0000_2632	*treB*							1	1	1	1			Fused trehalose(maltose)-specific PTS enzyme: IIB component/IIC component
BAA196NC_3897	HMPRNC0000_0491	*treC*								1		1	-1		Trehalose-6-P hydrolase
BAA196NC_3898	HMPRNC0000_2908	*nrdD*						-1							Anaerobic ribonucleoside triphosphate reductase
BAA196NC_3903	HMPRNC0000_1885	*murC*												-1	UDP-N-acetylmuramate--L-alanine ligase
BAA196NC_3926	HMPRNC0000_2710	*cycA*							-1		-1				D-alanine/D-serine/glycine transporter
BAA196NC_3938	HMPRNC0000_0619	*ulaD*								-1		-1			3-keto-L-gulonate 6-phosphate decarboxylase
BAA196NC_3954	HMPRNC0000_0575	*rlmB*							1						23S rRNA (Gm2251)-methyltransferase
BAA196NC_3955	HMPRNC0000_0870	*rnr*	-1				-1		1	1		1			Exoribonuclease R, RNase R
BAA196NC_3957	HMPRNC0000_0018	*purA*					1								Adenylosuccinate synthetase
BAA196NC_3961	HMPRNC0000_1373	*hflX*							1						Predicted GTPase
BAA196NC_3977	HMPRNC0000_1197	*frdA*	-1				-1	-1	-1		-1			1	Fumarate reductase
BAA196NC_3978	HMPRNC0000_1198	*frdB*					-1	-1	-1		-1			1	Fumarate reductase (anaerobic), Fe-S subunit
BAA196NC_3986	HMPRNC0000_1654	*efp*					1								Elongation factor P
BAA196NC_3990	HMPRNC0000_2200	*groL*		1				1	1	1	1	1	1	1	Chaperonin GroEL
BAA196NC_3991	HMPRNC0000_2201	*groS*						1	1	1	1	1			Co-chaperonin GroES
BAA196NC_4003	HMPRNC0000_0543	*lysU*						1		1		1			Lysine tRNA synthetase, inducible
BAA196NC_4006	HMPRNC0000_2794	*yjdJ*						1							Predicted acyltransferase with acyl-CoA N-acyltransferase domain
BAA196NC_4026	HMPRNC0000_2879	*phnB*							-1				-1		Hypothetical protein
BAA196NC_4057	HMPRNC0000_2640	*gltP*							-1						Glutamate/aspartate:proton symporter
BAA196NC_4065	HMPRNC0000_1877	*acs*		1							-1				Acetyl-coenzyme A synthetase
BAA196NC_4078	HMPRNC0000_0843	*uvrA*						-1							Excinuclease ABC subunit A
BAA196NC_4095	HMPRNC0000_1436	*lexA*											-1		LexA repressor
BAA196NC_4104	HMPRNC0000_0229	*malE*							-1		-1				Maltose ABC transporter periplasmic protein
BAA196NC_4106	HMPRNC0000_0231	*malG*							-1						Maltose transporter subunit
BAA196NC_4128	HMPRNC0000_1111	*purH*			1				-1		-1			-1	Bifunctional phosphoribosylaminoimidazolecarboxamide formyltransferase/IMP cyclohydrolase
BAA196NC_4129	HMPRNC0000_1112	*purD*							-1		-1			-1	Phosphoribosylamine--glycine ligase
BAA196NC_4134	HMPRNC0000_1588	*hupA*				-1			-1	-1		-1		1	HU, DNA-binding transcriptional regulator, alpha subunit
BAA196NC_4137	HMPRNC0000_1977	*hemE*						-1							Uroporphyrinogen decarboxylase
BAA196NC_4141	HMPRNC0000_2282	*thiE*					-1		-1		-1				Thiamine-phosphate pyrophosphorylase
BAA196NC_4148	HMPRNC0000_0585	*rpoB*		1											DNA-directed RNA polymerase subunit beta
BAA196NC_4150	HMPRNC0000_0582	*rplJ*					1							-1	50S ribosomal protein L10
BAA196NC_4151	HMPRNC0000_0581	*rplA*								1		1			50S ribosomal protein L1
BAA196NC_4152	HMPRNC0000_0580	*rplK*					1								50S ribosomal protein L11
BAA196NC_4153	HMPRNC0000_0579	*nusG*					1								Transcription antitermination protein NusG
BAA196NC_4168	HMPRNC0000_0990	*argH*							1		1				Argininosuccinate lyase
BAA196NC_4171	HMPRNC0000_2193	*dapE*								-1		-1			Acetylornithine deacetylase
BAA196NC_4188	HMPRNC0000_0476	*metB*							1		1		1		Cystathionine gamma-synthase
BAA196NC_4192	HMPRNC0000_1267	*priA*							1	1		1			Primosome assembly protein PriA
BAA196NC_4195	HMPRNC0000_1317	*hslV*								1		1			ATP-dependent protease peptidase subunit
BAA196NC_4196	HMPRNC0000_1318	*hslU*			1										ATP-dependent protease ATP-binding subunit
BAA196NC_4197	HMPRNC0000_1072	*menA*											1		1,4-dihydroxy-2-naphthoate octaprenyltransferase
BAA196NC_4200	HMPRNC0000_1366	*glpF*							-1	-1	-1	-1			Glycerol facilitator
BAA196NC_4201	HMPRNC0000_1367	*glpK*							-1		-1			1	Glycerol kinase
BAA196NC_4208	HMPRNC0000_0863	*tpiA*							1		1			-1	Triosephosphate isomerase
BAA196NC_4216	HMPRNC0000_2578	*yiiM*						1							Hypothetical protein
BAA196NC_4218	HMPRNC0000_1682	*sodA*		1										1	Superoxide dismutase, Mn
BAA196NC_4229	HMPRNC0000_2729	*frvX*							1						Predicted endo-1,4-beta-glucanase
BAA196NC_4232	HMPRNC0000_2501	*fdhD*							1		1				Formate dehydrogenase accessory protein
BAA196NC_4259	HMPRNC0000_1379	*glnA*	1		1		-1								Glutamine synthetase
BAA196NC_4265	HMPRNC0000_1801	*yihA*					1		1						GTP-binding protein
BAA196NC_4267	HMPRNC0000_1818	*polA*							1						DNA polymerase I
BAA196NC_4295	HMPRNC0000_0398	*metE*						-1							5-methyltetrahydropteroyltriglutamate-- homocysteine methyltransferase

BAA196NC_4301	HMPRNC0000_0795	*recQ*						-1	1						ATP-dependent DNA helicase
BAA196NC_4309	HMPRNC0000_2089	*uvrD*						-1							DNA-dependent ATPase I and helicase II
BAA196NC_4318	HMPRNC0000_1798	*hemC*							1	1		1			Porphobilinogen deaminase
BAA196NC_4335	HMPRNC0000_2303	*rffE*							-1						UDP-N-acetyl glucosamine-2-epimerase
BAA196NC_4340	HMPRNC0000_1193	*trxA*					1		1					1	Thioredoxin
BAA196NC_4349	HMPRNC0000_2231	*ilvD*							1		1				Dihydroxy-acid dehydratase
BAA196NC_4357	HMPRNC0000_2597	*hsrA*									1				Predicted multidrug or homocysteine efflux system
BAA196NC_4359	HMPRNC0000_0289	*rbsK*					-1								Ribokinase
BAA196NC_4363	HMPRNC0000_0290	*rbsD*	-1	-1											Predicted cytoplasmic sugar-binding protein
BAA196NC_4370	HMPRNC0000_3027	*gidA*							1	1		1			Glucose-inhibited division protein A
BAA196NC_4373	HMPRNC0000_2301	*atpB*							-1						F0F1 ATP synthase subunit A
BAA196NC_4375	HMPRNC0000_2299	*atpF*		1					-1		-1				F0F1 ATP synthase subunit B
BAA196NC_4377	HMPRNC0000_2297	*atpA*		1					-1		-1				F0F1 ATP synthase subunit alpha
BAA196NC_4378	HMPRNC0000_2296	*atpG*		1											F0F1 ATP synthase subunit gamma
BAA196NC_4379	HMPRNC0000_2295	*atpD*		1							-1				F0F1 ATP synthase subunit beta
BAA196NC_4380	HMPRNC0000_2294	*atpC*							-1		-1				F0F1 ATP synthase subunit epsilon
BAA196NC_4381	HMPRNC0000_0521	*glmU*					1								Bifunctional N-acetylglucosamine-1-phosphate uridyltransferase/glucosamine-1-phosphate acetyltransferase
BAA196NC_4387	HMPRNC0000_1486	*phoU*			1										Negative regulator of PhoR/PhoB two-component regulator
BAA196NC_4405	HMPRNC0000_3029	*trmE*						1	1	1		1			TRNA modification GTPase
BAA196NC_4478	HMPRNC0000_2586	.									-1				Sodium/glutamate symporter
BAA196NC_0005	NA	*yidB*						1							Hypothetical protein
BAA196NC_0017	NA	*ibpA*				1		1							Heat shock chaperone
BAA196NC_0018	NA	*ibpB*						1							Heat shock chaperone
BAA196NC_0034	NA	*uhpA*						-1							DNA-binding response regulator in two-component regulatory system wtih UhpB
BAA196NC_0055	NA	*rpoZ*					1								DNA-directed RNA polymerase subunit omega
BAA196NC_0060	NA	*yicC*		-1			1								Hypothetical protein
BAA196NC_0067	NA	*rpmB*		-1		-1									50S ribosomal protein L28
BAA196NC_0077	NA	.						-1							Lipopolysaccharide 1,6-galactosyltransferase
BAA196NC_0090	NA	*tdh*	1	1		1									L-threonine 3-dehydrogenase
BAA196NC_0106	NA	*mtlR*		-1											DNA-binding repressor
BAA196NC_0139	NA	*bax*					1								Hypothetical protein
BAA196NC_0155	NA	*cspA*	-1	-1	-1	-1		-1							Major cold shock protein
BAA196NC_0156	NA	*yiaG*						1							Predicted transcriptional regulator
BAA196NC_0157	NA	*yiaF*	-1					1							Hypothetical protein
BAA196NC_0164	NA	*yhjX*					-1								Predicted transporter
BAA196NC_0188	NA	*kdgK*					1								Ketodeoxygluconokinase
BAA196NC_0198	NA	*gadX*		-1				1							DNA-binding transcriptional dual regulator
BAA196NC_0201	NA	*gadW*		-1											DNA-binding transcriptional activator
BAA196NC_0203	NA	*mdtE*		1			-1								Multidrug resistance efflux transporter
BAA196NC_0205	NA	*gadE*						1							DNA-binding transcriptional activator
BAA196NC_0206	NA	*hdeD*						1							Acid-resistance membrane protein
BAA196NC_0208	NA	*hdeB*		-1			1								Acid-resistance protein
BAA196NC_0209	NA	*yhiD*		-1											Predicted Mg(2+) transport ATPase inner membrane protein
BAA196NC_0211	NA	*slp*						1							Outer membrane lipoprotein
BAA196NC_0217	NA	*gorA*			1										Glutathione reductase
BAA196NC_0223	NA	*yhiO*						1							Universal stress protein UspB
BAA196NC_0226	NA	*yhiM*						1							Conserved inner membrane protein
BAA196NC_0257	NA	*rpoH*			1		1								RNA polymerase sigma factor
BAA196NC_0281	NA	*yhhW*	1	1	1	1	1	1							Quercetin 2,3-dioxygenase
BAA196NC_0282	NA	*gntR*			1										DNA-binding transcriptional repressor
BAA196NC_0290	NA	*glgA*					-1								Glycogen synthase
BAA196NC_0302	NA	*malQ*						-1							4-alpha-glucanotransferase (amylomaltase)
BAA196NC_0322	NA	*nudE*					1								ADP-ribose diphosphatase
BAA196NC_0329	NA	*aroK*					1								Shikimate kinase I
BAA196NC_0358	NA	*fic*					-1								Stationary-phase protein, cell division
BAA196NC_0363	NA	*yhfA*					1								Hypothetical protein
BAA196NC_0381	NA	*tufA*			-1										Protein chain elongation factor EF-Tu (duplicate of tufB)
BAA196NC_0384	NA	*bfr*		-1	-1	-1									Bacterioferritin, iron storage and detoxification protein
BAA196NC_0427	NA	*yhdN*						1							Hypothetical protein
BAA196NC_0429	NA	*yhdL*				1									Hypothetical protein
BAA196NC_0454	NA	*dusB*	-1					-1							tRNA-dihydrouridine synthase B
BAA196NC_0464	NA	*mreC*						-1							Cell wall structural complex MreBCD transmembrane component MreC
BAA196NC_0474	NA	*yhcO*		-1											Predicted barnase inhibitor
BAA196NC_0475	NA	*yhcN*						1							Hypothetical protein
BAA196NC_0514	NA	*kdsC*					-1								3-deoxy-D-manno-octulosonate 8-phosphate phosphatase
BAA196NC_0519	NA	*yrbD*			1										Predicted ABC-type organic solvent transporter
BAA196NC_0523	NA	*murA*					1								UDP-N-acetylglucosamine 1-carboxyvinyltransferase
BAA196NC_0528	NA	*yhbE*					1								Conserved inner membrane protein
BAA196NC_0532	NA	*greA*					1								Transcription elongation factor GreA
BAA196NC_0538	NA	*secG*					1								Protein-export membrane protein
BAA196NC_0547	NA	*rpsO*				1									30S ribosomal protein S15
BAA196NC_0550	NA	*deaD*						-1							ATP-dependent RNA helicase
BAA196NC_0558	NA	*yhbP*				1									Hypothetical protein
BAA196NC_0582	NA	*yhaV*		-1		-1									Hypothetical protein
BAA196NC_0589	NA	*yhaC*						-1							Uncharacterized protein
BAA196NC_0593	NA	*tdcA*					-1								DNA-binding transcriptional activator
BAA196NC_0595	NA	*tdcC*					-1								L-threonine/L-serine transporter

BAA196NC_0596	NA	*tdcD*				-1		-1		Propionate kinase/acetate kinase C, anaerobic
BAA196NC_0597	NA	*tdcE*					-1	-1		Pyruvate formate-lyase 4/2-ketobutyrate formate-lyase
BAA196NC_0598	NA	*tdcF*						-1		Predicted L-PSP (mRNA) endoribonuclease
BAA196NC_0601	NA	*yhaM*			1			-1		Hypothetical protein
BAA196NC_0608	NA	*yqjF*	1				1	1		Predicted quinol oxidase subunit
BAA196NC_0610	NA	*yqjE*				1				Conserved inner membrane protein
BAA196NC_0623	NA	*ygjR*			-1		-1			Predicted NAD(P)-binding dehydrogenase
BAA196NC_0629	NA	*fadH*		-1				-1		2,4-dienoyl-CoA reductase, NADH and FMN-linked
BAA196NC_0643	NA	*rpoD*	1		1					RNA polymerase sigma factor
BAA196NC_0644	NA	*dnaG*					1			DNA primase
BAA196NC_0645	NA	*rpsU*				-1	1			30S ribosomal protein S21
BAA196NC_0664	NA	*glgS*		-1						Glycogen synthesis protein GlgS
BAA196NC_0671	NA	*yqiC*		-1	-1	-1				Hypothetical protein
BAA196NC_0675	NA	*zupT*			1			1		Predicted dioxygenase
BAA196NC_0684	NA	*ygiN*		-1						Quinol monooxygenase
BAA196NC_0689	NA	*ygiV*				1				Predicted transcriptional regulator
BAA196NC_0700	NA	*yqhD*		1	1					Alcohol dehydrogenase, NAD(P)-dependent
BAA196NC_0704	NA	*exbB*					1			Membrane spanning protein in TonB-ExbB-ExbD complex
BAA196NC_0708	NA	*yghZ*					-1			Aldo-keto reductase
BAA196NC_0713	NA	*hybO*					-1	-1		Hydrogenase 2, small subunit
BAA196NC_0714	NA	*hybA*						-1		Hydrogenase 2 4Fe-4S ferredoxin-type component
BAA196NC_0716	NA	*hybC*						-1		Hydrogenase 2, large subunit
BAA196NC_0732	NA	*glcD*		1						Glycolate oxidase subunit, FAD-linked
BAA196NC_0733	NA	*glcE*		1		1				Glycolate oxidase FAD binding subunit
BAA196NC_0734	NA	*glcF*		1		1				Glycolate oxidase iron-sulfur subunit
BAA196NC_0735	NA	*glcG*		1						Hypothetical protein
BAA196NC_0736	NA	*glcB*		1		1				Malate synthase
BAA196NC_0746	NA	*nupG*						1		Nucleoside transporter
BAA196NC_0751	NA	*yggL*		-1						Hypothetical protein
BAA196NC_0753	NA	*ansB*		-1	-1		-1			Periplasmic L-asparaginase II
BAA196NC_0762	NA	*yqgE*				1				Hypothetical protein
BAA196NC_0774	NA	*tktA*		1						Transketolase 1, thiamin-binding
BAA196NC_0784	NA	*mscS*						1		Mechanosensitive channel
BAA196NC_0786	NA	*yggE*	1					1		Hypothetical protein
BAA196NC_0798	NA	*gcvH*	1		1	1				Glycine cleavage system protein H
BAA196NC_0801	NA	*pepP*						-1		Proline aminopeptidase P II
BAA196NC_0802	NA	*ubiH*		1						2-octaprenyl-6-methoxyphenyl hydroxylase
BAA196NC_0803	NA	*visC*						-1		Hypothetical protein
BAA196NC_0807	NA	*ygfF*						-1		Predicted NAD(P)-binding oxidoreductase with NAD(P)-binding Rossmann-fold domain
BAA196NC_0811	NA	*ygfZ*			1		1			Putative global regulator
BAA196NC_0839	NA	*ygeV*					-1			Predicted DNA-binding transcriptional regulator
BAA196NC_0876	NA	*ygdR*		-1			-1			Hypothetical protein
BAA196NC_0889	NA	*recC*						-1		Exonuclease V (RecBCD complex), gamma chain
BAA196NC_0890	NA	*ptr*					1			Protease III
BAA196NC_0899	NA	*ygdI*		-1				1		Hypothetical protein
BAA196NC_0900	NA	*fucK*		-1						L-fuculokinase
BAA196NC_0913	NA	*sdaC*						1		Predicted serine transporter
BAA196NC_0914	NA	*ygdH*					-1			Hypothetical protein
BAA196NC_0927	NA	*chpR*				1		1		Antitoxin of the ChpA-ChpR toxin-antitoxin system
BAA196NC_0959	NA	*cysD*			1					Sulfate adenylyltransferase subunit 2
BAA196NC_0966	NA	*truD*		1						tRNA pseudouridine synthase D
BAA196NC_0969	NA	*nlpD*		1		1				Predicted outer membrane lipoprotein
BAA196NC_0970	NA	*rpoS*		1			1			RNA polymerase sigma factor
BAA196NC_0983	NA	*hypD*						-1		Protein required for maturation of hydrogenases
BAA196NC_0985	NA	*hypB*					-1			GTP hydrolase involved in nickel liganding into hydrogenases
BAA196NC_1004	NA	*norR*						1		Anaerobic nitric oxide reductase transcription regulator
BAA196NC_1017	NA	*csrA*		-1	-1	-1				Carbon storage regulator
BAA196NC_1031	NA	*proX*		1						Glycine betaine transporter subunit
BAA196NC_1040	NA	*ygaW*						1		Predicted inner membrane protein
BAA196NC_1044	NA	*yqaE*						1		Predicted membrane protein
BAA196NC_1046	NA	*csiR*				1				DNA-binding transcriptional dual regulator
BAA196NC_1049	NA	*gabD*						-1		Succinate-semialdehyde dehydrogenase I, NADP-dependent
BAA196NC_1051	NA	*ygaT*		1						Hypothetical protein
BAA196NC_1082	NA	*yfjF*	-1		-1					Uncharacterized protein
BAA196NC_1104	NA	*pheA*						1		Fused chorismate mutase P/prephenate dehydratase
BAA196NC_1105	NA	*yfiA*			-1		-1			Cold shock protein associated with 30S ribosomal subunit
BAA196NC_1113	NA	*yfiQ*					-1			Fused predicted acyl-CoA synthetase: NAD(P)-binding subunit/ATP-binding subunit
BAA196NC_1115	NA	*trxC*						1		Thioredoxin 2
BAA196NC_1118	NA	*yfiD*	-1	-1	-1	-1	-1	1		Pyruvate formate lyase subunit
BAA196NC_1124	NA	*rpoE*					1			RNA polymerase, sigma 24 (sigma E) factor
BAA196NC_1126	NA	*rseB*			1					Periplasmic negative regulator of sigmaE
BAA196NC_1133	NA	*pdxJ*				1				Pyridoxal phosphate biosynthetic protein
BAA196NC_1137	NA	*yfhB*						1		Hypothetical protein
BAA196NC_1141	NA	*purL*		-1						Phosphoribosylformylglycinamidine synthase
BAA196NC_1145	NA	*glnB*					1			Regulatory protein P-II for glutamine synthetase
BAA196NC_1168	NA	*iscS*		1		1				Cysteine desulfurase
BAA196NC_1175	NA	*pepB*			1					Aminopeptidase B
BAA196NC_1223	NA	*nlpB*		1						Lipoprotein

BAA196NC_1237	NA	*talA*		1						Transaldolase A
BAA196NC_1238	NA	*maeB*		1		1				Malic enzyme
BAA196NC_1261	NA	*eutC*						-1		Ethanolamine ammonia-lyase small subunit
BAA196NC_1275	NA	*ucpA*			-1		-1			Short chain dehydrogenase
BAA196NC_1300	NA	*yfeC*				-1				Predicted DNA-binding transcriptional regulator
BAA196NC_1318	NA	*yfdY*						1		Predicted inner membrane protein
BAA196NC_1325	NA	*evgS*		-1			-1			Hybrid sensory histidine kinase in two-component regulatory system with EvgA
BAA196NC_1351	NA	*yfcZ*		-1		-1				Hypothetical protein
BAA196NC_1352	NA	*yfcY*				-1		-1		Acetyl-CoA acetyltransferase
BAA196NC_1354	NA	*sixA*					1			Phosphohistidine phosphatase
BAA196NC_1364	NA	*yfcN*		-1						Hypothetical protein
BAA196NC_1372	NA	*fabB*					1			3-oxoacyl-(acyl carrier protein) synthase
BAA196NC_1375	NA	*flk*						-1		Predicted flagella assembly protein
BAA196NC_1383	NA	*cvpA*	1		1					Membrane protein required for colicin V production
BAA196NC_1399	NA	*pta*			1	-1		-1		Phosphate acetyltransferase
BAA196NC_1401	NA	*yfbV*		-1		-1				Hypothetical protein
BAA196NC_1402	NA	*yfbU*						1		Hypothetical protein
BAA196NC_1404	NA	*yfbS*						-1		Predicted transporter
BAA196NC_1407	NA	*irhA*		-1				1		DNA-binding transcriptional repressor of flagellar, motility and chemotaxis genes
BAA196NC_1411	NA	*nuoC*						-1		NADH:ubiquinone oxidoreductase, chain C,D
BAA196NC_1412	NA	*nuoE*						-1		NADH dehydrogenase subunit E
BAA196NC_1414	NA	*nuoG*						-1		NADH dehydrogenase subunit G
BAA196NC_1431	NA	*elaB*	-1		-1	-1		1		Hypothetical protein
BAA196NC_1432	NA	*menF*						-1		Menaquinone-specific isochorismate synthase
BAA196NC_1437	NA	*menE*						-1		O-succinylbenzoic acid--CoA ligase
BAA196NC_1438	NA	*pmrD*						-1		Polymyxin resistance protein B
BAA196NC_1483	NA	*rcsD*			-1					Phosphotransfer intermediate protein in two-component regulatory system with RcsBC
BAA196NC_1485	NA	*ompC*	-1		-1					Outer membrane porin protein C
BAA196NC_1495	NA	*napA*					-1			Nitrate reductase, periplasmic, large subunit
BAA196NC_1496	NA	*napG*						-1		Quinol dehydrogenase periplasmic component
BAA196NC_1499	NA	*napC*					-1			Nitrate reductase, cytochrome c-type, periplasmic
BAA196NC_1524	NA	*spr*					1			Predicted peptidase, outer membrane lipoprotein
BAA196NC_1560	NA	*cdd*		-1						Cytidine deaminase
BAA196NC_1575	NA	*yehX*						-1		Predicted transporter subunit: ATP-binding component of ABC superfamily
BAA196NC_1609	NA	*gatZ*	-1				-1			D-tagatose 1,6-bisphosphate aldolase 2, subunit
BAA196NC_1634	NA	*yegK*				1	-1	-1		Uncharacterized protein
BAA196NC_1635	NA	*yegJ*				1		-1		Uncharacterized protein
BAA196NC_1667	NA	*galF*					1			Predicted subunit with GalU
BAA196NC_1669	NA	*rfbD*		-1						DTDP-4-dehydrorhamnose reductase subunit, NAD(P)-binding, of dTDP-L-rhamnose synthase
BAA196NC_1702	NA	*yeeX*				-1	1			Hypothetical protein
BAA196NC_1709	NA	*flu*					-1	-1		CP4-44 prophage; antigen 43 (Ag43) phase-variable biofilm formation autotransporter
BAA196NC_1733	NA	*yedY*				1				Hypothetical protein
BAA196NC_1745	NA	*yodC*				1		1		Hypothetical protein
BAA196NC_1749	NA	*yodD*		-1		-1				Hypothetical protein
BAA196NC_1750	NA	*dsrB*		-1		-1				Hypothetical protein
BAA196NC_1791	NA	*yecH*		-1				1		Hypothetical protein
BAA196NC_1821	NA	*cutC*		-1				-1		Copper homeostasis protein
BAA196NC_1824	NA	*yecP*						-1		Predicted S-adenosyl-L-methionine-dependent methyltransferase
BAA196NC_1826	NA	*yecN*		-1						Predicted inner membrane protein
BAA196NC_1827	NA	*yecE*				-1				Hypothetical protein
BAA196NC_1830	NA	*nudB*				1				DATP pyrophosphohydrolase
BAA196NC_1832	NA	*ruvC*				1				Holliday junction resolvase
BAA196NC_1847	NA	*purT*			1					Phosphoribosylglycinamide formyltransferase 2
BAA196NC_1848	NA	*yebG*				-1				Conserved protein regulated by LexA
BAA196NC_1850	NA	*yebE*				1				Hypothetical protein
BAA196NC_1862	NA	*yebV*		-1						Hypothetical protein
BAA196NC_1869	NA	*htpX*		1	1	1		1		Heat shock protein HtpX
BAA196NC_1875	NA	*yobF*	-1	-1	-1	-1				Hypothetical protein
BAA196NC_1876	NA	*cspC*		-1			-1			Stress protein, member of the CspA-family
BAA196NC_1882	NA	*manX*					-1			Fused mannose-specific PTS enzymes: IIA component/IIB component
BAA196NC_1886	NA	*sdaA*				-1				L-serine deaminase I
BAA196NC_1890	NA	*yoaC*		-1		-1				Hypothetical protein
BAA196NC_1893	NA	*yeaZ*						-1		Predicted peptidase
BAA196NC_1895	NA	*fadD*				-1		-1		Acyl-CoA synthase
BAA196NC_1896	NA	*rnd*					1			Ribonuclease D
BAA196NC_1905	NA	*yeaQ*						1		Conserved inner membrane protein
BAA196NC_1913	NA	*yeaJ*						1		Predicted diguanylate cyclase
BAA196NC_1915	NA	*yeaH*						-1		Hypothetical protein
BAA196NC_1916	NA	*yeaG*					-1			Conserved protein with nucleoside triphosphate hydrolase domain
BAA196NC_1917	NA	*mipA*		1		1				Scaffolding protein for murein synthesizing machinery
BAA196NC_1953	NA	*astA*						-1		Arginine succinyltransferase
BAA196NC_1954	NA	*astD*					-1	-1		Succinylglutamic semialdehyde dehydrogenase
BAA196NC_1955	NA	*astB*					-1			Succinylarginine dihydrolase
BAA196NC_1956	NA	*astE*						-1		Succinylglutamate desuccinylase

BAA196NC_1962	NA	*chbB*		-1		-1				N,N'-diacetylchitobiose-specific enzyme IIB component of PTS
BAA196NC_1971	NA	*ydjN*					1			Predicted transporter
BAA196NC_1974	NA	*yniB*		-1						Predicted inner membrane protein
BAA196NC_1976	NA	*ydiZ*	1					1		Hypothetical protein
BAA196NC_1986	NA	*ihfA*		-1		-1				Integration host factor subunit alpha
BAA196NC_1989	NA	*btuD*						-1		Vitamin B12-transporter ATPase
BAA196NC_1997	NA	*pps*		1	1	1				Phosphoenolpyruvate synthase
BAA196NC_2021	NA	*sufD*						-1		Component of SufBCD complex
BAA196NC_2025	NA	*lpp*	-1		-1	-1	1	-1		Murein lipoprotein
BAA196NC_2027	NA	*fumD*		-1		-1				Fumarase D
BAA196NC_2043	NA	*purR*					1			DNA-binding transcriptional repressor, hypoxanthine-binding
BAA196NC_2053	NA	*ydhM*						1		Predicted DNA-binding transcriptional regulator
BAA196NC_2061	NA	*slyB*				1		1		Outer membrane lipoprotein
BAA196NC_2084	NA	*hdhA*						1		7-alpha-hydroxysteroid dehydrogenase
BAA196NC_2085	NA	*uidR*						-1		DNA-binding transcriptional repressor
BAA196NC_2100	NA	*pntA*		1						NAD(P) transhydrogenase subunit alpha
BAA196NC_2101	NA	*pntB*		1						Pyridine nucleotide transhydrogenase
BAA196NC_2105	NA	*ydgD*						1		Predicted peptidase
BAA196NC_2118	NA	*ynfC*		-1						Hypothetical protein
BAA196NC_2139	NA	*relB*						1		Qin prophage; bifunctional antitoxin of the RelE-RelB toxin-antitoxin system/ transcriptional repressor
BAA196NC_2141	NA	*hokD*						1		Qin prophage; small toxic polypeptide
BAA196NC_2146	NA	*cspB*						-1		Qin prophage; cold shock protein
BAA196NC_2164	NA	*dcp*		1				1		Dipeptidyl carboxypeptidase II
BAA196NC_2167	NA	*ydeH*				1				Hypothetical protein
BAA196NC_2170	NA	*marB*			1					Hypothetical protein
BAA196NC_2171	NA	*marA*						1		DNA-binding transcriptional dual activator of multiple antibiotic resistance
BAA196NC_2186	NA	*lsrB*				-1		-1		AI2 transporter
BAA196NC_2188	NA	*lsrC*		-1		-1				AI2 transporter
BAA196NC_2189	NA	*lsrA*			-1		-1			Fused AI2 transporter subunits of ABC superfamily: ATP-binding components
BAA196NC_2211	NA	*yddV*				-1				Predicted diguanylate cyclase
BAA196NC_2221	NA	*sra*		-1		-1				30S ribosomal subunit protein S22
BAA196NC_2233	NA	*narZ*						-1		Nitrate reductase 2 (NRZ), alpha subunit
BAA196NC_2234	NA	*narY*						-1		Nitrate reductase 2 (NRZ), beta subunit
BAA196NC_2250	NA	*yncE*				1				Hypothetical protein
BAA196NC_2261	NA	*ydcT*						-1		Predicted spermidine/putrescine transporter subunit
BAA196NC_2262	NA	*ydcS*						-1		Predicted spermidine/putrescine transporter subunit
BAA196NC_2266	NA	*tehB*		1		1		1		Putative S-adenosyl-L-methionine-dependent methyltransferase
BAA196NC_2277	NA	*ydcH*		-1				-1		Hypothetical protein
BAA196NC_2279	NA	*ydcJ*				-1				Hypothetical protein
BAA196NC_2287	NA	*ydbD*		-1				-1		Uncharacterized protein
BAA196NC_2325	NA	*hslJ*				-1				Heat-inducible protein
BAA196NC_2326	NA	*ydbJ*		-1						Hypothetical protein
BAA196NC_2328	NA	*ydbK*			1					Fused predicted pyruvate-flavodoxin oxidoreductase: conserved protein/conserved protein/FeS binding protein
BAA196NC_2331	NA	*uspF*				-1		1		Stress-induced protein, ATP-binding protein
BAA196NC_2355	NA	*dbpA*						-1		ATP-dependent RNA helicase, specific for 23S rRNA
BAA196NC_2359	NA	*ycjY*				1				Putative hydrolase
BAA196NC_2365	NA	*fnr*					1			DNA-binding transcriptional dual regulator, global regulator of anaerobic growth
BAA196NC_2392	NA	*pspE*			1					Thiosulfate:cyanide sulfurtransferase (rhodanese)
BAA196NC_2415	NA	*gmr*						1		Modulator of Rnase II stability
BAA196NC_2418	NA	*osmB*		-1			-1			Lipoprotein
BAA196NC_2424	NA	*ribA*		-1			1			GTP cyclohydrolase II protein
BAA196NC_2429	NA	*yciN*		-1						Hypothetical protein
BAA196NC_2442	NA	*yciG*		-1						Hypothetical protein
BAA196NC_2443	NA	*yciF*						1		Hypothetical protein
BAA196NC_2444	NA	*yciE*						1		Hypothetical protein
BAA196NC_2445	NA	*ompW*						-1		Outer membrane protein W
BAA196NC_2464	NA	*hns*		-1		-1				Global DNA-binding transcriptional dual regulator H-NS
BAA196NC_2465	NA	*galU*						1		Glucose-1-phosphate uridylyltransferase
BAA196NC_2467	NA	*rssA*		-1				1		Hypothetical protein
BAA196NC_2477	NA	*narX*						1		Sensory histidine kinase in two-component regulatory system with NarL
BAA196NC_2500	NA	*ychH*	-1		-1		-1			Predicted inner membrane protein
BAA196NC_2512	NA	*ymgE*						1		Predicted inner membrane protein
BAA196NC_2518	NA	*dadX*		1		1				
BAA196NC_2529	NA	*ycgL*						1		Hypothetical protein
BAA196NC_2569	NA	*hflD*		-1				-1		Hypothetical protein
BAA196NC_2576	NA	*ycfP*				1		1		Probably esterase of a salvage cluster
BAA196NC_2580	NA	*potD*		1						Spermidine/putrescine ABC transporter periplasmic substrate-binding protein
BAA196NC_2591	NA	*ycfR*			1	1		1		Hypothetical protein
BAA196NC_2642	NA	*pyrC*	1		1					Dihydroorotase
BAA196NC_2644	NA	*yceP*			-1					Hypothetical protein
BAA196NC_2652	NA	*msyB*		1		1		1		Hypothetical protein
BAA196NC_2653	NA	*yceK*						1		Predicted lipoprotein
BAA196NC_2654	NA	*mdoH*			1					Glucosyltransferase MdoH

BAA196NC_2687	NA	*putA*		1						Fused DNA-binding transcriptional regulator/proline dehydrogenase/pyrroline-5-carboxylate dehydrogenase
BAA196NC_2725	NA	*hyaD*		-1				-1		Protein involved in processing of HyaA and HyaB proteins
BAA196NC_2727	NA	*hyaB*						-1		Hydrogenase 1, large subunit
BAA196NC_2728	NA	*hyaA*						-1		Hydrogenase 1, small subunit
BAA196NC_2746	NA	*fabA*					1			3-hydroxydecanoyl-ACP dehydratase
BAA196NC_2747	NA	*rmf*		-1						Ribosome modulation factor
BAA196NC_2748	NA	*ymbA*					1			Hypothetical protein
BAA196NC_2751	NA	*uup*						-1		Fused predicted transporter subunits of ABC superfamily: ATP-binding components
BAA196NC_2753	NA	*ycbX*						-1		Predicted 2Fe-2S cluster-containing protein
BAA196NC_2771	NA	*ompF*	-1	-1	-1					Outer membrane porin 1a (Ia;b;F)
BAA196NC_2772	NA	*aspC*					1			Aspartate aminotransferase, PLP-dependent
BAA196NC_2794	NA	*ycaP*						1		Conserved inner membrane protein
BAA196NC_2802	NA	*ycaD*						1		Putative MFS family transporter protein
BAA196NC_2803	NA	*ycaC*						1		Predicted hydrolase
BAA196NC_2804	NA	*dmsC*						-1		Dimethyl sulfoxide reductase, anaerobic, subunit C
BAA196NC_2806	NA	*dmsA*					-1			Dimethyl sulfoxide reductase, anaerobic, subunit A
BAA196NC_2811	NA	*lrp*			-1					DNA-binding transcriptional dual regulator, leucine-binding
BAA196NC_2819	NA	*cspD*		-1						Cold shock protein homolog
BAA196NC_2829	NA	*ltaE*			1	1				L-allo-threonine aldolase, PLP-dependent
BAA196NC_2833	NA	*ybjQ*						1		Hypothetical protein
BAA196NC_2863	NA	*dacC*					-1			D-alanyl-D-alanine carboxypeptidase (penicillin-binding protein 6a)
BAA196NC_2865	NA	*yliI*				1				Predicted dehydrogenase
BAA196NC_2866	NA	*yliH*			-1		-1	1		Hypothetical protein
BAA196NC_2883	NA	*ybiS*					1			L,D-transpeptidase ldtB
BAA196NC_2886	NA	*rhlE*		-1		-1				RNA helicase
BAA196NC_2888	NA	*ompX*			-1					Outer membrane protein X
BAA196NC_2891	NA	*glnH*					-1			Glutamine ABC transporter periplasmic protein
BAA196NC_2892	NA	*glnP*			1					Glutamine ABC transporter permease protein
BAA196NC_2893	NA	*glnQ*			1		-1			Glutamine ABC transporter ATP-binding protein
BAA196NC_2896	NA	*ybiM*						1		Hypothetical protein
BAA196NC_2897	NA	*fiu*				1				Predicted iron outer membrane transporter
BAA196NC_2900	NA	*ybiJ*				1		1		Hypothetical protein
BAA196NC_2907	NA	*ybhG*						-1		Hypothetical protein
BAA196NC_2911	NA	*ybhQ*						1		Predicted inner membrane protein
BAA196NC_2938	NA	*ynfO-1*						1		Qin prophage protein
BAA196NC_2947	NA	*ninH*						-1		Uncharacterized phage protein
BAA196NC_2951	NA	*ninB*						-1		Phage DNA recombination
BAA196NC_2975	NA	*ybhI*		-1						Inner membrane protein
BAA196NC_3007	NA	*galK*					-1			Galactokinase
BAA196NC_3013	NA	*pnuC*						1		Predicted nicotinamide mononucleotide transporter
BAA196NC_3015	NA	*ybgF*			-1					Hypothetical protein
BAA196NC_3018	NA	*tolA*					1			Cell envelope integrity inner membrane protein TolA
BAA196NC_3024	NA	*cydB*					-1			Cytochrome d terminal oxidase, subunit II
BAA196NC_3036	NA	*sdhD*					-1	-1		Succinate dehydrogenase cytochrome b556 small membrane subunit
BAA196NC_3038	NA	*gltA*		1						Citrate synthase
BAA196NC_3058	NA	*ybfA*			-1		-1			Hypothetical protein
BAA196NC_3060	NA	*kdpA*						-1		Potassium-transporting ATPase subunit A
BAA196NC_3063	NA	*kdpD*						-1		Fused sensory histidine kinase in two-component regulatory system with KdpE: signal sensing protein
BAA196NC_3072	NA	*seqA*					1			Regulatory protein for replication initiation
BAA196NC_3075	NA	*fldA*			1		1			Flavodoxin 1
BAA196NC_3093	NA	*hscC*						-1		Hsp70 family chaperone Hsc62, binds to RpoD and inhibits transcription
BAA196NC_3117	NA	*dacA*					1	-1		D-alanyl-D-alanine carboxypeptidase (penicillin-binding protein 5)
BAA196NC_3118	NA	*ybeD*		-1						Hypothetical protein
BAA196NC_3122	NA	*tatE*		-1		-1				Twin arginine translocase protein E
BAA196NC_3128	NA	*dcuC*					-1			Anaerobic C4-dicarboxylate transport
BAA196NC_3142	NA	*uspG*				1		1		Universal stress protein UP12
BAA196NC_3144	NA	*ahpC*	1			1		1		Alkyl hydroperoxide reductase, C22 subunit
BAA196NC_3152	NA	*cstA*		1						Carbon starvation protein
BAA196NC_3155	NA	*entB*		1						Isochorismatase
BAA196NC_3189	NA	*appY*						1		DLP12 prophage; DNA-binding transcriptional activator
BAA196NC_3248	NA	*allR*			1			1		DNA-binding transcriptional repressor
BAA196NC_3293	NA	*ybaJ*						1		Hypothetical protein
BAA196NC_3296	NA	*ylaC*				-1				Predicted inner membrane protein
BAA196NC_3297	NA	*ylaB*		-1						Conserved inner membrane protein
BAA196NC_3299	NA	*ybaX*	1					-1		7-cyano-7-deazaguanine synthase
BAA196NC_3313	NA	*ppiD*					1			Peptidyl-prolyl cis-trans isomerase (rotamase D)
BAA196NC_3318	NA	*tig*	-1		-1	-1		-1		Trigger factor
BAA196NC_3320	NA	*bolA*		-1						Regulator of penicillin binding proteins and beta lactamase transcription (morphogene)
BAA196NC_3323	NA	*cyoA*	1				-1			Cytochrome o ubiquinol oxidase subunit II
BAA196NC_3325	NA	*cyoC*					-1			Cytochrome o ubiquinol oxidase subunit III
BAA196NC_3326	NA	*cyoD*					-1			Cytochrome o ubiquinol oxidase subunit IV
BAA196NC_3329	NA	*yajQ*						1		Nucleotide-binding protein
BAA196NC_3336	NA	*yajO*			-1					Predicted oxidoreductase, NAD(P)-binding
BAA196NC_3337	NA	*pgpA*					-1			Phosphatidylglycerophosphatase A
BAA196NC_3339	NA	*nusB*					1			Transcription antitermination protein NusB

BAA196NC_3355	NA	*phoR*			1					Sensory histidine kinase in two-component regulatory system with PhoB
BAA196NC_3361	NA	*rdgC*					1			Recombination associated protein
BAA196NC_3365	NA	*yaiA*		-1		-1				Hypothetical protein
BAA196NC_3370	NA	*psiF*		-1						Hypothetical protein
BAA196NC_3371	NA	*phoA*						1		Bacterial alkaline phosphatase
BAA196NC_3372	NA	*yaiB*						1		Hypothetical protein
BAA196NC_3400	NA	*frmA*		1	1	1		1		Alcohol dehydrogenase class III/glutathione-dependent formaldehyde dehydrogenase
BAA196NC_3419	NA	*codA*	1		1		-1			Cytosine deaminase
BAA196NC_3420	NA	*codB*			1					Cytosine transporter
BAA196NC_3424	NA	*prpB*					1			2-methylisocitrate lyase
BAA196NC_3426	NA	*yahO*		-1						Hypothetical protein
BAA196NC_3452	NA	*ykgC*						1		Pyridine nucleotide-disulfide oxidoreductase
BAA196NC_3460	NA	*ykgO*						1		RpmJ (L36) paralog
BAA196NC_3472	NA	*yagR*		-1				-1		Predicted oxidoreductase with molybdenum-binding domain
BAA196NC_3515	NA	*dinJ*		-1						Predicted antitoxin of YafQ-DinJ toxin-antitoxin system
BAA196NC_3521	NA	*ivy*				1				Inhibitor of vertebrate C-lysozyme
BAA196NC_3526	NA	*dnaQ*					1	1		DNA polymerase III subunit epsilon
BAA196NC_3530	NA	*mltD*	-1		-1		1			Predicted membrane-bound lytic murein transglycosylase D
BAA196NC_3546	NA	*yaeP*						1		Hypothetical protein
BAA196NC_3547	NA	*rof*						1		Modulator of Rho-dependent transcription termination
BAA196NC_3550	NA	*ldcC*					-1			Lysine decarboxylase 2, constitutive
BAA196NC_3551	NA	*accA*			-1					Acetyl-CoA carboxylase subunit alpha
BAA196NC_3555	NA	*lpxA*				1				UDP-N-acetylglucosamine acyltransferase
BAA196NC_3558	NA	*hlpA*				-1				Periplasmic chaperone
BAA196NC_3577	NA	*degP*		1	1					Serine endoprotease (protease Do), membrane-associated
BAA196NC_3579	NA	.			-1					5'-methylthioadenosine/S-adenosylhomocysteine nucleosidase
BAA196NC_3583	NA	*clcA*		-1						Chloride channel protein
BAA196NC_3588	NA	*fhuA*				1				Ferrichrome outer membrane transporter
BAA196NC_3592	NA	*sfsA*	-1	-1						Sugar fermentation stimulation protein A
BAA196NC_3593	NA	*dksA*	-1		-1					DNA-binding transcriptional regulator of rRNA transcription, DnaK suppressor protein
BAA196NC_3615	NA	*cueO*	1	1	1	1				Multicopper oxidase (laccase)
BAA196NC_3620	NA	*acnB*		1						Aconitate hydratase
BAA196NC_3623	NA	*aceF*		1				-1		Dihydrolipoamide acetyltransferase
BAA196NC_3645	NA	*murC*					1	-1		UDP-N-acetylmuramate--L-alanine ligase
BAA196NC_3652	NA	*ftsI*				1		-1		Transpeptidase involved in septal peptidoglycan synthesis (penicillin-binding protein 3)
BAA196NC_3682	NA	*surA*						-1		Peptidyl-prolyl cis-trans isomerase (PPIase)
BAA196NC_3716	NA	*nhaA*						1		PH-dependent sodium/proton antiporter
BAA196NC_3721	NA	*dnaK*					-1			Molecular chaperone DnaK
BAA196NC_3734	NA	*arcA*					1			DNA-binding response regulator in two-component regulatory system with ArcB or CpxA
BAA196NC_3761	NA	*osmY*		1		1		1		Periplasmic protein
BAA196NC_3773	NA	*dnaT*				-1				Primosomal protein I
BAA196NC_3781	NA	*yjiY*		1		1	-1			Predicted inner membrane protein
BAA196NC_3785	NA	*hsdR*						-1		Endonuclease R
BAA196NC_3824	NA	*fimA*				1				Major type 1 subunit fimbrin (pilin)
BAA196NC_3878	NA	*pepA*			1					Leucyl aminopeptidase
BAA196NC_3879	NA	*holC*		1						DNA polymerase III subunit chi
BAA196NC_3883	NA	*yjgD*				-1				Hypothetical protein
BAA196NC_3889	NA	*yjgI*		1		1		1		Predicted oxidoreductase with NAD(P)-binding Rossmann-fold domain
BAA196NC_3892	NA	*pyrI*	1		1					Aspartate carbamoyltransferase regulatory subunit
BAA196NC_3894	NA	*mgtA*				1				Magnesium transporter
BAA196NC_3901	NA	*pmbA*				-1				Predicted peptidase required for the maturation and secretion of the antibiotic peptide MccB17
BAA196NC_3908	NA	*ytfQ*						1		Predicted sugar transporter subunit: periplasmic-binding component of ABC superfamily
BAA196NC_3909	NA	*ppa*					1			Inorganic pyrophosphatase
BAA196NC_3933	NA	*priB*		1						Primosomal replication protein N
BAA196NC_3945	NA	*yjfO*	-1	-1	-1		-1			Hypothetical protein
BAA196NC_3946	NA	*yjfN*	-1		-1		-1			Hypothetical protein
BAA196NC_3962	NA	*hfq*			-1					RNA-binding protein Hfq
BAA196NC_3982	NA	*blc*						1		Outer membrane lipoprotein (lipocalin)
BAA196NC_3984	NA	*ecnB*		-1		-1				Entericidin B membrane lipoprotein
BAA196NC_3994	NA	*aspA*	-1		-1		-1			Aspartate ammonia-lyase
BAA196NC_3995	NA	*dcuA*				-1	-1	-1		C4-dicarboxylate antiporter
BAA196NC_4070	NA	*yjcD*	1		1					Predicted permease
BAA196NC_4072	NA	*yjbQ*		-1						Uncharacterized protein
BAA196NC_4075	NA	*yjcC*		-1						Predicted signal transduction protein (EAL domain containing protein)
BAA196NC_4079	NA	*yjbR*		-1		-1				Hypothetical protein
BAA196NC_4087	NA	*qor*				1				Quinone oxidoreductase, NADPH-dependent
BAA196NC_4092	NA	*zur*		-1						DNA-binding transcriptional repressor, Zn(II)-binding
BAA196NC_4093	NA	*yjbJ*		-1				1		Predicted stress response protein
BAA196NC_4102	NA	*lamB*						-1		Maltoporin precursor
BAA196NC_4103	NA	*malK*						-1		Fused maltose transport subunit, ATP-binding component of ABC superfamily/regulatory protein

BAA196NC_4119	NA	*metH*						-1							B12-dependent methionine synthase
BAA196NC_4122	NA	*aceK*				1		-1							Bifunctional isocitrate dehydrogenase kinase/phosphatase protein
BAA196NC_4123	NA	*aceA*		1		1									Isocitrate lyase
BAA196NC_4124	NA	*aceB*		1		1		-1							Malate synthase
BAA196NC_4136	NA	*nfi*						-1							Endonuclease V
BAA196NC_4140	NA	*thiC*					-1								Thiamine biosynthesis protein ThiC
BAA196NC_4142	NA	*thiF*					-1								Thiamine biosynthesis protein ThiF
BAA196NC_4145	NA	*thiH*					-1								Thiamine biosynthesis protein ThiH
BAA196NC_4164	NA	*fabR*				1									DNA-binding transcriptional repressor
BAA196NC_4189	NA	*metJ*			-1										Transcriptional repressor protein MetJ
BAA196NC_4191	NA	*rpmE*	-1	-1	-1	-1									50S ribosomal subunit protein L31
BAA196NC_4194	NA	*ftsN*					1								Essential cell division protein
BAA196NC_4198	NA	*rraA*		-1											Ribonuclease activity regulator protein RraA
BAA196NC_4204	NA	*yiiT*						1							Stress-induced protein
BAA196NC_4205	NA	*yiiS*				1		1							Hypothetical protein
BAA196NC_4210	NA	*sbp*			1	1		1							Sulfate transporter subunit
BAA196NC_4213	NA	*cpxP*				1									Periplasmic protein combats stress
BAA196NC_4234	NA	*fdoG*		1	1			-1							Formate dehydrogenase-O, large subunit
BAA196NC_4235	NA	*fdoH*					-1								Formate dehydrogenase-O, Fe-S subunit
BAA196NC_4236	NA	*fdoI*		1											Formate dehydrogenase-O, cytochrome b556 subunit
BAA196NC_4237	NA	*fdhE*						-1							Formate dehydrogenase accessory protein FdhE
BAA196NC_4260	NA	*glnL*			1										Sensory histidine kinase in two-component regulatory system with GlnG
BAA196NC_4261	NA	*glnG*					-1								Fused DNA-binding response regulator in two-component regulatory system with GlnL: response regulator/sigma54 interaction protein
BAA196NC_4272	NA	*yihE*				1									Predicted kinase
BAA196NC_4273	NA	*yihD*				-1									Hypothetical protein
BAA196NC_4285	NA	*tatD*						-1							DNase, magnesium-dependent
BAA196NC_4287	NA	*tatB*						-1							Sec-independent translocase
BAA196NC_4316	NA	*cyaY*						-1							Frataxin-like protein
BAA196NC_4317	NA	*cyaA*		-1		-1									Adenylate cyclase
BAA196NC_4322	NA	*hemC*		-1			-1								Porphobilinogen deaminase
BAA196NC_4336	NA	*wzzE*				-1									Entobacterial Common Antigen (ECA) polysaccharide chain length modulation protein
BAA196NC_4341	NA	*rhlB*		-1											ATP-dependent RNA helicase
BAA196NC_4342	NA	*gpp*				1									Guanosine pentaphosphatase/exopolyphosphatase
BAA196NC_4367	NA	*asnA*					1								Asparagine synthetase AsnA
BAA196NC_4383	NA	*pstS*	1		1			1							Phosphate transporter subunit
BAA196NC_4404	NA	*tnaA*			-1		-1								Tryptophanase/L-cysteine desulfhydrase, PLP-dependent
BAA196NC_4428	NA	*aph3*			-1										Aminoglycoside 3'-phosphotransferase
BAA196NC_4442	NA	*aacA6*	-1		-1										Aminoglycoside N(6')-acetyltransferase
BAA196NC_4447	NA	.						-1							S-(hydroxymethyl)glutathione dehydrogenase
BAA196NC_4471	NA	*terD*						1							Tellurium resistance protein TerD
BAA196NC_4472	NA	*terE*						1							Tellurium resistance cAMP binding protein TerE
NA	HMPRNC0000_0008	*hutH*							-1		-1				Histidine ammonia-lyase
NA	HMPRNC0000_0014	.											-1		Hypothetical protein
NA	HMPRNC0000_0015	.								1		1			Cyclic-di-AMP phosphodiesterase GdpP
NA	HMPRNC0000_0023	*yycH*								1		1			Two-component system YycFG regulatory protein YycH
NA	HMPRNC0000_0026	.							1						Zn-dependent hydrolase YycJ/WalJ
NA	HMPRNC0000_0027	*sasH*									-1				Virulence-associated cell-wall-anchored protein SasH (LPXTG motif), 5'-nucleotidase
NA	HMPRNC0000_0034	*cadC*							1		1				Cadmium efflux system accessory protein
NA	HMPRNC0000_0037	.							1	1	1	1			Cadmium resistance protein
NA	HMPRNC0000_0038	.							1	1	1	1			DNA repair protein RadC
NA	HMPRNC0000_0045	*mecA*							-1		-1				Penicillin-binding protein PBP2a, methicillin resistance determinant MecA, transpeptidase
NA	HMPRNC0000_0052	.							1						ctRNA
NA	HMPRNC0000_0053	.							1						Uncharacterized ncRNA
NA	HMPRNC0000_0054	*pre*							1		1				Plasmid recombination enzyme type 3
NA	HMPRNC0000_0055	*tet*								1		1			Tetracycline resistance, MFS efflux pump -> Tet(K)
NA	HMPRNC0000_0102	.							-1	-1	-1	-1		1	Protein A, von Willebrand factor binding protein Spa
NA	HMPRNC0000_0121	.									1				Hypothetical protein
NA	HMPRNC0000_0123	*budC*							1	-1		-1		1	2,3-butanediol dehydrogenase, S-alcohol forming, (R)-acetoin-specific / Acetoin (diacetyl) reductase
NA	HMPRNC0000_0129	*sodA*							1						Superoxide dismutase (Mn), Superoxide dismutase (Fe)
NA	HMPRNC0000_0173	.									1				Hypothetical protein
NA	HMPRNC0000_0176	.							1	1	1	1			Hypothetical protein
NA	HMPRNC0000_0178	.							1		1				Acyl-CoA dehydrogenase, short-chain specific
NA	HMPRNC0000_0182	.							-1		-1				Polyketide synthase
NA	HMPRNC0000_0194	*ipdC*							-1		-1				Pyruvate decarboxylase, Alpha-keto-acid decarboxylase
NA	HMPRNC0000_0207	*hsdR*							-1						Type I restriction-modification system, restriction subunit R
NA	HMPRNC0000_0234	.							-1		-1				Inosose isomerase
NA	HMPRNC0000_0246	.							1						Hypothetical protein
NA	HMPRNC0000_0248	*fadA*								-1		-1	1		3-ketoacyl-CoA thiolase (fadN-fadA-fadE operon)
NA	HMPRNC0000_0249	*fadB*								-1		-1			3-hydroxyacyl-CoA dehydrogenase/Enoyl-CoA hydratase
NA	HMPRNC0000_0261	*ldhA*												-1	L-lactate dehydrogenase
NA	HMPRNC0000_0284	*lrgB*								1		1	-1		LrgA-associated membrane protein LrgB
NA	HMPRNC0000_0299	.							-1						Hypothetical protein
NA	HMPRNC0000_0301	.							-1		-1				Hypothetical protein
NA	HMPRNC0000_0307	*esxA*							-1	-1		-1			6 kDa early secretory antigenic target ESAT-6 (EsxA)

NA	HMPRNC0000_0312	*essC*		-1						FtsK/SpoIIIE family protein, putative EssC/YukB component of Type VII secretion system
NA	HMPRNC0000_0334	*brnQ*				1				Branched-chain amino acid transport system carrier protein
NA	HMPRNC0000_0337	*lolD*			-1		-1			ABC transporter, ATP-binding protein
NA	HMPRNC0000_0338	*pfoR*				-1				Perfringolysin O regulator protein PfoR
NA	HMPRNC0000_0347	.		-1						N-acetylmannosamine kinase
NA	HMPRNC0000_0348	.				1				Sialic acid utilization regulator, RpiR family
NA	HMPRNC0000_0351	*lip2*			-1	-1	-1			Lipase precursor
NA	HMPRNC0000_0363	.		-1						Putative sugar-specific permease, SgaT/UlaA
NA	HMPRNC0000_0364	.		-1		-1				PTS system, IIB component
NA	HMPRNC0000_0370	*glpT*		-1		-1				Glycerol-3-phosphate transporter
NA	HMPRNC0000_0371	*gloA*		1						Glyoxalase/Bleomycin resistance protein/Dioxygenase superfamily
NA	HMPRNC0000_0372	.		1						Luciferase-like monooxygenase YhbW
NA	HMPRNC0000_0373	.		1	1		1			FMN reductase
NA	HMPRNC0000_0388	.						1		Transcriptional regulator, Xre family
NA	HMPRNC0000_0417	.			-1		-1			hypothetical protein
NA	HMPRNC0000_0418	.				1				Hypothetical protein
NA	HMPRNC0000_0419	.			-1		-1		-1	Hypothetical protein
NA	HMPRNC0000_0427	.		1		1				L-cystine uptake protein TcyP, sodium:anion symporter family
NA	HMPRNC0000_0431	.			-1		-1			hypothetical protein
NA	HMPRNC0000_0467	.						-1	-1	Putative transmembrane protein coupled to NADH-ubiquinone oxidoreductase chain 5 protein
NA	HMPRNC0000_0471	.		1		1				PAP2 family phosphatase/haloperoxidase
NA	HMPRNC0000_0472	.		1						Esterase/lipase family protein
NA	HMPRNC0000_0474	.		1		1				Sodium-dependent transporter
NA	HMPRNC0000_0480	*sle*						-1		Autolysin precursor
NA	HMPRNC0000_0482	.		1						Hypothetical protein
NA	HMPRNC0000_0483	.		1						Acetyltransferase, GNAT family
NA	HMPRNC0000_0490	*treP*			1		1	-1		PTS system, trehalose-specific IIB component / PTS system, trehalose-specific IIC component
NA	HMPRNC0000_0492	*treR*			1		1	-1		Trehalose operon transcriptional repressor
NA	HMPRNC0000_0493	.		-1	-1		-1			Bacterial signal recognition particle RNA
NA	HMPRNC0000_0495	*dnaX*			1		1			DNA polymerase III subunits gamma and tau
NA	HMPRNC0000_0504	.			-1		-1			nitrogen regulatory protein P-II (GLNB)
NA	HMPRNC0000_0516	.			-1		-1	-1	-1	Veg protein
NA	HMPRNC0000_0518	*purR*		1	1		1			Pur operon repressor PurR
NA	HMPRNC0000_0530	.		1						Ribosome-associated heat shock protein implicated in the recycling of the 50S subunit (S4 protein)
NA	HMPRNC0000_0531	.				1				Cell division protein DivIC (FtsB), stabilizes FtsL against RasP cleavage
NA	HMPRNC0000_0532	.						-1		RNA binding protein, contains ribosomal protein S1 domain
NA	HMPRNC0000_0539	*cysK*			1		1			Cysteine synthase
NA	HMPRNC0000_0561	.		-1		-1				Pyridoxal 5'-phosphate synthase (glutamine hydrolyzing), synthase subunit
NA	HMPRNC0000_0562	*pdxT*		-1		-1				Pyridoxal 5'-phosphate synthase (glutamine hydrolyzing), glutaminase subunit
NA	HMPRNC0000_0563	.		-1						Pyrimidine nucleoside transporter NupC/NucG family
NA	HMPRNC0000_0564	.		-1		-1		-1		Pyrimidine nucleoside transporter NupC/NucG family
NA	HMPRNC0000_0565	*ctsR*		1	1	1	1			Transcriptional regulator CtsR
NA	HMPRNC0000_0566	.		1	1	1	1		1	Nucleotide excision repair protein, with UvrB/UvrC motif
NA	HMPRNC0000_0567	.		1	1	1	1		1	Arginine kinase
NA	HMPRNC0000_0568	*clpC*		1	1	1	1		1	ATP-dependent Clp protease, ATP-binding subunit ClpC
NA	HMPRNC0000_0572	*cysE*		1						Serine acetyltransferase
NA	HMPRNC0000_0574	.		1						Mini-ribonuclease III
NA	HMPRNC0000_0576	*yacP*		1		1				Uncharacterized protein
NA	HMPRNC0000_0584	.						-1		16S rRNA (guanine(1207)-N(2))-methyltransferase
NA	HMPRNC0000_0587	*rplL3*							-1	Firmicutes ribosomal L7Ae family protein
NA	HMPRNC0000_0592	.		1						N-acetyl-L,L-diaminopimelate deacetylase
NA	HMPRNC0000_0597	*tdh*		-1		-1				L-threonine 3-dehydrogenase
NA	HMPRNC0000_0606	*sdrC*						-1		Adhesin SdrC
NA	HMPRNC0000_0608	*sdrD*		-1						Adhesin SdrD
NA	HMPRNC0000_0609	*sdrE*						-1		Adhesin SdrE
NA	HMPRNC0000_0613	.		1						GTP cyclohydrolase I type 2
NA	HMPRNC0000_0614	.		1		1				GTP cyclohydrolase I type 2
NA	HMPRNC0000_0615	*bshB2*		1						N-acetylglucosaminyl-L-malate N-acetyl hydrolase
NA	HMPRNC0000_0616	*yojF*				1				Uncharacterized protein
NA	HMPRNC0000_0625	*vraB*				-1				3-ketoacyl-CoA thiolase and Acetyl-CoA acetyltransferase
NA	HMPRNC0000_0638	.			1		1			Octanoyl-(GcvH):protein N-octanoyltransferase
NA	HMPRNC0000_0643	.		1						DUF1450 superfamily protein
NA	HMPRNC0000_0644	*merA2*		1	1	1	1			Putative Dihydrolipoamide dehydrogenase/Mercuric ion reductase
NA	HMPRNC0000_0645	.		1	1	1	1			Hypothetical protein
NA	HMPRNC0000_0659	.			1		1			Hypothetical protein
NA	HMPRNC0000_0662	.		1	1		1			dNTP triphosphohydrolase, broad substrate specificity
NA	HMPRNC0000_0673	.		-1	-1		-1			Hypothetical protein
NA	HMPRNC0000_0689	.		1	1	1	1			Na+/H+ antiporter
NA	HMPRNC0000_0690	*sitC*		-1		-1				Manganese ABC transporter, periplasmic-binding protein SitA
NA	HMPRNC0000_0692	.		-1		-1		-1	-1	Manganese ABC transporter, ATP-binding protein SitB
NA	HMPRNC0000_0693	*sirR*						-1		Mn-dependent transcriptional regulator MntR
NA	HMPRNC0000_0704	*lmrA*				1				Efflux ABC transporter, permease/ATP-binding protein
NA	HMPRNC0000_0712	*fhuG*				1				Ferrichrome transport system permease protein FhuG

NA	HMPRNC0000_0716	.						1		Phosphoenolpyruvate-dihydroxyacetone phosphotransferase, subunit DhaM, DHA-specific IIA component
NA	HMPRNC0000_0717	.			1		1			Hypothetical protein
NA	HMPRNC0000_0718	.			1		1			Hypothetical protein
NA	HMPRNC0000_0723	.		1						Hypothetical protein
NA	HMPRNC0000_0729	.			1		1			Putative low-affinity inorganic phosphate transporter
NA	HMPRNC0000_0730	*amiD7*		-1	-1		-1			Secretory antigen precursor SsaA
NA	HMPRNC0000_0738	.		1						Hypothetical protein
NA	HMPRNC0000_0739	.		1	1		1			Catabolite control protein E (CcpE), LysR family
NA	HMPRNC0000_0750	.			-1		-1			hypothetical protein
NA	HMPRNC0000_0751	*uppP*		-1		-1				Undecaprenyl-diphosphatase
NA	HMPRNC0000_0754	*mgrA*		-1						Transcriptional regulator MgrA (Regulator of autolytic activity)
NA	HMPRNC0000_0771	*pfkB2*		1	1	1	1			1-phosphofructokinase
NA	HMPRNC0000_0772	*fruA*		1	1	1	1			PTS system, fructose-specific IIA/IIB/IIC component
NA	HMPRNC0000_0778	*saeR*			-1		-1			Response regulator SaeR, exoprotein expression protein R
NA	HMPRNC0000_0779	.			-1		-1	1		Hypothetical protein
NA	HMPRNC0000_0780	.						1		Hypothetical protein
NA	HMPRNC0000_0829	.		1						Protein co-occurring with transport systems
NA	HMPRNC0000_0837	*secA*			1		1			Protein translocase subunit SecA
NA	HMPRNC0000_0839	.				1				LysM domain protein
NA	HMPRNC0000_0859	*cggR*			1		1	-1	-1	Central glycolytic regulator
NA	HMPRNC0000_0860	*gapA1*		1					-1	NAD-dependent glyceraldehyde-3-phosphate dehydrogenase
NA	HMPRNC0000_0877	*clfA*			-1		-1		-1	Clumping factor ClfA, fibrinogen-binding protein
NA	HMPRNC0000_0883	*cspA2*		-1				-1		Cold shock protein of CSP family
NA	HMPRNC0000_0898	.				1				Putative nitroreductase family protein
NA	HMPRNC0000_0903	.		-1						Glutathione-dependent thiol reductase
NA	HMPRNC0000_0909	.		-1						Uncharacterized ncRNA
NA	HMPRNC0000_0910	*metN2*		1		1				Methionine ABC transporter ATP-binding protein
NA	HMPRNC0000_0911	.		1		1				Methionine ABC transporter permease protein
NA	HMPRNC0000_0912	*metQ*				1				Methionine ABC transporter substrate-binding protein
NA	HMPRNC0000_0916	*sei*		-1						Exotoxin, phage associated
NA	HMPRNC0000_0924	.				1				Hypothetical protein
NA	HMPRNC0000_0931	.		1						Putative terminase, superantigen-encoding pathogenicity islands SaPI
NA	HMPRNC0000_0942	.			-1		-1			hemolysin
NA	HMPRNC0000_0947	.						-1		UPF0721 transmembrane protein YunE
NA	HMPRNC0000_0948	.			1		1			5'-nucleotidase family protein
NA	HMPRNC0000_0958	*dltB*						1		D-alanyl transfer protein DltB
NA	HMPRNC0000_0960	*dltD*						1		Poly(glycerophosphate chain) D-alanine transfer protein DltD
NA	HMPRNC0000_0964	.		1						DUF1450 superfamily protein
NA	HMPRNC0000_0969	.		1	1	1	1			Histidine permease YuiF
NA	HMPRNC0000_0978	*mnhD*			1		1			Na(+) H(+) antiporter subunit D
NA	HMPRNC0000_0981	*mnhA*						-1		Na(+) H(+) antiporter subunit A
NA	HMPRNC0000_0985	.						-1		General stress protein 13
NA	HMPRNC0000_0991	*argG*		1		1				Argininosuccinate synthase
NA	HMPRNC0000_0997	*addB*		1		1				ATP-dependent helicase/nuclease AddAB, subunit B
NA	HMPRNC0000_1001	*fahA*			-1		-1			Fumarylacetoacetate hydrolase family protein
NA	HMPRNC0000_1005	*yitU*				1				Haloacid dehalogenase hydrolase
NA	HMPRNC0000_1010	.		1		1				Transcriptional regulator, LysR family
NA	HMPRNC0000_1013	.						1		Hypothetical protein
NA	HMPRNC0000_1014	.						1		Hypothetical protein
NA	HMPRNC0000_1031	*mecA2*		1		1				Negative regulator of genetic competence, sporulation and motility
NA	HMPRNC0000_1033	*pepF*		-1						Oligoendopeptidase F
NA	HMPRNC0000_1040	*ppnK*							1	NAD kinase
NA	HMPRNC0000_1041	*yjbO*				1				Ribosomal large subunit pseudouridine synthase D
NA	HMPRNC0000_1050	.		-1	-1	-1	-1			2H phosphoesterase superfamily protein Bsu1186 (yjcG)
NA	HMPRNC0000_1063	.			-1		-1		1	Hypothetical protein
NA	HMPRNC0000_1086	*atl*		-1	-1		-1			N-acetylmuramoyl-L-alanine amidase / Endo-beta-N-acetylglucosaminidase and Bifunctional autolysin Atl
NA	HMPRNC0000_1089	.						-1		Cell envelope-associated transcriptional attenuator LytR-CpsA-Psr, subfamily F2
NA	HMPRNC0000_1093	*qoxD*		-1	-1		-1			Cytochrome aa3-600 menaquinol oxidase subunit IV
NA	HMPRNC0000_1095	*qoxA*							-1	Cytochrome aa3-600 menaquinol oxidase subunit II
NA	HMPRNC0000_1098	*folD*		-1	-1	-1	-1			Methenyltetrahydrofolate cyclohydrolase / Methylenetetrahydrofolate dehydrogenase (NADP+)
NA	HMPRNC0000_1103	*purS*				-1				Phosphoribosylformylglycinamidine synthase, PurS subunit
NA	HMPRNC0000_1104	*purQ*		-1		-1				Phosphoribosylformylglycinamidine synthase, glutamine amidotransferase subunit
NA	HMPRNC0000_1105	*purL*		-1		-1			-1	Phosphoribosylformylglycinamidine synthase, synthetase subunit
NA	HMPRNC0000_1113	.		-1		-1				Duplicated ATPase component YkoD
NA	HMPRNC0000_1114	.		-1	-1	-1	-1			Substrate-specific component YkoE of thiamin-regulated ECF transporter for HydroxyMethylPyrimidine
NA	HMPRNC0000_1126	*ktrA*		1						KtrCD potassium uptake system, peripheral membrane component KtrC
NA	HMPRNC0000_1127	.			1		1			Ribonuclease J1 (endonuclease and 5' exonuclease)
NA	HMPRNC0000_1145	.		1						Hypothetical protein
NA	HMPRNC0000_1149	.						1		Hypothetical protein
NA	HMPRNC0000_1153	.		1				1		UPF0358 protein YlaN
NA	HMPRNC0000_1191	.		1		1				DNA polymerase X family
NA	HMPRNC0000_1192	*mutS3*			1		1			Recombination inhibitory protein MutS2

NA	HMPRNC0000_1195	*sdhC*	-1						Succinate dehydrogenase cytochrome b558 subunit
NA	HMPRNC0000_1203	.	1		1				Hypothetical protein
NA	HMPRNC0000_1206	*fib*						1	Extracellular fibrinogen-binding protein Efb
NA	HMPRNC0000_1207	*scn*	1						Fibrinogen-binding prophage protein
NA	HMPRNC0000_1210	*hly*						1	Cytolytic pore-forming protein -> Alpha-hemolysin
NA	HMPRNC0000_1227	*bshC*	1	1	1	1			Glucosaminyl-malate:cysteine ligase
NA	HMPRNC0000_1230	*pbpI*	-1						Cell division protein FtsI (Peptidoglycan synthetase) and Penicillin-binding protein 2B
NA	HMPRNC0000_1233	*divIB*	-1		-1				Cell division protein FtsQ
NA	HMPRNC0000_1234	*ftsA*	-1		-1				Cell division protein FtsA
NA	HMPRNC0000_1243	.					-1		Cell division initiation protein DivIVA
NA	HMPRNC0000_1251	*upp2*	-1						Pyrimidine operon regulatory protein PyrR
NA	HMPRNC0000_1258	*pyrE*					1		Orotate phosphoribosyltransferase
NA	HMPRNC0000_1261	*fbpA*		1		1			Fibronectin/fibrinogen-binding protein
NA	HMPRNC0000_1269	.	-1						Hypothetical protein
NA	HMPRNC0000_1271	.		-1		-1			hypothetical protein
NA	HMPRNC0000_1277	*prkC*		1		1			Serine/threonine protein kinase PrkC, regulator of stationary phase
NA	HMPRNC0000_1281	*rpmB*	-1	-1		-1			LSU ribosomal protein L28p and LSU ribosomal protein L28p, zinc-independent
NA	HMPRNC0000_1283	.	-1						Dihydroxyacetone kinase
NA	HMPRNC0000_1287	.		1		1			Transcription factor FapR
NA	HMPRNC0000_1321	*tsf*		1		1			Translation elongation factor Ts
NA	HMPRNC0000_1328	*polC*		1		1			DNA polymerase III polC-type
NA	HMPRNC0000_1332	.	1						Putative nucleic-acid-binding protein implicated in transcription termination
NA	HMPRNC0000_1341	.		1		1			Ribonuclease J2 (endoribonuclease in RNA processing)
NA	HMPRNC0000_1347	.		1		1			ACT domain protein
NA	HMPRNC0000_1352	*cvfA*						1	Ribonuclease Y
NA	HMPRNC0000_1356	*porB*			1				2-oxoglutarate/2-oxoacid ferredoxin oxidoreductase, beta subunit
NA	HMPRNC0000_1368	*glpD*						1	Aerobic glycerol-3-phosphate dehydrogenase
NA	HMPRNC0000_1377	*glnR*	-1						FemC, factor involved in methicillin resistance / Glutamine synthetase repressor
NA	HMPRNC0000_1421	.	-1		-1				Hypothetical protein
NA	HMPRNC0000_1423	*thrA*	1		1			-1	Homoserine dehydrogenase
NA	HMPRNC0000_1424	*thrC*	1					-1	Threonine synthase
NA	HMPRNC0000_1438	.					-1		Hypothetical protein
NA	HMPRNC0000_1448	*opuD2*	-1	1	-1	1			Glycine betaine transporter OpuD
NA	HMPRNC0000_1463	*msrR*	-1		-1				Peptide methionine sulfoxide reductase regulator MsrR
NA	HMPRNC0000_1472	*trpF*		-1		-1			Phosphoribosylanthranilate isomerase
NA	HMPRNC0000_1483	*pepF*	-1						Oligoendopeptidase F
NA	HMPRNC0000_1512	.					-1		5-bromo-4-chloroindolyl phosphate hydrolysis protein XpaC
NA	HMPRNC0000_1514	*brnQ*	1						Branched-chain amino acid transport system carrier protein
NA	HMPRNC0000_1515	.	1	1		1			Nitric oxide reductase activation protein NorD
NA	HMPRNC0000_1541	.					-1		Surface anchored protein
NA	HMPRNC0000_1543	*ebh*		1		1	-1		Surface anchored protein
NA	HMPRNC0000_1550	.			-1				DNA polymerase I 5'-3' exonuclease domain
NA	HMPRNC0000_1552	.	-1						Bsub YpbR
NA	HMPRNC0000_1553	.	-1						Iron-regulated membrane protein, Iron-uptake factor PiuB
NA	HMPRNC0000_1554	.					-1		23S rRNA (guanine(2445)-N(2))-methyltransferase
NA	HMPRNC0000_1555	.	-1	-1	-1	-1			Bacterial RNase P class B
NA	HMPRNC0000_1559	*recU*	-1	1	-1	1			RecU Holliday junction resolvase
NA	HMPRNC0000_1560	*pbp*		1		1			Multimodular transpeptidase-transglycosylase / Penicillin-binding protein 1A/1B (PBP1)
NA	HMPRNC0000_1563	.	1						Hypothetical protein
NA	HMPRNC0000_1576	.	1						Hypothetical protein
NA	HMPRNC0000_1577	.			1				Hypothetical protein
NA	HMPRNC0000_1578	.			1				TPR-repeat-containing protein
NA	HMPRNC0000_1591	*rpsA*	-1						SSU ribosomal protein S1p
NA	HMPRNC0000_1597	*recQ*					-1		ATP-dependent DNA helicase RecS (RecQ family)
NA	HMPRNC0000_1608	*srrB*					-1		Respiratory response protein SrrB
NA	HMPRNC0000_1618	*nudF*	-1						ADP-ribose pyrophosphatase
NA	HMPRNC0000_1631	.					-1		Glyoxalase/bleomycin resistance protein/dioxygenase superfamily protein
NA	HMPRNC0000_1662	*gcvP2*	-1		-1				Glycine dehydrogenase (decarboxylating) (glycine cleavage system P2 protein)
NA	HMPRNC0000_1663	*gcvP*	-1		-1				Glycine dehydrogenase (decarboxylating) (glycine cleavage system P1 protein)
NA	HMPRNC0000_1679	*fthC*					1		5-formyltetrahydrofolate cyclo-ligase
NA	HMPRNC0000_1681	*pbp3*			-1				Conserved protein Pbp3
NA	HMPRNC0000_1691	*dnaG*		1		1			DNA primase
NA	HMPRNC0000_1694	*glyS*		-1		-1			Glycyl-tRNA synthetase
NA	HMPRNC0000_1704	.						-1	Hypothetical protein
NA	HMPRNC0000_1707	*prmA*	1	1	1	1			Ribosomal protein L11 methyltransferase
NA	HMPRNC0000_1710	*dnaK*	1	1	1	1		1	Chaperone protein DnaK
NA	HMPRNC0000_1713	*hrcA*	1	1	1	1			Heat-inducible transcription repressor HrcA
NA	HMPRNC0000_1735	.	-1						Metal ion transporter YcsG, Mn(2+)/Fe(2+) NRAMP family
NA	HMPRNC0000_1737	*accC*			-1				Biotin carboxylase
NA	HMPRNC0000_1745	.	1						Protease small subunit YrrN
NA	HMPRNC0000_1759	.					1		HesA/MoeB/ThiF family protein
NA	HMPRNC0000_1760	.	-1	-1		-1		1	6S / SsrS RNA

NA	HMPRNC0000_1772	*queA*							-1	S-adenosylmethionine:tRNA ribosyltransferase-isomerase
NA	HMPRNC0000_1777	*cgtA*		1	1		1			GTP-binding protein Obg
NA	HMPRNC0000_1790	.				1				3'UTR
NA	HMPRNC0000_1802	*clpX*			1		1			ATP-dependent Clp protease ATP-binding subunit ClpX
NA	HMPRNC0000_1804	*tig*			1		1		1	Cell division trigger factor
NA	HMPRNC0000_1805	.						-1		Hypothetical protein
NA	HMPRNC0000_1809	*infC*		1						Translation initiation factor 3
NA	HMPRNC0000_1812	*dnaI*			1		1			Helicase loader DnaI
NA	HMPRNC0000_1827	*cycA2*		-1				-1		D-serine/D-alanine/glycine transporter
NA	HMPRNC0000_1834	*maeB*		-1						NADP-dependent malic enzyme
NA	HMPRNC0000_1836	.						-1		3'-to-5' oligoribonuclease A
NA	HMPRNC0000_1839	.		-1	-1		-1	1	1	Universal stress protein family
NA	HMPRNC0000_1842	.		-1		-1				Transcript
NA	HMPRNC0000_1843	*ald*		-1		-1		1		Alanine dehydrogenase
NA	HMPRNC0000_1845	.			-1		-1		1	Universal stress protein family
NA	HMPRNC0000_1858	.				-1		1		Transcript
NA	HMPRNC0000_1863	.				1				Phosphoserine aminotransferase, putative
NA	HMPRNC0000_1864	*serA*		1		1				D-3-phosphoglycerate dehydrogenase
NA	HMPRNC0000_1868	.				-1			1	Serine protease, DegP/HtrA
NA	HMPRNC0000_1875	*fhs*		-1		-1				Formate--tetrahydrofolate ligase
NA	HMPRNC0000_1876	*acsA2*		-1		-1				Acetyl-CoA synthetase
NA	HMPRNC0000_1879	*acuC*						-1		NAD-independent protein deacetylase AcuC
NA	HMPRNC0000_1881	*aroF*				1				Chorismate mutase I / 2-keto-3-deoxy-D-arabino-heptulosonate-7-phosphate synthase I beta
NA	HMPRNC0000_1882	.			-1		-1			hypothetical protein
NA	HMPRNC0000_1883	.			-1		-1			hypothetical protein
NA	HMPRNC0000_1884	.			-1		-1			hypothetical protein
NA	HMPRNC0000_1902	.		1						Rhodanese-like domain protein
NA	HMPRNC0000_1906	.		1						Putative rRNA methylase YtqB
NA	HMPRNC0000_1907	*rot*		-1		-1				Repressor of toxins Rot
NA	HMPRNC0000_1909	*putA*		-1	-1	-1	-1			Proline dehydrogenase
NA	HMPRNC0000_1911	*ribB*						1		3,4-dihydroxy-2-butanone 4-phosphate synthase / GTP cyclohydrolase II
NA	HMPRNC0000_1914	.						-1		Hypothetical protein
NA	HMPRNC0000_1935	.		-1		-1				Hypothetical protein
NA	HMPRNC0000_1936	.		-1		-1				Hypothetical protein
NA	HMPRNC0000_1937	.		-1						Hypothetical protein
NA	HMPRNC0000_1939	.		-1		-1				Hypothetical protein
NA	HMPRNC0000_1947	.				-1				Type I restriction-modification system, specificity subunit S
NA	HMPRNC0000_1980	.						-1		ABC transporter, ATP-binding protein EcsA
NA	HMPRNC0000_1982	.			-1		-1			Hypothetical protein
NA	HMPRNC0000_1984	*prsA2*		-1		-1				Foldase protein PrsA precursor and Foldase clustered with pyrimidine conversion
NA	HMPRNC0000_2004	.		1		1				ABC transporter/substrate-binding protein/ABC transporter,permease protein
NA	HMPRNC0000_2049	*yfhP*							1	conserved membrane protein YfhP
NA	HMPRNC0000_2062	.		-1						Acyl-CoA hydrolase
NA	HMPRNC0000_2066	.		-1	-1		-1			Hypothetical protein
NA	HMPRNC0000_2067	*yihY*		-1						Inner membrane protein YihY, formerly thought to be RNase BN
NA	HMPRNC0000_2069	*vraS*						-1		Sensor histidine kinase VraS
NA	HMPRNC0000_2075	.			1		1			Putative amidotransferase similar to cobyric acid synthase
NA	HMPRNC0000_2076	*murE*			1		1			Putative amino acid ligase found clustered with an amidotransferase
NA	HMPRNC0000_2084	*gatB*			1		1			Aspartyl-tRNA(Asn) amidotransferase subunit B and Glutamyl-tRNA(Gln) amidotransferase subunit B
NA	HMPRNC0000_2086	*gatC*						-1		Aspartyl-tRNA(Asn) amidotransferase subunit C and Glutamyl-tRNA(Gln) amidotransferase subunit C
NA	HMPRNC0000_2092	*purB*		-1		-1				Adenylosuccinate lyase and SAICAR lyase
NA	HMPRNC0000_2104	*sdcS*		-1						Sodium-dependent anion transporter family
NA	HMPRNC0000_2128	*mapW2*			-1		-1			Extracellular adherence protein of broad specificity Eap/Map
NA	HMPRNC0000_2132	*scn*		1						Fibrinogen binding phage associated protein
NA	HMPRNC0000_2135	*lytN*		1						Phage lysin, N-acetylmuramoyl-L-alanine amidase
NA	HMPRNC0000_2191	.						1		Cytolytic pore-forming protein S component -> Leukocidin LukS-PV
NA	HMPRNC0000_2202	.		-1		-1				Hypothetical protein
NA	HMPRNC0000_2204	.		1		1				Nitroreductase family protein
NA	HMPRNC0000_2229	*yeaZ*						-1		TRNA threonylcarbamoyladenosine biosynthesis protein TsaB
NA	HMPRNC0000_2235	*ilvC*		1		1				Ketol-acid reductoisomerase (NADP(+))
NA	HMPRNC0000_2243	.							1	Mobile element protein
NA	HMPRNC0000_2268	*deaD*			1		1			DEAD-box ATP-dependent RNA helicase DeaD
NA	HMPRNC0000_2280	*yidC*				-1				Inner membrane protein translocase and chaperone YidC
NA	HMPRNC0000_2285	*tenA*		-1		-1				Thiaminease II involved in salvage of thiamine pyrimidine moiety
NA	HMPRNC0000_2300	*atpE*		-1		-1				ATP synthase F0 sector subunit c
NA	HMPRNC0000_2302	.		-1		-1				Uncharacterized ncRNA
NA	HMPRNC0000_2306	*ywlG*		-1	-1		-1			UPF0340 protein YwlG
NA	HMPRNC0000_2319	.		1		1				Transcriptional regulator, HxlR family
NA	HMPRNC0000_2332	.		1		1				Hypothetical protein
NA	HMPRNC0000_2349	*czrA*		1		1		1		Zn(II) or Co(II)-specific transcriptional repressor protein
NA	HMPRNC0000_2351	.						-1		Lytic regulatory protein
NA	HMPRNC0000_2358	*mtlA*						1		PTS system, mannitol-specific IIC component / PTS system, mannitol-specific IIB component

NA	HMPRNC0000_2363	*fmtB1*				1				FmtB (Mrp) protein involved in methicillin resistance and cell wall biosynthesis
NA	HMPRNC0000_2370	*rocF*		-1						Arginase
NA	HMPRNC0000_2384	*sepA*						-1		Uncharacterized ncRNA
NA	HMPRNC0000_2387	.						-1		N-acetylglucosamine-1-phosphate uridyltransferase eukaryotic
NA	HMPRNC0000_2389	.						1		Hypothetical protein
NA	HMPRNC0000_2401	*asp*		-1	-1		-1			Alkaline shock protein 23
NA	HMPRNC0000_2403	.			-1		-1			Hypothetical protein
NA	HMPRNC0000_2405	.		1		1				Bifunctional protein: zinc-containing alcohol dehydrogenase, quinone oxidoreductase (NADPH:quinone reductase), Similar to arginate lyase
NA	HMPRNC0000_2407	.			1		1			Cell surface hydrolase (putative)
NA	HMPRNC0000_2409	*lacG*		-1				-1		6-phospho-beta-galactosidase
NA	HMPRNC0000_2471	*glcU*		1						Glucose:proton symporter GlcU
NA	HMPRNC0000_2473	.						-1		Hypothetical protein
NA	HMPRNC0000_2485	*sarV*		1		1				Transcriptional regulator SarV (accessory regulator V)
NA	HMPRNC0000_2528	*ssaA*		-1		-1				Secretory antigen precursor SsaA
NA	HMPRNC0000_2538	.			-1		-1			hypothetical protein
NA	HMPRNC0000_2539	*yjgC*			-1		-1			putative oxidoreductase YjgC
NA	HMPRNC0000_2545	.		1		1				Transcriptional regulator, DeoR family
NA	HMPRNC0000_2547	.		-1				-1		Protein of pXO2-46
NA	HMPRNC0000_2564	.		1						Hypothetical protein
NA	HMPRNC0000_2566	.			-1		-1			hypothetical protein
NA	HMPRNC0000_2569	*hipO*		1						N-acetyl-L,L-diaminopimelate deacetylase
NA	HMPRNC0000_2570	*hutI*		-1		-1				Imidazolonepropionase
NA	HMPRNC0000_2572	*hutU*		-1		-1				Urocanate hydratase
NA	HMPRNC0000_2574	*hutG*			-1		-1			Formiminoglutamase
NA	HMPRNC0000_2581	.		-1						ABC-type Na+ efflux pump, permease component
NA	HMPRNC0000_2582	.		-1						ABC transporter, ATP-binding protein
NA	HMPRNC0000_2615	.						-1		Uncharacterized ncRNA
NA	HMPRNC0000_2633	.		-1	-1	-1	-1		1	Putative type II DNA modification enzyme
NA	HMPRNC0000_2660	*cbiX*		-1	-1	-1	-1			Sirohydrochlorin ferrochelatase SirB
NA	HMPRNC0000_2669	.				1				Hypothetical protein
NA	HMPRNC0000_2673	.		1	1	1	1			L-cystine ABC transporter, ATP-binding protein TcyC
NA	HMPRNC0000_2674	.				1				Transcript
NA	HMPRNC0000_2685	*sbi*		1					1	IgG-binding protein SBI
NA	HMPRNC0000_2687	*hlgA*		1	-1	1	-1			Cytolytic pore-forming protein S component -> Gamma-hemolysin HlgA
NA	HMPRNC0000_2689	*lukS*							1	Cytolytic pore-forming protein S component -> Gamma-hemolysin HlgC
NA	HMPRNC0000_2691	*hlgB*		1	-1	1	-1			Cytolytic pore-forming protein F component -> Gamma-hemolysin HlgB
NA	HMPRNC0000_2708	.		1						Hypothetical protein
NA	HMPRNC0000_2711	.				1				Na+/H+ antiporter
NA	HMPRNC0000_2714	.		1						Hypothetical protein
NA	HMPRNC0000_2715	.		1						Uncharacterized ncRNA
NA	HMPRNC0000_2717	.		1		1				MFS-type transporter
NA	HMPRNC0000_2722	*proV*						1		Choline ABC transport system, ATP-binding protein OpuBA
NA	HMPRNC0000_2724	.		1	1	1	1			Amino acid-proton symporter YbeC
NA	HMPRNC0000_2725	*pnbA*		-1	-1	-1	-1			Carboxylesterase, type B
NA	HMPRNC0000_2756	.			-1		-1			hypothetical protein
NA	HMPRNC0000_2759	*pgm*		-1		-1				Phosphoglucosamine mutase / Phosphomannomutase
NA	HMPRNC0000_2765	*sasG*						-1	1	Virulence-associated cell-wall-anchored protein SasG
NA	HMPRNC0000_2769	*fnbB*		1		1		1		Fibronectin binding protein FnbB
NA	HMPRNC0000_2770	*fnbA*		1	1	1	1			Fibronectin binding protein FnbA
NA	HMPRNC0000_2774	.			-1		-1	-1		Transcriptional regulator, MerR family
NA	HMPRNC0000_2776	.			-1		-1			hypothetical protein
NA	HMPRNC0000_2786	.		-1						Efflux ABC transporter, ATP-binding protein
NA	HMPRNC0000_2787	.		-1		-1				Putative membrane-spanning protein
NA	HMPRNC0000_2791	.		1		1				Phospholipase/carboxylesterase
NA	HMPRNC0000_2792	.		1		1				Glyoxalase family protein
NA	HMPRNC0000_2793	.		1		1				Transcriptional regulator, MarR family
NA	HMPRNC0000_2795	.		1		1				Glyoxalase family protein
NA	HMPRNC0000_2804	*srtA*		1						LPXTG specific sortase A
NA	HMPRNC0000_2814	.		1		1				Thioredoxin
NA	HMPRNC0000_2815	.		1		1				Hypothetical protein
NA	HMPRNC0000_2824	*mvaS*				-1				Hydroxymethylglutaryl-CoA synthase
NA	HMPRNC0000_2828	*clpL*			-1		-1			ClpE family protein
NA	HMPRNC0000_2834	*rocA*		-1		-1				Delta-1-pyrroline-5-carboxylate dehydrogenase
NA	HMPRNC0000_2839	*copZ*		1						Copper(I) chaperone CopZ
NA	HMPRNC0000_2842	*crtN*		-1					-1	Dehydrosqualene desaturase
NA	HMPRNC0000_2843	*crtM*							-1	Dehydrosqualene synthase
NA	HMPRNC0000_2844	.		-1	-1		-1			4,4'-diaponeurosporenoate glycosyltransferase
NA	HMPRNC0000_2846	*crtI*		-1						Diapolycopene oxygenase
NA	HMPRNC0000_2849	.				-1				Acyltransferase
NA	HMPRNC0000_2852	*isaA*				-1				Immunodominant antigen A precursor
NA	HMPRNC0000_2856	.		1	1	1	1			Hypothetical protein
NA	HMPRNC0000_2857	.		1	1	1	1	1	1	Hypothetical protein
NA	HMPRNC0000_2865	.			-1		-1			Putative hydrolase/acyltransferase

NA	HMPRNC0000_2891	.		1		1		-1	-1	Amino acid permease, GabP family
NA	HMPRNC0000_2898	.			-1		-1			hypothetical protein
NA	HMPRNC0000_2918	.		-1						ABC transporter sensor linked response regulator
NA	HMPRNC0000_2924	.						-1		Cardiolipin synthetase
NA	HMPRNC0000_2926	*clfB*			-1		-1	-1	-1	Clumping factor ClfB, fibrinogen binding protein
NA	HMPRNC0000_2942	*manA*						-1		PTS system, mannose-specific IIB/IIC/IIA component
NA	HMPRNC0000_2949	*sraP*				1				Putative cell-wall-anchored protein SasF (LPXAG motif)
NA	HMPRNC0000_2966	.			-1		-1			hypothetical protein
NA	HMPRNC0000_2986	*hisB*				1				Imidazoleglycerol-phosphate dehydratase
NA	HMPRNC0000_2987	*hisC*		1		1				Histidinol-phosphate aminotransferase
NA	HMPRNC0000_2990	*hisG*		1		1				ATP phosphoribosyltransferase -> HisGs
NA	HMPRNC0000_2991	*hisZ*		1		1				ATP phosphoribosyltransferase regulatory subunit
NA	HMPRNC0000_2996	.				1				Duplicated ATPase component MtsB of energizing module of methionine-regulated ECF transporter
NA	HMPRNC0000_2999	.				1				Hypothetical protein
NA	HMPRNC0000_3011	.						1		Hypothetical protein
NA	HMPRNC0000_3019	*cspB*						-1		Cold shock protein of CSP family
NA	HMPRNC0000_3025	.		1	1		1			Chromosome (plasmid) partitioning protein ParB-2
