# Supplementary material for: The Effect of Three Complexes of Iodine with Amino Acids on Gene Expression of Model Antibiotic Resistant Microorganisms Escherichia coli ATCC BAA-196 and Staphylococcus aureus ATCC BAA-39
Source: Microorganisms. 2023 Jun 29;11(7):1705. doi: 10.3390/microorganisms11071705 (PMC10383335; doi:10.3390/microorganisms11071705)
Supplement: Supplementary file 1 [file microorganisms-11-01705-s001.zip › Table S3.pdf]

Table S3. Regulation of expression of genes involved in the central metabolism of bacteria by the three tested complexes.

Locus tag in *E. coli* BAA-196	Locus tag in *S. aureus* BAA-39	Gene	*E.coli* BAA-196						*S.aureus* BAA-39						Annotation
			IPC25		IPC33		IPC51		IPC25		IPC33		IPC51		
			Lag	Log	Lag	Log	Lag	Log	Lag	Log	Lag	Log	Lag	Log	
**Glycolysis**															
BAA196NC_4208	HMPRNC0000_0863	*tpiA*							1		1			-1	Triosephosphate isomerase
NA	HMPRNC0000_0860	*gapA1*							1					-1	NAD-dependent glyceraldehyde-3-phosphate dehydrogenase
**Glycolysis & Gluconeogenesis**															
BAA196NC_0782	HMPRNC0000_0861	*pgk*							1	1		1			Phosphoglycerate kinase
BAA196NC_3009	HMPRNC0000_2679	*gpmA*						1					-1		Phosphoglyceromutase
**Gluconeogenesis**															
BAA196NC_0089	HMPRNC0000_0594	*kbl*							-1						2-amino-3-ketobutyrate coenzyme A ligase
BAA196NC_1920	HMPRNC0000_1815	*gapA*							-1		-1				Glyceraldehyde-3-phosphate dehydrogenase
BAA196NC_0316	HMPRNC0000_1928	*pck*		1			-1		-1		-1				Phosphoenolpyruvate carboxykinase
BAA196NC_0477	HMPRNC0000_2889	*mdh*		1									1		Malate dehydrogenase
BAA196NC_1977	HMPRNC0000_2415	*pfkB*							-1		-1				6-phosphofructokinase II
BAA196NC_1238	NA	*maeB*	1	1		1									Malic enzyme
NA	HMPRNC0000_1834	*maeB*							-1						NADP-dependent malic enzyme
NA	HMPRNC0000_0771	*pfkB2*							1	1	1	1			1-phosphofructokinase
**Entner–Doudoroff (ED) pathway**															
BAA196NC_1997	NA	*pps*		1	1	1									Phosphoenolpyruvate synthase
**Pentose Phosphate Pathway (PPP)**															
BAA196NC_1844	HMPRNC0000_1624	*zwf*									1			1	Glucose-6-phosphate 1-dehydrogenase
BAA196NC_1237	NA	*talA*	1	1											Transaldolase A
BAA196NC_0774	NA	*tktA*	1	1											Transketolase 1, thiamin-binding
BAA196NC_1236	HMPRNC0000_1439	*tktB*	1	1											Transketolase 2, thiamin-binding
**Glycogen synthesis**															
BAA196NC_0290	NA	*glgA*					-1								Glycogen synthase
BAA196NC_0664	NA	*glgS*		-1											Glycogen synthesis protein GlgS
NA	HMPRNC0000_2759	*pgm*							-1		-1				Phosphoglucosamine mutase / Phosphomannomutase
BAA196NC_1017	NA	*csrA*	-1	-1	-1	-1									Carbon storage regulator
**TCA**															
BAA196NC_2092	HMPRNC0000_1996	*fumC*							-1		-1				Fumarate hydratase
BAA196NC_2565	HMPRNC0000_1825	*icd*		1		1	-1		-1		-1			1	Isocitrate dehydrogenase
BAA196NC_3030	HMPRNC0000_1313	*sucD*		1				-1	-1		-1				Succinyl-CoA synthetase subunit alpha
BAA196NC_3031	HMPRNC0000_1312	*sucC*		1	-1		-1	-1	-1		-1				Succinyl-CoA synthetase subunit beta
BAA196NC_3032	HMPRNC0000_1519	*sucB*		1	-1		-1	-1	-1		-1				Dihydrolipoamide acetyltransferase
BAA196NC_3033	HMPRNC0000_1520	*sucA*		1			-1	-1	-1		-1				Alpha-ketoglutarate decarboxylase
NA	HMPRNC0000_0644	*merA2*							1	1	1	1			Putative Dihydrolipoamide dehydrogenase/Mercuric ion reductase
**TCA & glyoxylate bypass**															
BAA196NC_0477	HMPRNC0000_2889	mdh	1	1									1		Malate dehydrogenase
BAA196NC_2425	HMPRNC0000_1449	*acnA*		1					-1		-1				Aconitate hydratase
BAA196NC_3038	NA	*gltA*		1											Citrate synthase
BAA196NC_3620	NA	*acnB*		1											Aconitate hydratase
**Glyoxylate bypass**															
BAA196NC_4123	NA	*aceA*		1		1									Isocitrate lyase
BAA196NC_4124	NA	*aceB*		1		1		-1							Malate synthase
**Aerobic respiration**															
BAA196NC_3034	HMPRNC0000_1198	*sdhB*		1			-1	-1	-1		-1			1	Succinate dehydrogenase, FeS subunit
BAA196NC_3035	HMPRNC0000_1197	*sdhA*		1		1	-1	-1	-1		-1			1	Succinate dehydrogenase flavoprotein subunit
BAA196NC_3036	NA	*sdhD*					-1	-1							Succinate dehydrogenase cytochrome b556 small membrane subunit
BAA196NC_3037	HMPRNC0000_1195	*sdhC*				-1		-1	-1						Succinate dehydrogenase cytochrome b556 large membrane subunit
BAA196NC_3323	NA	*cyoA*					-1								Cytochrome o ubiquinol oxidase subunit II
BAA196NC_3324	HMPRNC0000_1094	*cyoB*	1	1			-1								Cytochrome o ubiquinol oxidase subunit I
BAA196NC_3325	NA	*cyoC*					-1								Cytochrome o ubiquinol oxidase subunit III
BAA196NC_3326	NA	*cyoD*					-1								Cytochrome o ubiquinol oxidase subunit IV
BAA196NC_3327	HMPRNC0000_1158	*cyoE*	1	1											Protoheme IX farnesyltransferase
BAA196NC_3025	HMPRNC0000_1124	*cydA*								1		1			Cytochrome d terminal oxidase, subunit I
BAA196NC_3024	NA	*cydB*					-1								Cytochrome d terminal oxidase, subunit II
NA	HMPRNC0000_1095	*qoxA*												-1	Cytochrome aa3-600 menaquinol oxidase subunit II
NA	HMPRNC0000_1093	*qoxD*							-1	-1		-1			Cytochrome aa3-600 menaquinol oxidase subunit IV
BAA196NC_1411	NA	*nuoC*						-1							NADH:ubiquinone oxidoreductase, chain C,D
BAA196NC_1412	NA	*nuoE*						-1							NADH dehydrogenase subunit E
BAA196NC_1414	NA	*nuoG*						-1							NADH dehydrogenase subunit G
BAA196NC_2594	HMPRNC0000_0966	*ndh*						1							Respiratory NADH dehydrogenase 2/cupric reductase
**Anaerobic respiration & fermentation**															
BAA196NC_2425	HMPRNC0000_1449	*acnA*	1	1					-1		-1				Aconitate hydratase
BAA196NC_3620	NA	*acnB*	1	1											Aconitate hydratase
BAA196NC_2473	HMPRNC0000_2652	*narH*					-1		-1		-1				Nitrate reductase 1, beta (Fe-S) subunit
BAA196NC_2474	HMPRNC0000_2655	*narG*	1	1		1		1	-1		-1			-1	Nitrate reductase 1, alpha subunit
BAA196NC_2475	HMPRNC0000_2644	*narK*						1	-1		-1				Nitrate/nitrite transporter
BAA196NC_2478	HMPRNC0000_2068	*narL*						1							DNA-binding response regulator in two-component regulatory system with NarX
BAA196NC_2233	NA	*narZ*						-1							Nitrate reductase 2 (NRZ), alpha subunit
BAA196NC_2234	NA	*narY*						-1							Nitrate reductase 2 (NRZ), beta subunit
BAA196NC_2477	NA	*narX*						1							Sensory histidine kinase in two-component regulatory system with NarL
BAA196NC_0353	HMPRNC0000_2659	*nirB*						1	-1		-1				Nitrite reductase, large subunit, NAD(P)H-binding
BAA196NC_1495	NA	*napA*					-1								Nitrate reductase, periplasmic, large subunit

BAA196NC_1496	NA	*napG*						-1							Quinol dehydrogenase periplasmic component
BAA196NC_1499	NA	*napC*					-1								Nitrate reductase, cytochrome c-type, periplasmic
BAA196NC_2027	NA	*fumD*		-1		-1									Fumarase D
BAA196NC_3977	HMPRNC0000_1197	*frdA*					-1	-1	-1		-1			1	Fumarate reductase
BAA196NC_3978	HMPRNC0000_1198	*frdB*					-1	-1	-1		-1			1	Fumarate reductase (anaerobic), Fe-S subunit
BAA196NC_4234	NA	*fdoG*	1	1	1			-1							Formate dehydrogenase-O, large subunit
BAA196NC_4235	NA	*fdoH*					-1								Formate dehydrogenase-O, Fe-S subunit
BAA196NC_4236	NA	*fdoI*	1	1											Formate dehydrogenase-O, cytochrome b556 subunit
BAA196NC_4237	NA	*fdhE*						-1							Formate dehydrogenase accessory protein FdhE
BAA196NC_0120	HMPRNC0000_0169	*aldB*					-1	-1		1		1			Aldehyde dehydrogenase B
NA	HMPRNC0000_0860	*gapA1*							1					-1	NAD-dependent glyceraldehyde-3-phosphate dehydrogenase
BAA196NC_0094	HMPRNC0000_0864	*gpmI*							1	1	1	1			Phosphoglyceromutase
BAA196NC_3009	HMPRNC0000_2679	*gpmA*						1					-1		Phosphoglyceromutase
**Acetate & pyruvate metabolism**															
BAA196NC_1400	HMPRNC0000_1846	*ackA*								1		1			Acetate kinase
BAA196NC_1399	NA	*pta*			1	-1		-1							Phosphate acetyltransferase
BAA196NC_0596	NA	*tdcD*				-1		-1							Propionate kinase/acetate kinase C, anaerobic
BAA196NC_0597	NA	*tdcE*					-1	-1							Pyruvate formate-lyase 4/2-ketobutyrate formate-lyase
BAA196NC_2797	HMPRNC0000_0241	*pflB*					-1								Pyruvate formate lyase I
NA	HMPRNC0000_0178	.							1		1				Acyl-CoA dehydrogenase, short-chain specific
BAA196NC_4065	HMPRNC0000_1877	*acs*	1	1							-1				Acetyl-CoA synthetase
NA	HMPRNC0000_1876	*acsA2*							-1		-1				Acetyl-CoA synthetase
**Peptidoglycan metabolism associated with cell growth and devision**															
BAA196NC_3652	HMPRNC0000_1230	*ftsI*				1		-1	-1						Transpeptidase involved in septal peptidoglycan synthesis (penicillin-binding protein 3)
BAA196NC_3647	HMPRNC0000_1154	*ftsW*	-1	-1		-1									Integral membrane protein involved in stabilizing FstZ ring during cell division
BAA196NC_1917	NA	*mipA*		1		1									scaffolding protein for murein synthesizing machinery
BAA196NC_0969	NA	*nlpD*		1		1									Predicted outer membrane lipoprotein
BAA196NC_2519	NA	*dadA*				1									D-amino acid dehydrogenase small subunit
BAA196NC_2518	NA	*dadX*		1		1									alanine racemase
BAA196NC_2883	NA	*ybiS*					1								L,D-transpeptidase IdtB
BAA196NC_3530	NA	*mltD*			-1		1								Predicted membrane-bound lytic murein transglycosylase D
BAA196NC_2025	NA	*lpp*			-1	-1	1	-1							Murein lipoprotein
BAA196NC_1524	NA	*spr (mepS)*					1								Predicted peptidase, outer membrane lipoprotein
BAA196NC_3117	NA	*dacA*					1	-1							D-alanyl-D-alanine carboxypeptidase (penicillin-binding protein 5)
BAA196NC_4381	HMPRNC0000_0521	*glmU*					1								Bifunctional N-acetylglucosamine-1-phosphate uridyltransferase/glucosamine-1-phosphate acetyltransferase
**KEGG predicted Peptidoglycan synthesis**															
BAA196NC_0523	HMPRNC0000_2292	*murA1*					1								UDP-N-acetylglucosamine 1-carboxyvinyltransferase
NA	HMPRNC0000_2320	*murA2*													UDP-N-acetylglucosamine 1-carboxyvinyltransferase
BAA196NC_4159	HMPRNC0000_0815	*murB*													UDP-N-acetylenolpyruvoylglucosamine reductase
BAA196NC_3645	HMPRNC0000_1885	*murC*					1	-1						-1	UDP-N-acetylmuramate--L-alanine ligase
BAA196NC_3648	HMPRNC0000_1232	*murD*							-1		-1				UDP-N-acetylmuramoylalanine--D-glutamate ligase
NA	HMPRNC0000_2076	*murE*								1		1			putative amino acid ligase found clustered with an amidotransferase
BAA196NC_3650	HMPRNC0000_2269	*murF*													UDP-N-acetylmuramoyl-tripeptide--D-alanyl-D- alanine ligase
BAA196NC_3649	HMPRNC0000_1231	*mraY*							-1		-1				Phospho-N-acetylmuramoyl-pentapeptide- transferase
NA	HMPRNC0000_1526	*murG*													UDP-N-acetylglucosamine--N-acetylmuramyl- (pentapeptide) pyrophosphoryl-undecaprenol N-acetylglucosamine transferase
NA	HMPRNC0000_2479	*femX*													Lipid II:glycine glycyltransferase
NA	HMPRNC0000_1475	*femA*													tRNA-dependent lipid II-Gly glycyltransferase and FemA
NA	HMPRNC0000_1476	*femB*													tRNA-dependent lipid II-GlyGlyGly glycyltransferase
NA	HMPRNC0000_1872	*sgtA*													Multimodular transpeptidase-transglycosylase
NA	HMPRNC0000_1560	*pbp1*								1		1			Multimodular transpeptidase-transglycosylase / Penicillin-binding protein 1A/1B (PBP1)
BAA196NC_4160	HMPRNC0000_1199	*murI*													Glutamate racemase
